# Dietary DHA supplementation causes selective changes in phospholipids from different brain regions in both wild type mice and the Tg2576 mouse model of Alzheimer's disease

**DOI:** 10.1016/j.bbalip.2016.03.005

**Published:** 2016-06

**Authors:** Cécile Bascoul-Colombo, Irina A. Guschina, Benjamin H. Maskrey, Mark Good, Valerie B. O'Donnell, John L. Harwood

**Affiliations:** a School of Biosciences, Cardiff University, Cardiff CF10 3AX, UK; b School of Psychology, Cardiff University, Cardiff CF10 3AT, UK; c School of Medicine, Cardiff University, Cardiff CF14 4XN, UK

**Keywords:** Alzheimer's disease, 2576 mouse model, Phospholipid lipidomics, DHA-enriched diet, Brain areas

## Abstract

Alzheimer's disease (AD) is of major concern in ageing populations and we have used the Tg2576 mouse model to understand connections between brain lipids and amyloid pathology. Because dietary docosahexaenoic acid (DHA) has been identified as beneficial, we compared mice fed with a DHA-supplemented diet to those on a nutritionally-sufficient diet.

Major phospholipids from cortex, hippocampus and cerebellum were separated and analysed. Each phosphoglyceride had a characteristic fatty acid composition which was similar in cortex and hippocampus but different in the cerebellum. The biggest changes on DHA-supplementation were within ethanolamine phospholipids which, together with phosphatidylserine, had the highest proportions of DHA. Reciprocal alterations in DHA and arachidonate were found. The main diet-induced alterations were found in ethanolamine phospholipids, (and included their ether derivatives), as were the changes observed due to genotype. Tg mice appeared more sensitive to diet with generally lower DHA percentages when on the standard diet and higher relative proportions of DHA when the diet was supplemented. All four major phosphoglycerides analysed showed age-dependent decreases in polyunsaturated fatty acid contents.

These data provide, for the first time, a detailed evaluation of phospholipids in different brain areas previously shown to be relevant to behaviour in the Tg2576 mouse model for AD. The lipid changes observed with genotype are consistent with the subtle alterations found in AD patients, especially for the ethanolamine phospholipid molecular species. They also emphasise the contrasting changes in fatty acid content induced by DHA supplementation within individual phospholipid classes.

## Highlights

• The effects of dietary DHA on brain phospholipids were studied in normal and Tg2576 mice.
• Different lipids were affected variously by diet with PE being the most altered.
• Dietary DHA reduced brain arachidonate in PE, PC, PS and even in PI that contained little DHA.
• Genotype changes were mainly found in ethanolamine phosphoglycerides.
• The genotype data are consistent with results reported for Alzheimer's patients.

## 1 Introduction

Lipids are important constituents of brain, representing 50–60% dry weight in adults [1]. Major components are cholesterol and phospholipids (PL) such as phosphatidylcholine (PC), phosphatidylethanolamine (PE), phosphatidylserine (PS), phosphatidylinositol (PI) and sphingomyelin (Sph) [2], [3]. With their high level of docosahexaenoic acid (22:6n−3, DHA), the fatty acid composition of brain PL differs significantly from other tissues. DHA and the major n−6 polyunsaturated fatty acid (PUFA), arachidonic acid (20:4n−6, AA) account for about 8% and 6% of the dry weight of human brain, respectively [4], with DHA representing up to 20% of the total fatty acids. In contrast, other PUFA such as α-linolenic (18:3n−3, ALA) and eicosapentaenoic (20:5n−3, EPA) acids comprise less than 1% of total brain fatty acids [5]. The DHA content of different PL varies considerably and is especially high in PE and PS [6].

A number of studies have investigated the association of n−3 PUFA or DHA levels with Alzheimer's disease (AD) by examining *post-mortem* autopsy brain samples. Reductions in DHA in different PL have been reported by several labs. [7], [8], [9], [10], [11]. Recent studies have identified plasmanyl and plasmenyl derivatives of PL, especially of PE, as being notably reduced in AD brains [12], [13], [14].

In agreement with the above analyses, major epidemiological surveys show that dietary n−3 PUFA and/or DHA significantly reduce the risk of developing AD [15], [16], [17]. Moreover, brain DHA levels are positively associated with cognitive and behavioural performance [18]. Indeed, n−3 PUFA supplementation can improve brain function, especially for complex tasks in normal individuals [19].

To extend observations with humans, animal experiments have been carried out, including studies on rodent models of AD. Dietary deficiency of n−3 PUFA in rodents leads to a concomitant increase in brain n−6 PUFA [20], [21], [22]. Moreover, n−3 PUFA supplementation not only increased PL DHA levels [23], [24], [25], [26], [27], [28] but was shown to improve learning and memory [24], [26], [27], [28]. In a rat AD model caused by amyloid-β (Aβ1–40) injection, DHA and EPA were each shown to be of benefit [29], [30], [31]. In various transgenic mouse models feeding supplementary n−3 PUFA, especially DHA, was associated with lower Aβ levels [10], [11], [32], [33], [34], [35], [36], [37] and such animals showed improved behaviour [35], [36], [37].

The above studies indicate that dietary DHA can be of benefit for AD, possibly by alleviating the amyloid pathology. Therefore, we decided to examine the effects of dietary DHA on brain lipid composition in detail by using the Tg2576 mouse which expresses the Swedish double mutation of the amyloid precursor protein (APP) gene. This was the first transgenic model for an AD mouse line to display cognitive defects and, as such, has contributed greatly to the field [38]. However, despite its widespread usage, a comprehensive examination of the brain PL of the Tg2576 mouse has not been undertaken previously. Moreover, such data would permit comparisons with reported alterations in the brain lipids of AD patients — a useful exercise for such a widely used animal model for cognitive impairment.

Our data on the fatty acid composition of different PL revealed that effects of diet were much greater than for genotype. Moreover, there were notable differences between PL in the response of their compositions to dietary DHA. The main increase in DHA was found in the ethanolamine phospholipids while the other main DHA-enriched PL, PS, showed little change. All brain phosphoglycerides showed decreases in PUFA contents between 12 and 16 months. It was noted that most of the significant genotypic changes in molecular species occurred for ethanolamine lipids, just as reported for AD patients.

## 2 Material and methods

### 2.1 Chemicals

Standard chemicals and solvents of analytical or HPLC grade were purchased from Fisher Scientific (Loughborough, UK) or Sigma (Poole, UK). The dimyristoyl (di-14:0) phospholipid standards, (dimyristoyl-phosphatidylethanolamine (DMPE), dimyristoyl-phosphatidylcholine (DMPC) and dimyristoyl-phosphatidylserine (DMPS)) were obtained from Avanti Polar Lipids Inc. (Alabaster, AL, USA). Other phospholipid standards used for identification were obtained from Sigma (Poole, UK) and fatty acids were from Nu-Chek Prep Inc. (Elysian, MN, USA).

### 2.2 Diets and tissue samples

Mice were housed at 21 °C with a 12 h day/night cycle. Pups were weaned at 5–6 weeks of age and allowed *ad libitum* access to rodent chow and water up to 16 weeks. After this time, they were divided randomly into groups fed either a DHA-enriched or an oil-blend control diet. A standard maintenance rodent chow (Rodent Maintenance Diet RM1; Special Diet Services, Essex, UK) was supplemented with 5% DHA-rich oil (DHASCO, Martek Biosciences Corporation (now DSM) Columbia, MD, USA) or 5% oil-blend (lard, palm oil, olive oil, coconut oil, 3:3:3:1 by wt. to give a fatty acid mix similar to the average UK diet). According to the manufacturer (Special Diet Services, Essex, UK), the supplemented pellets (with the DHA-rich oil or the oil-blend) contained approximately 12.8% protein, 7.2% oil, 3.9% fibre and 5.6% ash and had similar calorific values (3409 kcal/kg DHA diet, 3407 kcal/kg oil-blend diet). The fatty acid content of the DHA-enriched diet and the oil-blend control diet determined by GLC (Section 2.6) is presented in Table 1. The diets were stored in sealed bags at − 20 °C. Analysis of the samples at 6-monthly intervals by GLC did not detect any changes in fatty acid composition or the appearance of oxidative products. Food consumption was monitored in the period 9–12 months and was not significantly different for either diet or genotype.

12, 16 and 21 month-old mice were sacrificed and the brain dissected into cortex, cerebellum and hippocampus. Samples were weighed and snap-frozen in liquid nitrogen and stored at − 80 °C.

### 2.3 Measurement of β-amyloid levels using an enzyme-linked immunosorbent assay

Cortex, hippocampus and cerebellum samples were homogenised in 2% (w/v) sodium dodecyl sulphate (SDS) solution. After centrifugation (21,000 *g* for 1 h), soluble proteins including soluble β-amyloid proteins were contained in the supernatant (SDS extract). Insoluble β-amyloid proteins were extracted from the pellet using ice-cold 5 M guanidine hydrochloride in PBS. The BCA™ Protein Assay kit (Pierce, Rockfore, IL, USA) was used to measure the protein concentration of the samples. β-Amyloid levels were measured by ELISA using commercial colorimetric ELISA kits (BioSource International Inc., Camarillo, CA, USA) for human Aβ1–40 (catalogue number KHB3482) and human Aβ1–42 (catalogue number KHB3442). Samples were analysed in duplicate. Aβ levels were expressed as pg of Aβ per μg of protein, using the protein concentration of the SDS extracts.

### 2.4 Lipid extraction

Lipids were extracted from previously dissected and weighed mouse brain tissue stored at − 80 °C, using the method of Garbus et al. [39]. This is an adaption of the Bligh and Dyer procedure to give quantitative extraction of polar lipids. PL internal standards for MS/MS analysis (DMPE, DMPC and DMPS) were added at the one-phase initial stage. The lipid-containing lower phase of the Garbus extract was washed with fresh upper phase. The tissue residue was extracted a second time by the Garbus et al. [39] method and, after washing (as above), the lower phases were combined. They were taken to dryness under nitrogen and resuspended in chloroform/methanol (2:1, v/v) containing 0.1 mg/ml butylated hydroxytoluene and stored in glass vials at − 20 °C under nitrogen.

### 2.5 Separation of PL by thin layer chromatography

Individual PLs were separated by two-dimensional TLC on silica gel G 60 plates (Merck, Darmstadt, Germany). In order to separate PI from PS, the TLC plates were pre-treated with a solution of 1.2% (w/v) boric acid in ethanol:water (1:1, v/v) [40]. Chromatography used solvent systems adapted from Katyal et al. [41]:

• Solvent system 1: chloroform:methanol:ammonium hydroxide (65:35:10, by volume),
• Solvent system 2: n-butanol:acetic acid:water (90:20:20, by volume).

Plates were then sprayed with 0.2% (w/v) ANSA (8-anilino-1-naphthalene sulphonic acid) in dry methanol and lipids were visualised under UV light (2UV Transilluminator UVP) [40]. The PLs were identified routinely by comparison with phospholipid standards and confirmed using specific colour reagents [42].

### 2.6 Fatty acid analysis

Fatty acid methyl esters (FAMEs) were prepared from individual PL classes separated by TLC, using acid-catalysed transmethylation. They were stored in HPLC grade hexane and separated using a Clarus 500 gas chromatograph (Perkin-Elmer, Norwalk, Connecticut) fitted with a 30 m × 0.25 mm (int. diam) Elite 225 polar capillary column (Perkin-Elmer). The temperature programme used was: initial temperature of 170 °C for 3 min, followed by heating to 220 °C at 4 °C·min^− 1^ and held at 220 °C for 30 min. Samples were injected at a flow rate of 20 ml·min^− 1^ with a split ratio of 20:1 and nitrogen was used as the carrier gas. Pentadecanoic acid was used routinely as an internal standard. Peaks corresponding to the different fatty acids were routinely identified by comparison of their retention times to standards.

### 2.7 Phosphoglyceride analysis by electrospray ionisation tandem mass spectrometry

Individual molecular species of the major phosphoglyceride classes, PE, PC, PS and PI were analysed in mouse brain lipid extracts using reverse-phase high performance liquid chromatography followed by tandem mass spectrometry (RP-LC–MS/MS). Individual phosphoglyceride molecular species were first identified by direct infusion into the electrospray source of a hybrid triple–quadrupole/linear ion trap mass spectrometer (4000 Q-Trap, Applied Biosystems/MDS Sciex, Concord, ON, Canada) at 10 μl/min. Phosphoglyceride molecular species were identified by a mixture of Q1 scans and diagnostic headgroup scans (PE = precursor 196 − and neutral loss 141 +; PC = precursor 184 +; PS = neutral loss 87 −; PI = precursor 241 −). PL were identified by a combination of MS/MS scans in negative mode to identify their *sn* − 1 and − 2 fatty acids and bond types, and by reference to published tables of PL masses (www.lipidmaps.org) [43]. Diagnostic MRM transitions were then calculated for each lipid based on their parent >* sn* − 2 fatty acid in negative ionisation mode. Due to the poor ionisation of PC in negative ion mode, PC was monitored in both positive ion mode, monitoring the [M + H]^+^ > 184 (protonated phosphocholine headgroup) transition, and also in negative ion mode for the [M-CH_3_]^−^ > *sn* − 2 fatty acid transition to confirm molecular identity. Internal MS/MS parameters for each phospholipid class were optimised using the di14:0 internal standards.

**Fig. 1:**
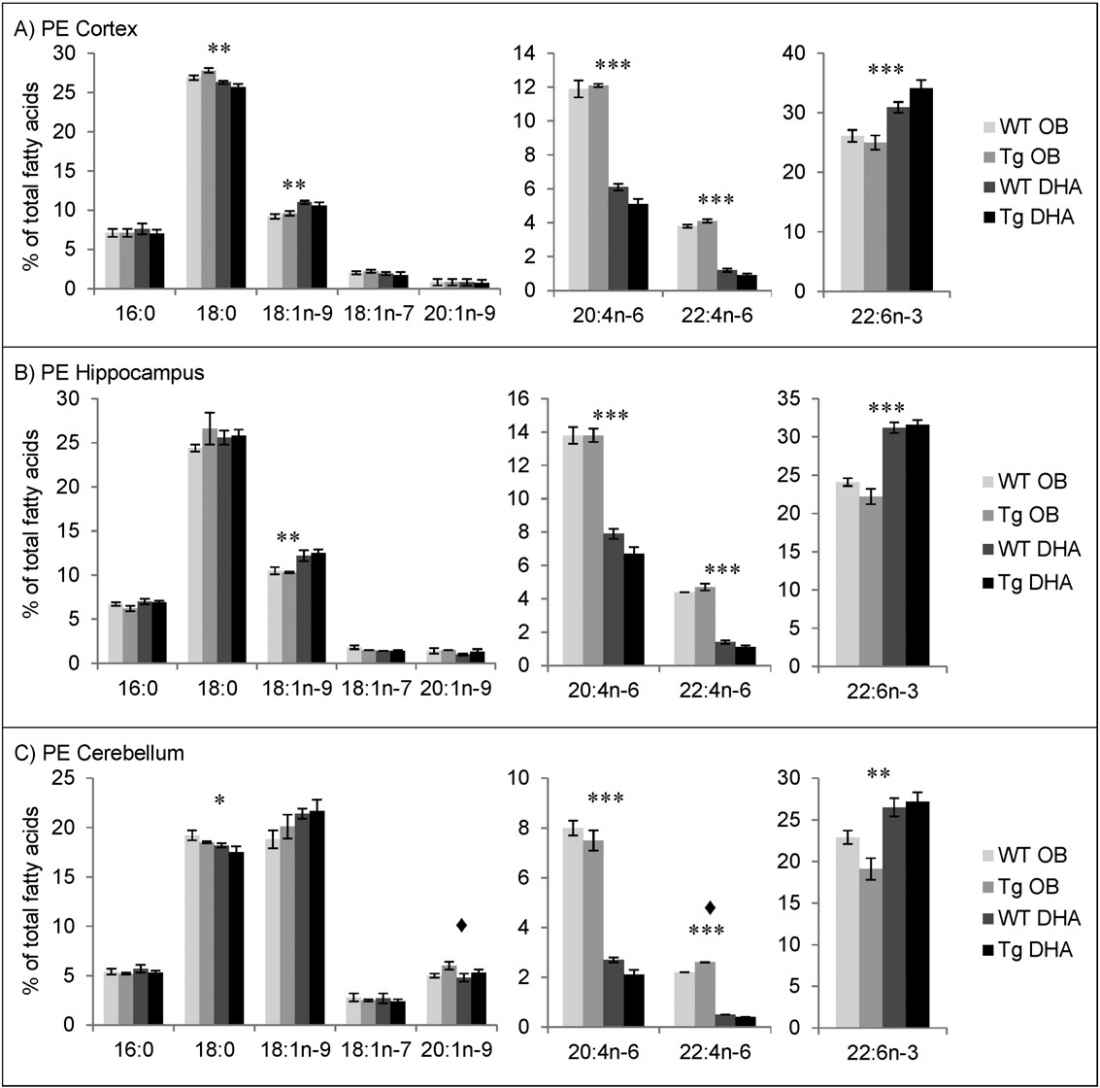
Main fatty acids in ethanolamine phospholipids from cortex (A), hippocampus (B) and cerebellum (C) of 16 month-old wild-type (WT) and transgenic (Tg) mice on the oil blend diet (OB) or on the DHA diet. Results are represented as mean percentages of total fatty acids (Tg OB (n = 3), WT OB (n = 3), Tg DHA (n = 3), WT DHA (n = 3)) ± SEM. Analysis by GLC. Significant effect of diet, *p < 0.05, **p < 0.01, ***p < 0.001; significant effect of genotype, ♦ p < 0.05. The amount of ethanolamine lipids was, on average, 12.09 ± 0.77 (n = 12), 11.48 ± 0.35 (n = 12), 13.40 ± 0.48 (n = 11) μg fatty acids/mg fresh weight for the cortex, hippocampus and cerebellum, respectively. There were no significant differences in the lipid amounts with diet or genotype.

**Fig. 2:**
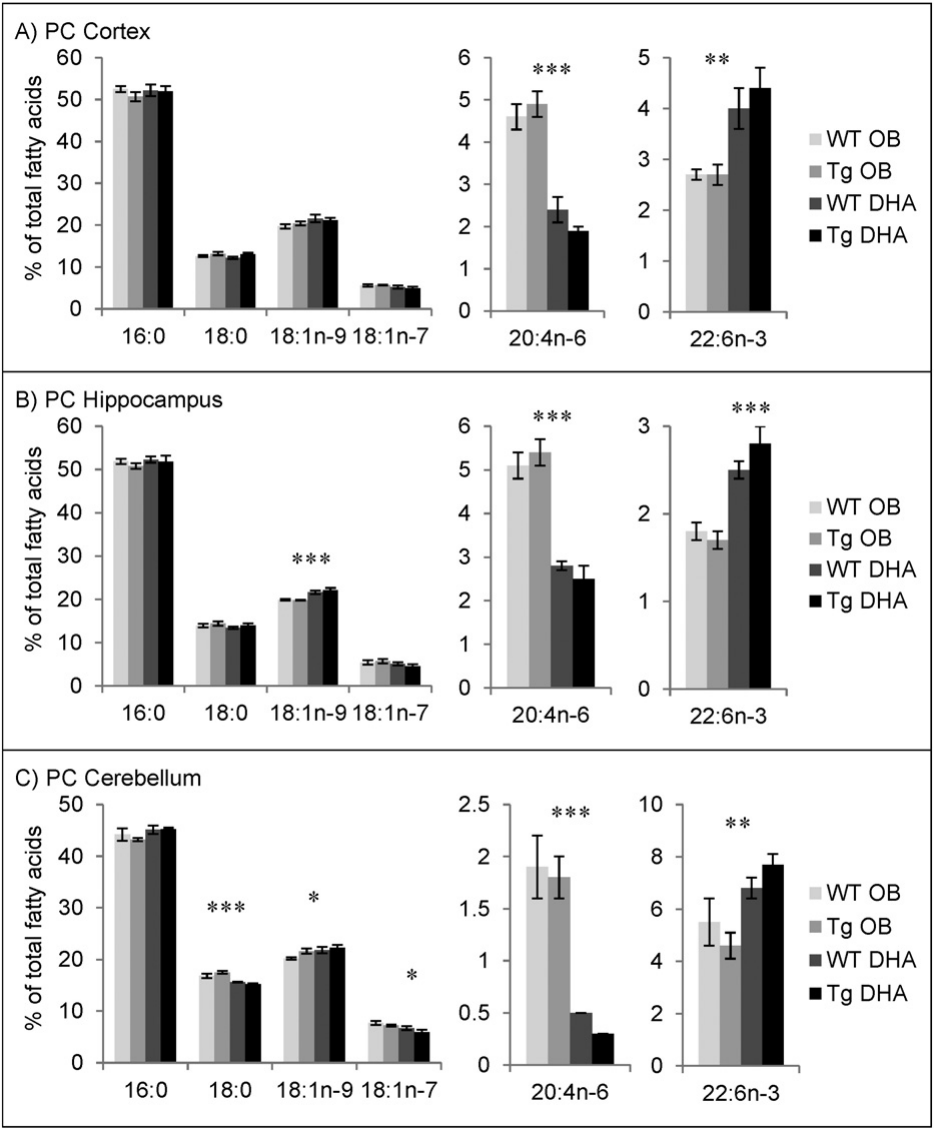
Main fatty acids in choline phospholipids from cortex (A), hippocampus (B) and cerebellum (C) of 16 month-old wild-type (WT) and transgenic (Tg) mice on the oil blend diet (OB) or on the DHA diet. Results are represented as mean percentages of total fatty acids (Tg OB (n = 3), WT OB (n = 3), Tg DHA (n = 3), WT DHA (n = 3)) ± SEM. Analysis by GLC. Significant effect of diet, *p < 0.05, **p < 0.01, ***p < 0.001. The amounts of choline phosphoglycerides were, on average, 13.51 ± 0.84, 13.51 ± 0.33 and 12.54 ± 0.31 μg fatty acids/mg fresh weight for the cortex, hippocampus and cerebellum, respectively. There were no significant differences in the lipid amounts with diet or genotype.

**Fig. 3:**
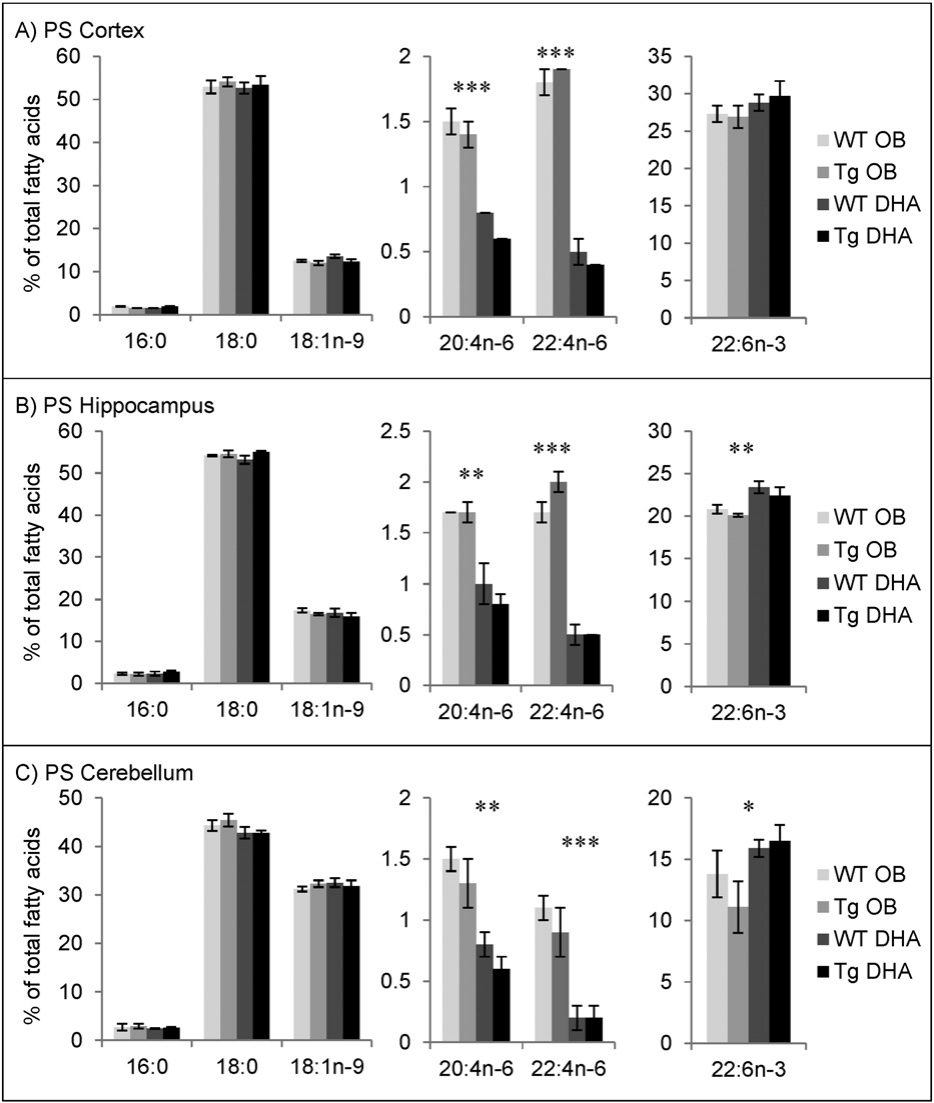
Main fatty acids in phosphatidylserine (PS) from cortex (A), hippocampus (B) and cerebellum (C) of 16 month-old wild-type (WT) and transgenic (Tg) mice on the oil blend diet (OB) or on the DHA diet. Results are represented as mean percentages of total fatty acids (Tg OB (n = 3), WT OB (n = 3), Tg DHA (n = 3), WT DHA (n = 3)) ± SEM. Analysis by GLC. Significant effect of diet, *p < 0.05, **p < 0.01, ***p < 0.001. The amounts of PS were, on average, 5.30 ± 0.20, 5.67 ± 0.49 and 4.99 ± 0.16 μg fatty acids/mg fresh weight for the cortex, hippocampus and cerebellum, respectively. There were no significant differences in the lipid amounts with diet or genotype.

**Fig. 4:**
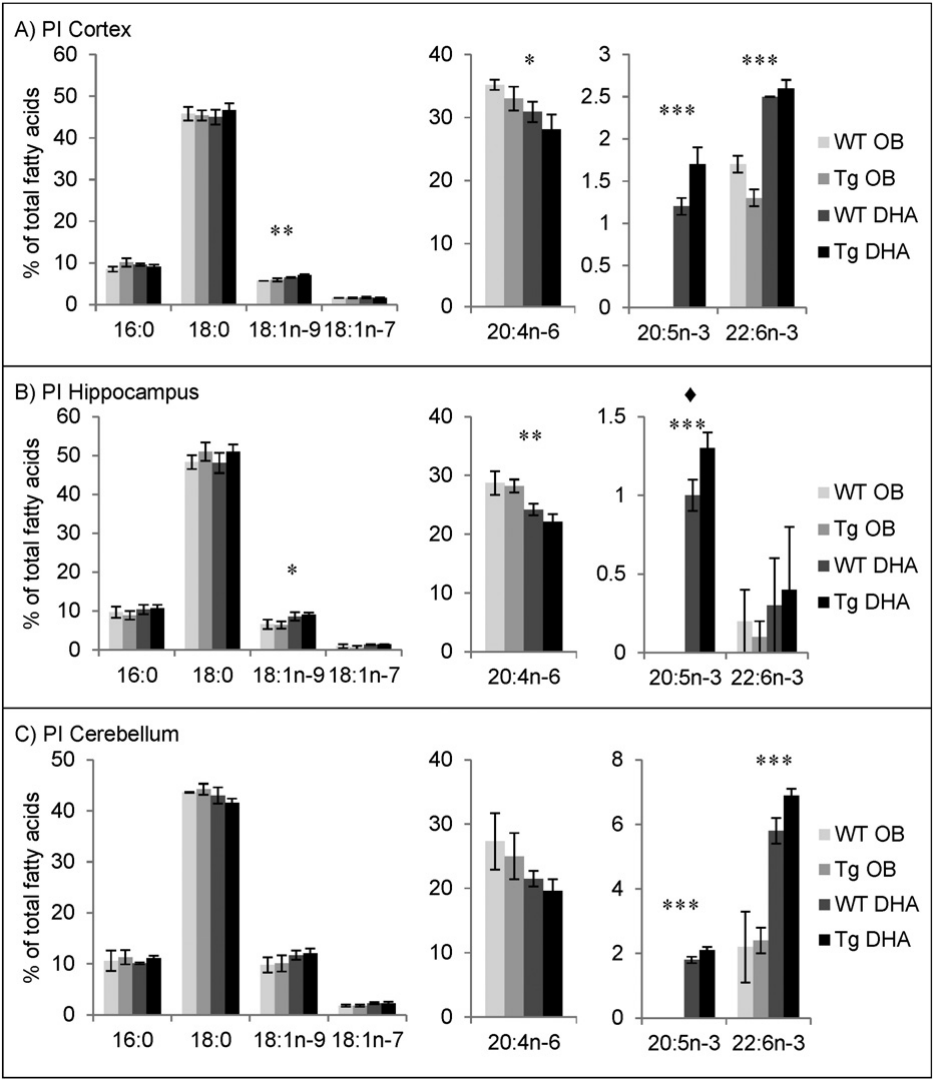
Main fatty acids in phosphatidylinositol (PI) from cortex (A), hippocampus (B) and cerebellum (C) of 16 month-old wild-type (WT) and transgenic (Tg) mice on the oil blend diet (OB) or on the DHA diet. Results are represented as mean percentages of total fatty acids (Tg OB (n = 3), WT OB (n = 3), Tg DHA (n = 3), WT DHA (n = 3)) ± SEM. Analysis by GLC. Significant effect of diet, *p < 0.05, **p < 0.01, ***p < 0.001; significant effect of genotype, ♦ p < 0.05. The amounts of PI were, on average, 1.80 ± 0.09, 1.66 ± 0.14 and 1.48 ± 0.04 μg fatty acids/mg fresh weight for the cortex, hippocampus and cerebellum, respectively. There were no significant differences in the lipid amounts with diet or genotype.

**Fig. 5:**
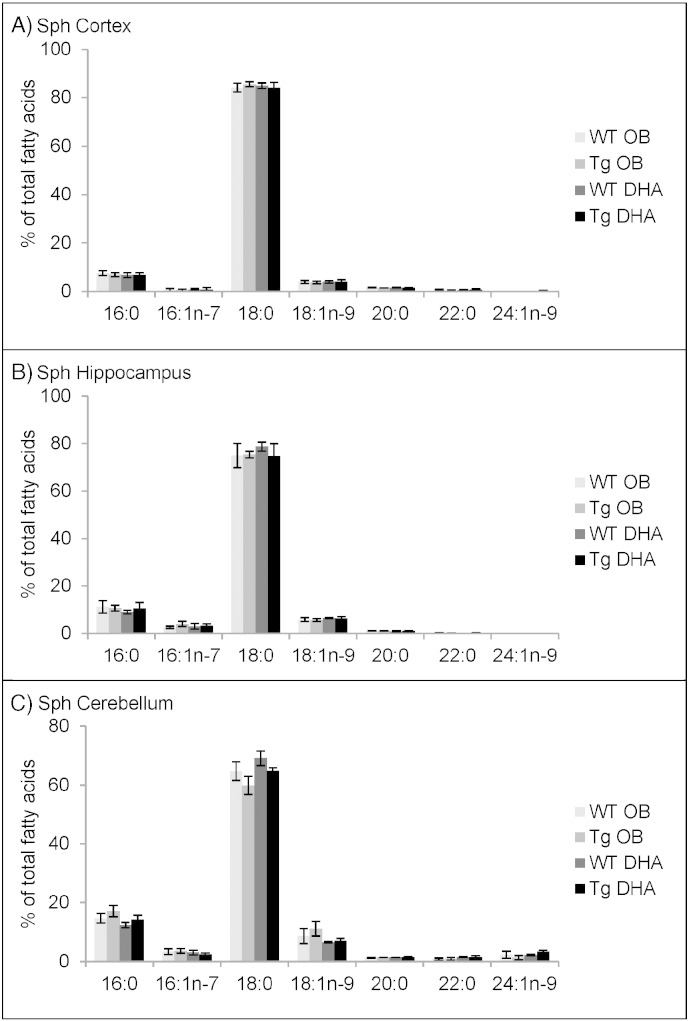
Main fatty acids in sphingomyelin (Sph) from cortex (A), hippocampus (B) and cerebellum (C) of 16 month-old wild-type (WT) and transgenic (Tg) mice on the oil blend diet (OB) or on the DHA diet. Results are represented as mean percentages of total fatty acids (Tg OB (n = 3), WT OB (n = 3), Tg DHA (n = 3), WT DHA (n = 3)) ± SEM. Analysis by GLC. The amounts of Sph were, on average, 1.73 ± 0.14, 1.65 ± 0.11 and 1.14 ± 0.06 μg fatty acids/mg fresh weight for the cortex, hippocampus and cerebellum, respectively. There were no significant differences in the lipid amounts with diet or genotype.

**Fig. 6:**
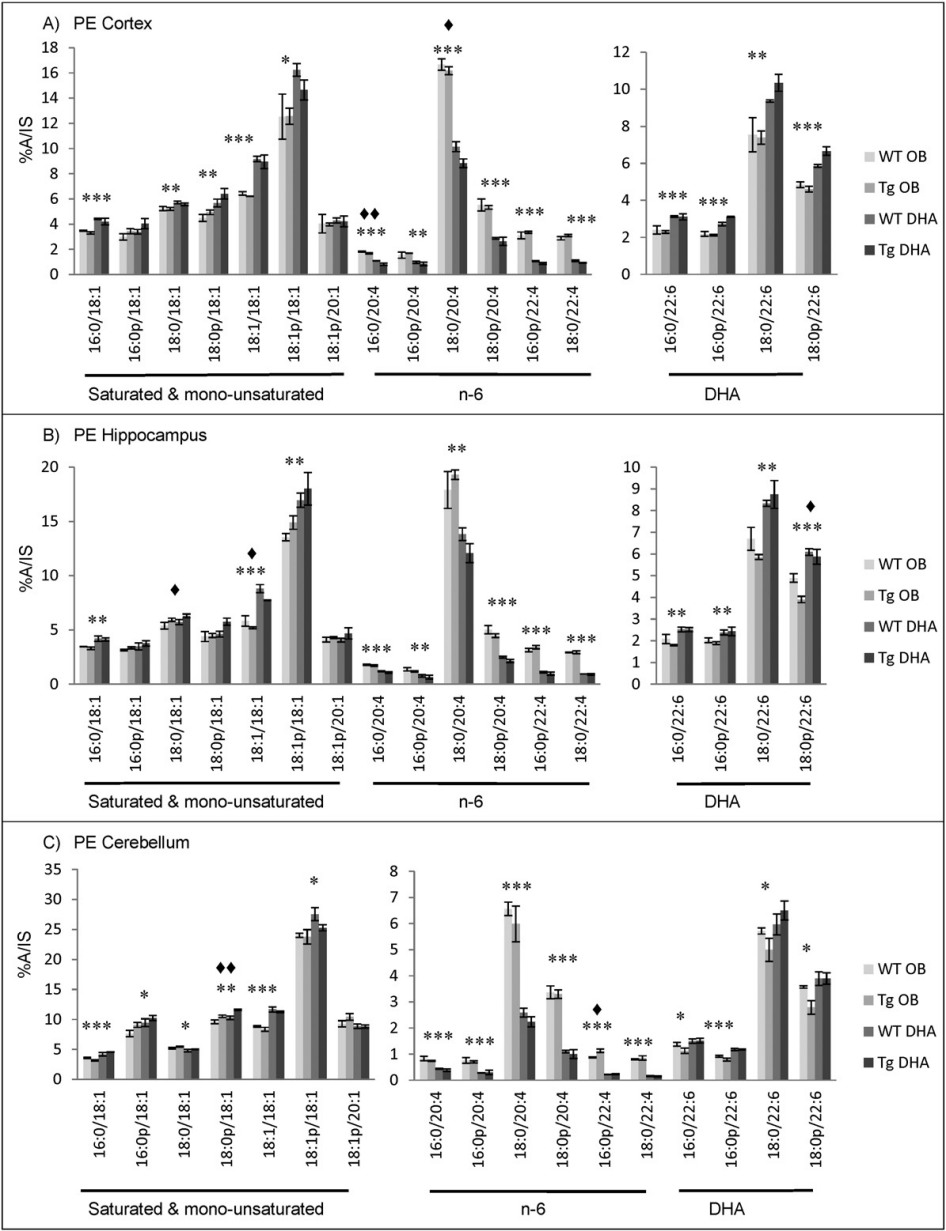
Phosphatidylethanolamine (PE) main molecular species from the cortex (A), hippocampus (B) and cerebellum (C) of 16 month-old wild-type (WT) and transgenic (Tg) mice on the oil blend diet (OB) or on the DHA diet. Results are expressed as percentages (%) of the total pool of lipids analysed for that class, using area for analyte (A) divided by the internal standard (IS), for each molecular species (Tg OB (n = 3), WT OB (n = 3), Tg DHA (n = 3), WT DHA (n = 3)) ± SEM. Analysis by LC–MS/MS. p, plasmenyl (or plasmalogen); significant effect of diet, *p < 0.05, **p < 0.01, ***p < 0.001; significant effect of genotype, ♦ p < 0.05, ♦♦ p < 0.01.

**Fig. 7:**
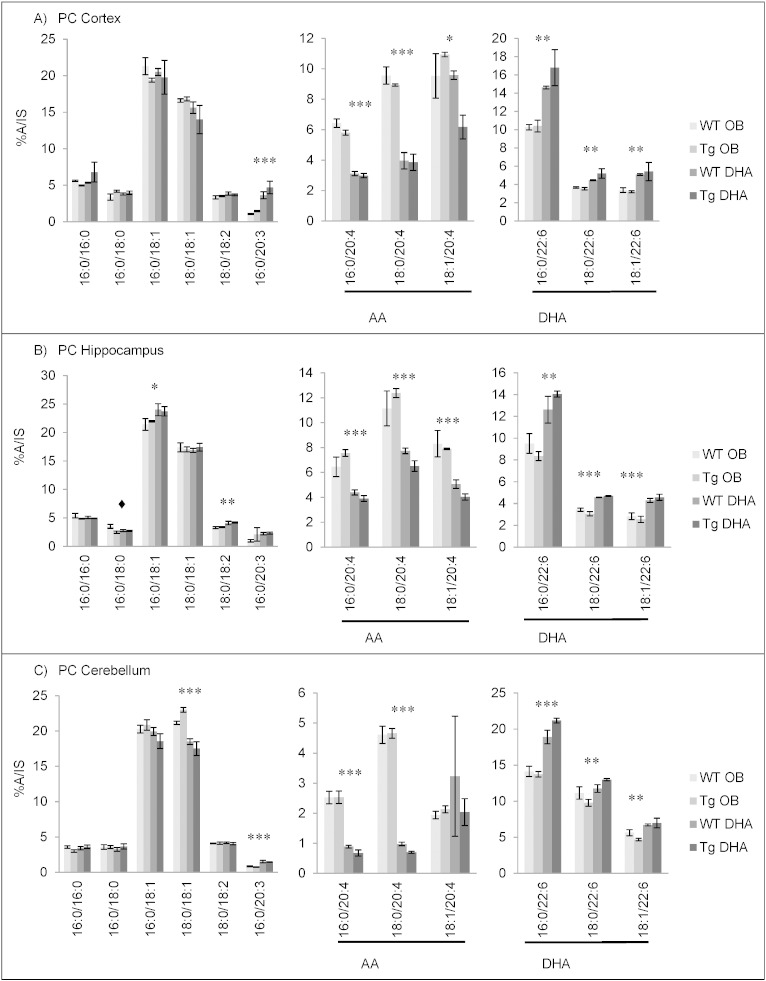
Phosphatidylcholine (PC) main molecular species from the cortex (A), hippocampus (B) and cerebellum (C) of 16 month-old wild-type (WT) and transgenic (Tg) mice on the oil blend diet (OB) or on the DHA diet. Results are expressed as percentages (%) of the total pool of lipids analysed for that class, using area for analyte (A) divided by the internal standard (IS), for each molecular species (Tg OB (n = 3), WT OB (n = 3), Tg DHA (n = 3), WT DHA (n = 3)) ± SEM. Analysis by LC–MS/MS. Significant effect of diet, *p < 0.05, **p < 0.01, ***p < 0.001; significant effect of genotype, ♦ p < 0.05.

**Fig. 8:**
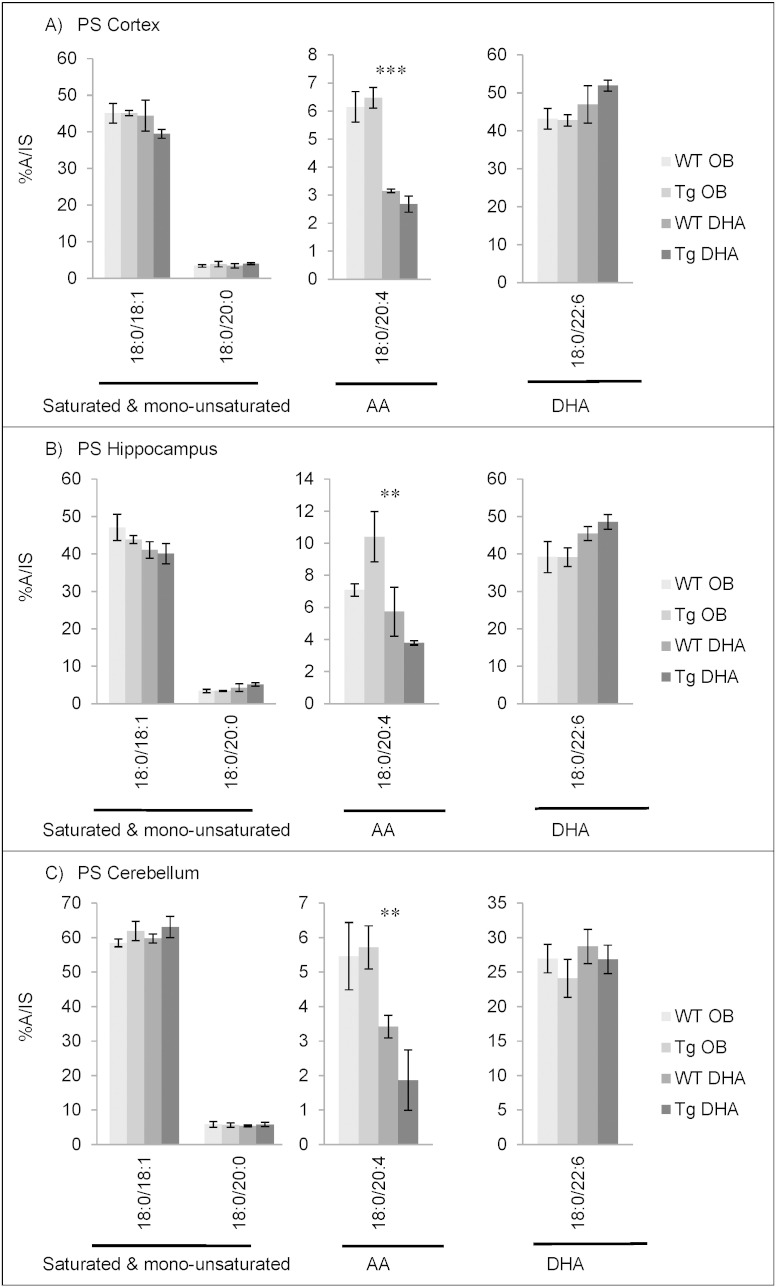
Phosphatidylserine (PS) main molecular species from the cortex (A), hippocampus (B) and cerebellum (C) of 16 month-old wild-type (WT) and transgenic (Tg) mice on the oil blend diet (OB) or on the DHA diet. Results are expressed as percentages (%) of the total pool of lipids analysed for that class, using area for analyte (A) divided by the internal standard (IS), for each molecular species (Tg OB (n = 3), WT OB (n = 3), Tg DHA (n = 3), WT DHA (n = 3)) ± SEM. Analysis by LC–MS/MS. Significant effect of diet, **p < 0.01, ***p < 0.001.

**Fig. 9:**
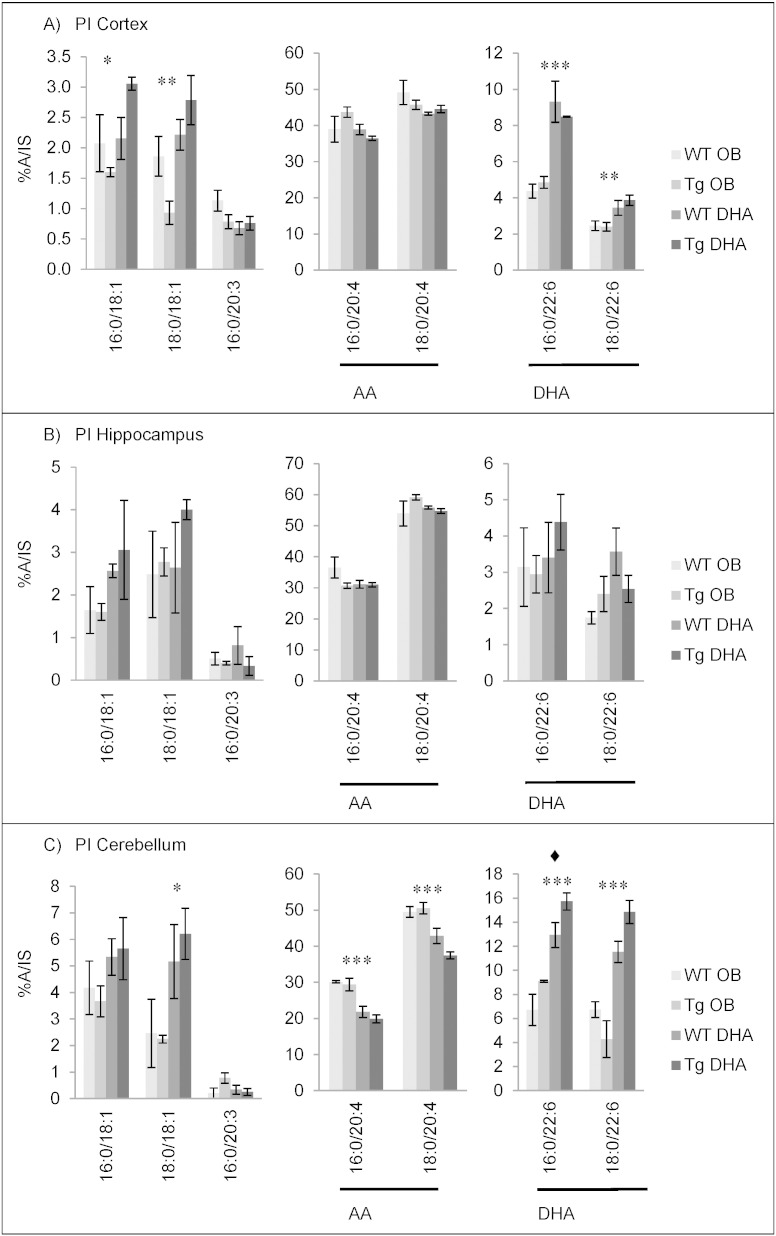
Phosphatidylinositol (PI) main molecular species from the cortex (A), hippocampus (B) and cerebellum (C) of 16 month-old wild-type (WT) and transgenic (Tg) mice on the oil blend diet (OB) or on the DHA diet. Results are expressed as percentages (%) of the total pool of lipids analysed for that class, using area for analyte (A) divided by the internal standard (IS), for each molecular species (Tg OB (n = 3), WT OB (n = 3), Tg DHA (n = 3), WT DHA (n = 3)) ± SEM. Analysis by LC–MS/MS. Significant effect of diet, *p < 0.05, **p < 0.01, ***p < 0.001; significant effect of genotype, ♦ p < 0.05.

To perform PL analysis, individual phosphoglyceride species from murine brain lipid extracts were separated by RP-HPLC on the basis of their acyl chain length on a Luna 3-μm C18 (2) 150 × 2 mm column (Phenomenex Ltd., Macclesfield, UK) with a gradient of 50–100% solvent B over 10 min, followed by 30 min at 100% solvent B, where A = methanol:acetonitrile:water, 60:20:20 (v/v) with 1 mM ammonium acetate and B = 1 mM methanolic ammonium acetate at a flow rate of 200 μl/min and analysed in MRM scan mode as described above, at a scan rate of 100 ms. Data was acquired and individual peak areas integrated using Analyst 1.4.1 (Applied Biosystems). Each phospholipid species was expressed as a ratio of the peak area relative to the di-14:0 internal standard of the same lipid class. Di-14:0-PI was not available commercially so di-14:0-PE was used for the PI normalisation. For each phospholipid class, data are reported as percentages of the total signal. The main species are presented in Fig. 6, Fig. 7, Fig. 8, Fig. 9, but all species analysed are presented in Supplementary Tables 31–45. We recognise that mass spectrometry is not strictly quantitative with, for example, varying ionisation values for individual lipid classes and for different fatty acyl chain lengths. However, here we are comparing lipids from two dietary regimes or two animal lines always under the same conditions. The signals can be compared to the fresh weights by utilising the results provided by the quantitative GLC data as stated in the legends to Fig. 1, Fig. 2, Fig. 3, Fig. 4, Fig. 5.

In the results showing the molecular species, the letter “p” indicates plasmalogen (plasmenyl).

### 2.8 Statistical analyses

All statistical analyses using univariate ANOVA with genotype and diet as factors were performed using SPSS 12.01 (SPSS Inc., USA). p < 0.05 was considered statistically significant.

Additional analyses were carried out using the non-parametric Friedman test (SPSS Inc., USA). Significant X^2^ values were evaluated using post-hoc Wilcoxon signed-rank tests. The two analyses complimented each other in terms of the main significant differences.

## 3 Results

### 3.1 Accumulation of β-amyloid in cortex and hippocampus of transgenic mice

As can be seen in Table 2, the Tg2576 mice accumulated both 1–40 and 1–42 β-amyloid with age. From 12 to 21 months of age, statistical analysis confirmed that there were increasing levels of amyloid detected with more of the 1–40 fragment and greater levels of insoluble material, in general. Nevertheless, despite numerical decreases, the impact of DHA diet on amyloid failed to reach statistical significance.

### 3.2 Fatty acid analysis of individual phospholipids

Following lipid class separations, we analysed the total fatty acid composition of individual PLs from cortex, hippocampus and cerebellum of 12 and 16 month-old WT and Tg mice. The data for 16 month-old mice main fatty acids are given in Fig. 1, Fig. 2, Fig. 3, Fig. 4, Fig. 5. Full results for 12 and 16 month-old mice are in Supplementary Tables 1–30.

#### 3.2.1 Fatty acid contents of ethanolamine phosphoglycerides

The main fatty acids identified in PE (and its ether derivatives) from cortex, hippocampus and cerebellum of 16 month-old WT and Tg mice on the oil-blend diet or on the DHA diet were 18:0, 18:1n−9, 20:4n−6 (AA) and DHA (Fig. 1). Significant amounts of 16:0, 18:1n−7, 20:1n−9 and 22:4n−6 were also found. 18:1n−9 was more prevalent in cerebellum. DHA was a major fatty acid with 25% to 34% of total fatty acids in cortex, 22% to 32% of total fatty acids in hippocampus and 19% to 27% of total fatty acids in cerebellum. Percentages of DHA were significantly higher from DHA-fed mice and this increase was compensated by a decrease in AA as well as its elongation product 22:4n−6. These simultaneous alterations in DHA and n−6 fatty acids were observed in all three brain areas (Fig. 1). Within the other fatty acids, diet only caused minor alterations (if any) in proportions (Fig. 1, see also Supplementary Tables 1–6).

When comparing the three brain regions, the fatty acid compositions (and their changes with diet) were similar for ethanolamine phosphoglycerides in the cortex and hippocampus. The cerebellum had lower percentages of 18:0, AA, 22:4n−6 and DHA than the two other brain regions. These decreases were mainly compensated by larger percentages of 18:1n−9 and 20:1n−9.

Comparison of the composition of ethanolamine phosphoglycerides from cortex, hippocampus and cerebellum between 12 months and 16 months of age suggests an age-related decrease of the proportion of very long chain PUFA such as AA, 22:4n−6 and DHA and conversely, an increased proportion of saturated fatty acids such as 16:0 and 18:0 (Supplementary Tables 1–6). This was especially found in cortex and hippocampus. The main DHA decrease was observed in cortex with a reduction of about 5% of total fatty acids from 12 to 16 months. At 12 months, DHA diet increased the DHA content by about 7% of total fatty acids regardless of the genotype. Interestingly, although DHA feeding reduced the proportion of AA, the latter's metabolic precursor 20:3n−6 was increased in PE and other lipids (see Supplementary Tables). This may be due to reduced feedback from the lowered AA in the synthetic metabolic pathway.

Although the effect was not statistically significant, it is interesting to note that, at 16 months of age, the mean percentages of DHA in oil-blend fed mice were constantly higher in WT mice than Tg mice and this was reversed in the DHA-fed mice. At this age, the DHA diet increased DHA levels by about 9% in Tg mice while it was only increased by about 5% total fatty acids in WT mice. Thus, DHA supplementation had a greater enhancing effect on brain DHA levels in Tg than WT mice while WT mice on oil-blend diet maintained a higher percentage of DHA than Tg mice on the same diet.

#### 3.2.2 Fatty acid contents of choline phosphoglycerides

The main fatty acid in PC (and its ether derivatives) was 16:0 (Fig. 2). 18:0, 18:1n−9, 18:1n−7, AA and DHA were also present in significant amounts. Compared to ethanolamine phosphoglycerides, the percentage of palmitic acid (16:0), in particular, was elevated in all three brain areas studied. AA was found at around a third of its percentage in PE (Fig. 1) while DHA was much lower, especially in cortex and hippocampus with less than 5% and less than 3% of total fatty acids, respectively. Diet changed DHA percentages by around a third and those of AA by about half. As with PE, reciprocal changes in these PUFA were found but 22:4n−6 was less than 1% in PC and, therefore, not shown in Fig. 2. Where significant changes in stearic and oleic acids were caused by diet, feeding the DHA-enriched diet lowered stearic acid and raised oleic acid, as seen in PE.

Comparison of choline phosphoglycerides from the three brain areas showed that the main fatty acid percentages were rather similar. However, the percentage of AA was lowest in cerebellum while DHA was highest there.

An age-related decrease of the proportion of AA and DHA was seen in the three brain areas (Supplementary Tables 7–12). As previously noted for PE, this was greater in cortex and hippocampus.

As also observed with PE, Tg mice seemed more sensitive to diet, with the maximum DHA percentages in all three regions obtained with Tg mice on DHA diet.

#### 3.2.3 Fatty acid contents of phosphatidylserine

PS was dominated by a high percentage of stearic acid (18:0) (Fig. 3). In cortex and hippocampus, DHA was the next most abundant fatty acid while in cerebellum, oleic acid (18:1) (at about 30%) was double that of DHA. AA and its elongation product, 22:4n−6 were relatively minor components (2% or less) and were halved by feeding a DHA-diet. Interestingly, in contrast to PE (Fig. 1), DHA feeding had little or no significant effect on the percentage of DHA in PS (Fig. 3).

Comparison of the composition of PS from cortex, hippocampus and cerebellum between 12 months and 16 months of age suggests an age-related decrease of the proportion of DHA and a conversely increased proportion of saturated 18:0. From 12 to 16 months, DHA levels decreased by about 5% total fatty acids, on average (Supplementary Tables 13–18).

#### 3.2.4 Fatty acid contents of phosphatidylinositol

PI contained the highest percentage of AA of all the phospholipids examined (Fig. 4). Like PS (Fig. 3), it also contained up to about 50% 18:0. These two fatty acids accounted for 60–75% of the total. DHA was relatively minor, especially in the hippocampus. It was highest in the cerebellum and its percentage was approximately doubled with DHA diet. Interestingly, eicosapentaenoic acid (20:5n−3, EPA) was detected in the PI of all brain areas after DHA feeding (Fig. 4). This contrasted with other phosphoglycerides where the proportion of EPA was no higher than 0.5% (Fig. 1, Fig. 2, Fig. 3). Since EPA was not present in the diet, this suggests a modification of metabolism possibly including retroconversion of DHA to EPA [44] which has been specifically demonstrated in both humans and rats by the use of [^13^C]DHA [45]. The appearance of EPA in PI only suggests that enzymes involved in its formation may be selective for PUFA with a Δ5 *cis* double bond (like EPA and AA).

Comparison of the composition of PI from cortex, hippocampus and cerebellum between 12 months and 16 months of age again suggests an age-related decrease of the proportion of PUFA and conversely increased proportions of 16:0 and 18:0. From 12 to 16 months, DHA level decreased by about 1% total fatty acids, on average. There was little effect of genotype (Fig. 4, Supplementary Tables 19–24).

#### 3.2.5 Fatty acid contents of sphingomyelin

The other major phospholipid was sphingomyelin (Sph). Although Sph contained a variety of fatty acids, including nervonic acid (24:1n−9), its composition was heavily dominated by 18:0 (Fig. 5). This represented up to about 85% of the total fatty acids in cortex and was lowest in cerebellum (60–70%). There were no effects of diet on the fatty acid composition in any of the three brain areas. As others have noted [2], [3], [46], [47], [48], [49], Sph contained virtually no AA or DHA.

Comparing the data from 12- and 16-month old mice (Supplementary Tables 25–30) suggested an age-related decrease in the proportions of 22:0 and 24:1n−9 with reciprocal increases in 16:0 and 18:0.

The above analyses allowed some general conclusions. First, the effects of diet were greater than for genotype. Second, DHA supplementation caused different effects depending on the PL class. Quantitatively, the largest effects of the DHA diet were on PE. Third, an age-dependent decrease in PUFA content of all phosphoglycerides was found. Fourth, dietary supplementation with DHA generally reduced the proportion of AA (and, where present, 22:4n−6) of PE, PC and PS but had negligible effects on other fatty acids of PL. Fifth, Tg mice seemed more sensitive to diet than WT and this was mostly observed in PE and PC.

### 3.3 Analysis of brain phosphoglyceride molecular species composition

Following on from total fatty acid analysis, we undertook a lipidomic approach to determine specific molecular species in brain phosphoglycerides, in order to examine which species predominated and whether changes on DHA supplementation were restricted to particular lipids.

Negative-ion LC–MS/MS analysis of ethanolamine phosphoglycerides demonstrated prominent peaks corresponding to the main molecular species: 18:1p/18:1, 18:0/20:4, and 18:0/22:6 in cortex and hippocampus and, in addition, 16:0p/18:1, 18:0p/18:1, 18:1/18:1 and 18:1p/20:1 in cerebellum (Fig. 6, Supplementary Tables 31–36). Feeding a DHA-enriched diet increased all major DHA-containing species significantly, the effects being most notable in cortex and hippocampus, and particularly in Tg mice (Fig. 6), as observed with GLC analysis (Fig. 1). In all three brain areas, the main DHA-containing species were those with 18:0 or its plasmanyl-derivative (18:0p/22:6). As expected, the DHA diet resulted in significant decreases in all AA and 22:4-containing molecular species. With mice on oil-blend diet, the main molecular species in cortex and hippocampus was 18:0/20:4. For PE species which did not contain PUFA, DHA feeding resulted in a significant increase in the dioleoyl (18:1) species (Fig. 6). The same overall patterns of change with diet were seen for 12 month-old and 16 month-old mice (Supplementary Tables 31–36). These included alterations in both PE and ethanolamine plasmalogens (Supplementary Tables 31–36). Genotypic alterations were found in both ethanolamine phosphoglyceride classes and in all three brain areas (Supplementary Tables 31–36).

The main molecular species of PC, as revealed by LC–MS/MS, are shown in Fig. 7. With the oil blend diet, the main species in the three brain regions were 16:0/18:1, 18:0/18:1, 16:0/20:4, 18:0/20:4, 18:1/20:4 and 16:0/22:6. The molecular species distribution of PC was very similar for cortex and hippocampus while cerebellum showed somewhat greater amounts of 18:0/22:6 species while AA-containing species were not as prevalent (Fig. 7), which was also seen with GLC analysis (Fig. 2). Feeding DHA caused an increase in all DHA-containing molecular species of PC while the AA-containing species were approximately halved. This effect was particularly pronounced in Tg mice, as seen with PE and with GLC analysis of PC (Fig. 2). There were few significant changes in other molecular species although one containing the Mead acid (Δ5, 8, 11-eicosatrienoic acid, 16:0/20:3) was increased rather consistently on DHA feeding (Fig. 7, Supplementary Tables 37–39). With age, there seemed to be a number of changes to the molecular species distribution in PC. For the cortex and hippocampus, 12 month-old mice on the oil blend diet had increased 16:0/16:0 and 16:0/18:1 and lower levels of 18:0/18:1, 18:0/20:4, 18:1/20:4 and all species containing DHA compared to 16 month-old mice. The same differences were found for the cerebellum except that 18:1/20:4 was hardly altered (Supplementary Tables 37–39).

Given the rather simple composition of PS major fatty acids (Fig. 3), it was not surprising that the molecular species analysis was dominated by 18:0-containing species (Fig. 8, Supplementary Tables 40–42). In agreement with the overall fatty acid composition (Fig. 3), the main molecular species were 18:0/18:1 and 18:0/22:6 (Fig. 8). Again, as reflected in the total fatty acid changes with diet (Fig. 3), the DHA diet caused an approximate halving of the 18:0/20:4 species but caused no significant increase in the 18:0/22:6 species (Fig. 8). In contrast to the other major DHA-containing PL, PE, there were negligible changes caused to PS molecular species by genotype (Supplementary Tables 40–42).

The main molecular species of phosphatidylinositol (PI) for 16 month-old mice are shown in Fig. 9. As noted before (Fig. 4), the DHA diet caused little change to AA in PI. Only in the cerebellum were there significant decreases in the AA-containing molecular species. This contrasted clearly from other phosphoglycerides (Fig. 1, Fig. 2, Fig. 3, Fig. 6, Fig. 7, Fig. 8). Other significant alterations to the cerebellum PI, included increases in 18:0/18:1, 16:0/22:6 and 18:0/22:6 (Fig. 9). Such changes were also found in other brain fractions and at 12 months age (Supplementary Tables 43–45). As with PS, genotype caused negligible changes to the molecular species composition of PI (Supplementary Tables 43–45).

## 4 Discussion

It is well known that β-amyloid accumulates in the brain of Tg2576 mice from about 8–9 months of age. For the ages used (12 and 16 months), as well as 21 months, we monitored the levels of β-amyloid in the cerebellum, cortex and hippocampus of our Tg2576 mice and observed a significant accumulation of the protein in the cortex and hippocampus (Table 2) but not in the cerebellum (data not shown). This agreed with previous data [50] as was the progressive increase in β-amyloid levels with age [50], [51]. The higher levels of insoluble *versus* soluble fraction and the greater accumulation of the 1–40 fragment were also consistent with published results [50], [51]. In general, the DHA-enriched diet gave lower levels of β-amyloid accumulation (Table 2), as seen before [10], [32], [34], [37].

Given the common usage of the Tg2576 amyloid mouse model [38], [52] and the widespread evidence for the connection between brain lipids and AD, it is surprising that a detailed study of brain lipids in this strain has not been undertaken before.

Our analyses of lipid classes and their molecular species give data which is consistent with reported data for normal mice [11], [53], [54] and, in particular, the results of Axelsen and Murphy [55]. The latter showed that molecular species of phosphoglycerides had a fairly similar distribution in hippocampus and cortex, as we have. However, here we show that cerebellum lipids were somewhat different (Fig. 1, Fig. 2, Fig. 3, Fig. 4, Fig. 5, Fig. 6, Fig. 7, Fig. 8, Fig. 9) as suggested in studies of other animals or humans (see [53], [56]). This may reflect the different functions of these brain regions where cortex and hippocampus are involved in memory whereas the cerebellum is associated with fine grain motor control [57], [58], [59].

In animal brains, PE and PC are the main phospholipids [60], [61] with plasmalogen derivatives representing up to half of the PE class [2]. Our data are in agreement, as is the observation that PI is enriched in AA while PE and PS are the main DHA-containing phospholipids. However, it is noticeable that, on DHA supplementation, the main increase in the PL content of DHA is concentrated in ethanolamine phosphoglycerides (Fig. 1, Fig. 6, Supplementary Tables 1–6 and 31–36) whereas the composition of PS is hardly changed (Fig. 3, Fig. 8, Supplementary Tables 13–18 and 40–42). Together with the preservation of selective acyl compositions within PL classes, this points to stringent metabolic controls (see [62], [63]) which merit further investigation.

DHA supplementation also caused a reciprocal significant decrease in the proportion of AA and, usually, 22:4n−6 in the different phosphoglycerides even when the proportion of DHA did not show a significant rise (as in PS, Fig. 3). These reciprocal changes in DHA and AA on feeding a DHA-enriched diet are in agreement with results from previous studies described earlier where transgenic mice expressing AD pathologies were fed DHA-enriched diets [10], [33], [34], [36], [64]. Not only do these two fatty acids compete during phospholipid synthesis partly due to the fact that the precursor fatty acids ALA and linoleic acid (LA) are metabolised by the same sequence of desaturases and elongases [65], [66], [67], but they are also precursors of bioactive mediators with potentially opposite effects. Thus, while n−6 derived mediators such as PGE_2_ and leukotriene B_4_ have pro-inflammatory properties, the n−3 derived molecules are non- or anti-inflammatory [67]. Furthermore, n−3 derived resolvins, docosatrienes and neuroprotectins are all anti-inflammatory and are suggested to have important functions in resolving inflammation and in protecting neurones [68], [69]. This may reduce local inflammation caused by the amyloid plaque formation in AD brain and subsequently reduce the severity of the symptoms. This provides an important reason for the proposition that adequate dietary n−3 PUFA are needed for good health, including reducing the prevalence of AD. Several large-scale epidemiological studies have shown benefit of dietary fish oil or n−3 PUFA in reducing the risks of developing AD [15], [16], [17]. This, in turn, has led to intervention trials with fish oils or n−3 PUFA to alleviate AD symptoms. Not surprisingly such trials (in patients with clear signs of neuronal damage, loss and dementia) have not proven generally successful [70]. However, it is relevant to note the possible positive effects of therapies with DHA-derivatives [71]. Moreover, there have been benefits of dietary DHA reported for cases of early cognitive impairment [72], [73], [74], [75], [76]. This was also shown in animal studies both with normal mice [28] or AD models [35], [36], [37], [76].

So far as age is concerned, we found a general decrease in the PUFA contents of phosphoglycerides in 16 month-old mice compared to 12 month-old animals. This was found in all three parts of the brain analysed and for all four phosphoglycerides. Our results are consistent with cortex data for PE which was specifically analysed by Han et al. [11].

We showed that the Tg2576 mouse reacts to supplementary dietary DHA in a broadly similar manner to wild-type animals. However, subtle changes were found in the molecular species of phosphoglyceride classes of the Tg2576 mice compared to wild type. PE (and its plasmalogens) appeared to show more changes than other PL classes. For example, in 16 month-old mice, there were ten significant differences in ethanolamine phosphoglycerides but only three in all the other phosphoglycerides combined (Supplementary Tables 32, 34, 36–42). Furthermore, significantly changed ethanolamine lipids included four plasmalogen species which contrasted to results reported for rats [77]. However, our data bear comparison with analysis of brains from Alzheimer's patients [7], [8], [11]. Thus, while total brain concentration of different phosphoglycerides are little changed in AD by comparison with control brains [13], [14], there are reported changes for their fatty acid compositions especially for PE [13]. Moreover, the plasmalogen species of PE were notably decreased [11], [12], [14], [78] and for the cortex this was found to correlate with clinical dementia ratings [11]. In addition to these changes, lower percentages of DHA were generally observed in Tg mice on the standard diet, compared to WT mice on the same diet and, in contrast, larger percentage increases in DHA were seen more in Tg mice on DHA diet than WT mice on the same diet. This was mainly observed in PE and PC and some of their specific DHA-containing molecular species. Although these observations might point to a deregulation of lipid metabolism affecting DHA accumulation in the brain of Tg mice, further studies will be needed to clarify the question of a connection between AD and low brain DHA levels (particularly in PE).

## 5 Conclusions

In conclusion, we demonstrate the selective incorporation of dietary DHA into different PL with PE being favoured quantitatively. Furthermore, the significant changes in both diacyl- and PE plasmalogens in the amyloid (Tg2576) mouse model suggest a connection between PE and behaviour, as proposed for AD patients. Further research should extend these experiments as well as including certain sphingolipids that are also altered in AD [14], [45], [79], [80]. The latest advances in mass spectrometry [81], [82] and similar techniques [83], [84] offer much promise in defining detailed lipid distributions within different brain areas. By such means, we will gain more information that should help explain the role of PE in relation to dementia (see [55]) and the potential protective effects of dietary DHA in preserving cognition. Our comprehensive data with the amyloid Tg2576 model mouse add significantly to the body of evidence linking DHA-supplemented diets in mammals to specific changes in brain lipid content and provide a basis for further research as suggested above.

## Conflict of interest

We confirm that there is no conflict of interest involved in this study.

## Transparency document

Transparency document

## Figures and Tables

**Table 1:** Fatty acid composition of the oil blend and the DHA diets.

Fatty acids	Oil blend diet: standard rodent feed + 5% oil blend	DHA diet: standard rodent feed + 5% DHA rich oil
12:0	0.05 ± 0.01	0.04 ± 0.01
14:0	0.08 ± tr.	0.38 ± tr.
16:0	1.65 ± 0.07	0.86 ± tr.
16:1	0.03 ± tr.	0.08 ± tr.
18:0	0.37 ± 0.01	0.07 ± tr.
18:1n−9	2.87 ± 0.13	1.66 ± 0.01
18:2n−6	2.06 ± 0.09	1.60 ± 0.02
18:3n−3	0.20 ± 0.01	0.17 ± 0.01
20:0	0.01 ± tr.	0.01 ± tr.
20:1	0.02 ± tr.	0.01 ± tr.
20:2	0.01 ± tr.	N.D.
20:4n−6	N.D.	N.D.
20:3n−3	N.D.	N.D.
20:5n−3	N.D.	N.D.
22:0	0.01 ± tr.	0.01 ± tr.
22:5n−3	N.D.	0.01 ± tr.
22:6n−3	N.D.	1.86 ± 0.04
24:0	N.D.	N.D.
Total SAT	2.17 ± 0.08	1.37 ± 0.01
Total MUFA	2.93 ± 0.13	1.75 ± 0.01
Total PUFA	2.27 ± 0.10	3.64 ± 0.05
Total n−3	0.20 ± 0.01	2.04 ± 0.03
Total n−6	2.06 ± 0.09	1.60 ± 0.02
n−6/n−3 ratio	10.3	0.8

Results are represented as mean percentage of fresh diet (by weight) ± SEM (n = 3). N.D., not detected; tr., trace (less than 0.005); SAT, saturated fatty acids; MUFA, monounsaturated fatty acids; PUFA, polyunsaturated fatty acids. Analysis was carried out as detailed in Section 2.6.

**Table 2:** Levels of amyloid-β in Tg2576 mice.

	Amyloid-β in pg/μg protein			
1–40		1–42	
Soluble	Insoluble	Soluble	Insoluble
12 month-old				
Cortex	4.5 ± 3.6 (44%)	48.0 ± 21.9 (84%)	n.d.	6.6 ± 2.7 (86%)
Hippocampus	18.9 ± 9.4 (46%)	16.0 ± 7.6 (37%)	1.0 ± 0.5 (48%)	1.1 ± 0.5 (47%)

16 month-old				
Cortex	31.2 ± 18.1 (96%)	223.6 ± 73.1 (65%)	2.3 ± 1.7 (123%)	15.4 ± 4.1 (80%)

21 month-old				
Cortex	31.8 ± 7.8 (107%)	284.3 ± 90.2 (88%)	3.5 ± 0.8 (92%)	25.2 ± 5.4 (83%)
Hippocampus	128.8 ± 60.1 (84%)	227.9 ± 134.3 (73%)	11.7 ± 3.6 (92%)	42.6 ± 17.8 (69%)

n.d., none detected. Figures in brackets show data for mice on the DHA-enriched diet as a percentage of the data for mice on the oil blend diet. Data show means ± SEM (n = 6 for 12 month-old, n = 3 for 16 and 21 month-old mice). Overall main effect of age p < 0.001 on amyloid levels. Despite numerical decreases, the impact of DHA did not reach conventional levels of significance.

## Appendix A Supplementary data

Supplementary tables Image 1
