# Supplementary material for: Dietary DHA supplementation causes selective changes in phospholipids from different brain regions in both wild type mice and the Tg2576 mouse model of Alzheimer's disease
Source: Biochim Biophys Acta. 2016 Jun;1861(6):524–37. doi: 10.1016/j.bbalip.2016.03.005 (PMC4847476; doi:10.1016/j.bbalip.2016.03.005)
Supplement: Supplementary file 2 — Supplementary tables [file mmc1.docx]

AD paper supplementary tables

**Tab1e 1**. Fatty acid composition of phosphatidylethanolamine (PE) from cortex of 12 month-old wild-type (WT) and transgenic (Tg) mice on the oil blend diet (OB) or the DHA diet. Results are represented as mean percentage of total fatty acids ± SEM. Analysis by GLC.

|  | PE Cortex - 12 months | | | | | |
| --- | --- | --- | --- | --- | --- | --- |
| Fatty acid | | Tg OB (n = 4) | WT OB (n = 4) | Tg DHA (n = 4) | WT DHA (n = 4) |  |
| 12:0 | | N.D. | N.D. | N.D. | N.D. |  |
| 16:0 | | 4.6 ± 0.2 | 5.4 ± 0.6 | 5.5 ± 0.8 | 5.1 ± 0.3 |  |
| 16:1n-7 | | 0.3 ± 0.1 | 0.4 ± 0.1 | 0.4 ± 0.2 | 0.3 ± 0.1 |  |
| 18:0 | | 22.1 ± 1.0 | 21.7 ± 0.5 | 21.9 ± 0.7 | 21.6 ± 0.5 |  |
| 18:1n-9 *** | | 8.6 ± 0.2 | 8.7 ± 0.2 | 9.6 ± 0.2 | 10.1 ± 0.3 |  |
| 18:1n-7 * | | 1.8 ± 0.1 | 1.8 ± tr. | 1.6 ± tr. | 1.7 ± 0.1 |  |
| 18:2n-6 | | 0.4 ± tr. | 0.3 ± tr. | 0.5 ± 0.1 | 0.4 ± tr. |  |
| 18:3n-6 | | N.D. | N.D. | N.D. | N.D. |  |
| 18:3n-3 | | 0.1 ± tr. | 0.1 ± tr. | 0.1 ± tr. | 0.1 ± tr. |  |
| 20:0 | | 0.1 ± tr. | 0.1 ± tr. | 0.1 ± tr. | 0.1 ± tr. |  |
| 20:1n-9 ^♦^ | | 1.5 ± 0.1 | 1.9 ± 0.1 | 1.5 ± 0.1 | 1.7 ± 0.1 |  |
| 20:3n-6 *** | | 0.4 ± tr. | 0.3 ± tr. | 1.1 ± 0.1 | 0.9 ± tr. |  |
| 20:4n-6 *** | | 13.3 ± 0.4 | 13.0 ± 0.4 | 7.7 ± 0.2 | 7.4 ± 0.2 |  |
| 20:3n-3 | | 0.1 ± 0.1 | N.D. | 0.1 ± tr. | N.D. |  |
| 20:5n-3 *** | | N.D. | N.D. | 0.5 ± tr. | 0.5 ± tr. |  |
| 22:0 | | N.D. | N.D. | N.D. | N.D. |  |
| 22:1n-9 | | N.D. | 0.1 ± tr. | N.D. | N.D. |  |
| 22:4n-6 *** | | 5.6 ± 0.2 | 5.6 ± 0.3 | 2.0 ± tr. | 1.9 ± 0.1 |  |
| 22:5n-3 *** | | 0.2 ± tr. | 0.2 ± tr. | 0.8 ± tr. | 0.8 ± tr. |  |
| 22:6n-3 *** | | 30.1 ± 0.8 | 30.1 ± 0.6 | 37.9 ± 0.7 | 37.1 ± 0.6 |  |
| 24:0 | | N.D. | N.D. | N.D. | N.D. |  |
| 24:1n-9 | | N.D. | N.D. | N.D. | N.D. |  |
| other minor FA | | 10.8 ± 1.2 | 10.2 ± 1.9 | 8.6 ± 1.9 | 10.4 ± 0.7 |  |
|  | |  |  |  |  |  |
| Total SAT | | 26.9 ± 1.0 | 27.3 ± 0.7 | 27.5 ± 1.3 | 26.8 ± 0.2 |  |
| Total MUFA * | | 12.2 ± 0.2 | 12.9 ± 0.4 | 13.2 ± 0.1 | 13.8 ± 0.5 |  |
| Total PUFA | | 50.1 ± 0.7 | 49.6 ± 1.0 | 50.6 ± 0.7 | 49.0 ± 0.3 |  |
| Total n-3 FA *** | | 30.5 ± 0.8 | 30.4 ± 0.6 | 39.4 ± 0.8 | 38.4 ± 0.6 |  |
| Total n-6 FA *** | | 19.6 ± 0.4 | 19.3 ± 0.7 | 11.3 ± 0.2 | 10.6 ± 0.3 |  |

SFA, saturated fatty acids; MUFA, monounsaturated fatty acids; PUFA, polyunsaturated fatty acids; N.D., not detected; tr., trace (less than 0.05); significant effect of diet, * p < 0.05, *** p < 0.001; significant effect of genotype, ^♦^ p < 0.05. For fatty acid abbreviations see Table 1.

**Tab1e 2.** Fatty acid composition of phosphatidylethanolamine (PE) from cortex of 16 month-old wild-type (WT) and transgenic (Tg) mice on the oil blend diet (OB) or the DHA diet. Results are represented as mean percentage of total fatty acids ± SEM. Analysis by GLC.

|  | PE Cortex - 16 months | | | | | |
| --- | --- | --- | --- | --- | --- | --- |
| Fatty acid | | Tg OB (n = 3) | WT OB (n = 3) | Tg DHA (n = 3) | WT DHA (n = 3) |  |
| 12:0 | | N.D. | N.D. | N.D. | N.D. |  |
| 16:0 | | 7.1 ± 0.5 | 7.1 ± 0.5 | 7.0 ± 0.5 | 7.6 ± 0.7 |  |
| 16:1n-7 | | 0.3 ± tr. | 0.3 ± 0.1 | 0.3 ± tr. | 0.4 ± 0.1 |  |
| 18:0 ** | | 27.8 ± 0.3 | 26.9 ± 0.3 | 25.7 ± 0.4 | 26.3 ± 0.2 |  |
| 18:1n-9 ** | | 9.6 ± 0.3 | 9.2 ± 0.3 | 10.6 ± 0.4 | 11.0 ± 0.2 |  |
| 18:1n-7 | | 2.2 ± 0.2 | 2.0 ± 0.2 | 1.7 ± 0.4 | 1.9 ± 0.2 |  |
| 18:2n-6 | | 0.2 ± tr. | 0.2 ± tr. | 0.3 ± tr. | 0.3 ± tr. |  |
| 18:3n-6 | | N.D. | N.D. | N.D. | N.D. |  |
| 18:3n-3 | | N.D. | N.D. | 0.1 ± tr. | N.D. |  |
| 20:0 | | 0.1 ± tr. | 0.1 ± tr. | 0.1 ± tr. | 0.1 ± tr. |  |
| 20:1n-9 | | 0.8 ± 0.4 | 0.8 ± 0.4 | 0.7 ± 0.4 | 0.8 ± 0.4 |  |
| 20:3n-6 *** | | 0.2 ± tr. | 0.2 ± tr. | 0.7 ± tr. | 0.6 ± tr. |  |
| 20:4n-6 *** | | 12.1 ± 0.1 | 11.9 ± 0.5 | 5.1 ± 0.3 | 6.1 ± 0.2 |  |
| 20:3n-3 | | N.D. | N.D. | 0.1 ± tr. | N.D. |  |
| 20:5n-3 *** | | N.D. | N.D. | 0.4 ± tr. | 0.3 ± tr. |  |
| 22:0 | | N.D. | N.D. | N.D. | N.D. |  |
| 22:1n-9 | | N.D. | N.D. | N.D. | N.D. |  |
| 22:4n-6 *** | | 4.1 ± 0.1 | 3.8 ± 0.1 | 0.9 ± 0.1 | 1.2 ± 0.1 |  |
| 22:5n-3 ** | | 0.1 ± tr. | 0.1 ± tr. | 0.3 ± 0.1 | 0.5 ± tr. |  |
| 22:6n-3 *** | | 25.0 ± 1.2 | 26.1 ± 1.0 | 34.1 ± 1.4 | 30.9 ± 0.9 |  |
| 24:0 | | N.D. | N.D. | N.D. | N.D. |  |
| 24:1n-9 | | N.D. | N.D. | N.D. | N.D. |  |
| Other minor FA * | | 10.2 ± 0.4 | 11.2 ± 0.6 | 12.1 ± 0.3 | 12.0 ± 0.4 |  |
|  | |  |  |  |  |  |
| Total SAT | | 34.9 ± 0.6 | 34.1 ± 0.3 | 32.8 ± 0.9 | 34.0 ± 0.9 |  |
| Total MUFA | | 12.9 ± 0.4 | 12.2 ± 0.6 | 13.6 ± 0.8 | 14.2 ± 0.3 |  |
| Total PUFA | | 42.0 ± 1.2 | 42.5 ± 1.4 | 41.9 ± 1.6 | 39.8 ± 1.0 |  |
| Total n-3 FA *** | | 25.2 ± 1.2 | 26.3 ± 1.0 | 34.9 ± 1.4 | 31.7 ± 0.9 |  |
| Total n-6 FA *** | | 16.7 ± 0.2 | 16.2 ± 0.5 | 7.0 ± 0.4 | 8.1 ± 0.3 |  |

SFA, saturated fatty acids; MUFA, monounsaturated fatty acids; PUFA, polyunsaturated fatty acids; N.D., not detected; tr., trace (less than 0.05); significant effect of diet, * p < 0.05, ** p < 0.01, *** p < 0.001.

**Tab1e 3.** Fatty acid composition of phosphatidylethanolamine (PE) from hippocampus of 12 month-old wild-type (WT) and transgenic (Tg) mice on the oil blend diet (OB) or the DHA diet. Results are represented as mean percentage of total fatty acids ± SEM. Analysis by GLC.

|  | PE Hippocampus - 12 months | | | | | |
| --- | --- | --- | --- | --- | --- | --- |
| Fatty acid | | Tg OB (n = 4) | WT OB (n = 4) | Tg DHA (n = 4) | WT DHA (n = 4) |  |
| 12:0 | | N.D. | N.D. | N.D. | N.D. |  |
| 16:0 | | 6.4 ± 0.5 | 7.1 ± 0.3 | 6.5 ± 0.7 | 6.6 ± 0.8 |  |
| 16:1n-7 | | 0.8 ± 0.3 | 0.6 ± 0.1 | 0.8 ± 0.2 | 0.9 ± 0.5 |  |
| 18:0 | | 23.6 ± 0.5 | 22.8 ± 0.6 | 23.1 ± 0.9 | 22.3 ± 0.3 |  |
| 18:1n-9 | | 11.3 ± 0.8 | 11.7 ± 1.5 | 13.7 ± 0.9 | 13.2 ± 1.0 |  |
| 18:1n-7 | | 1.7 ± 0.2 | 2.1 ± 0.1 | 1.9 ± 0.1 | 1.9 ± 0.1 |  |
| 18:2n-6 ** ^♦^ | | 0.3 ± tr. | 0.2 ± tr. | 0.4 ± tr. | 0.3 ± tr. |  |
| 18:3n-6 | | N.D. | N.D. | N.D. | N.D. |  |
| 18:3n-3 | | 0.1 ± tr. | 0.1 ± tr. | 0.1 ± tr. | 0.1 ± tr. |  |
| 20:0 | | 0.1 ± tr. | 0.1 ± tr. | 0.1 ± tr. | 0.1 ± tr. |  |
| 20:1n-9 | | 1.9 ± 0.5 | 2.4 ± 0.3 | 1.9 ± 0.5 | 2.0 ± 0.4 |  |
| 20:3n-6 *** | | 0.3 ± tr. | 0.2 ± tr. | 0.8 ± tr. | 0.6 ± 0.1 |  |
| 20:4n-6 *** | | 15.9 ± 0.3 | 14.5 ± 0.4 | 9.5 ± 0.1 | 10.8 ± 1.2 |  |
| 20:3n-3 | | N.D. | N.D. | N.D. | 0.1 ± 0.1 |  |
| 20:5n-3 ** | | N.D. | N.D. | 0.4 ± 0.1 | 0.5 ± 0.2 |  |
| 22:0 | | N.D. | N.D. | N.D. | N.D. |  |
| 22:1n-9 | | 0.1 ± tr. | N.D. | N.D. | N.D. |  |
| 22:4n-6 *** | | 6.7 ± 0.2 | 6.0 ± 0.5 | 2.5 ± 0.1 | 3.5 ± 1.1 |  |
| 22:5n-3 *** | | 0.1 ± tr. | 0.1 ± tr. | 0.6 ± tr. | 0.5 ± 0.2 |  |
| 22:6n-3 *** | | 25.6 ± 0.5 | 25.7 ± 0.7 | 32.6 ± 1.4 | 32.3 ± 1.9 |  |
| 24:0 | | N.D. | N.D. | N.D. | N.D. |  |
| 24:1n-9 | | N.D. | N.D. | N.D. | N.D. |  |
| Other minor FA | | 5.1 ± 0.3 | 6.4 ± 2.3 | 5.2 ± 1.4 | 4.3 ± 0.8 |  |
|  | |  |  |  |  |  |
| Total SAT | | 30.2 ± 0.9 | 30.0 ± 0.5 | 29.7 ± 1.5 | 29.0 ± 0.6 |  |
| Total MUFA | | 15.7 ± 1.2 | 16.8 ± 1.8 | 18.3 ± 1.2 | 18.0 ± 1.0 |  |
| Total PUFA | | 49.0 ± 0.5 | 46.8 ± 1.3 | 46.8 ± 1.3 | 48.6 ± 0.5 |  |
| Total n-3 FA *** | | 25.8 ± 0.5 | 25.9 ± 0.7 | 33.7 ± 1.4 | 33.4 ± 2.2 |  |
| Total n-6 FA *** | | 23.2 ± 0.4 | 21.0 ± 0.9 | 13.1 ± 0.1 | 15.2 ± 2.3 |  |

SFA, saturated fatty acids; MUFA, monounsaturated fatty acids; PUFA, polyunsaturated fatty acids; N.D., not detected; tr., trace (less than 0.05); significant effect of diet, ** p < 0.01, *** p < 0.001; significant effect of genotype, ^♦^ p < 0.05.

**Tab1e 4.** Fatty acid composition of phosphatidylethanolamine (PE) from hippocampus of 16 month-old wild-type (WT) and transgenic (Tg) mice on the oil blend diet (OB) or the DHA diet. Results are represented as mean percentage of total fatty acids ± SEM. Analysis by GLC.

|  | PE Hippocampus - 16 months | | | | | |
| --- | --- | --- | --- | --- | --- | --- |
| Fatty acid | | Tg OB (n = 3) | WT OB (n = 3) | Tg DHA (n = 3) | WT DHA (n = 3) |  |
| 12:0 | | N.D. | N.D. | N.D. | N.D. |  |
| 16:0 | | 6.2 ± 0.3 | 6.7 ± 0.2 | 6.9 ± 0.2 | 7.0 ± 0.3 |  |
| 16:1n-7 ** | | 0.3 ± 0.1 | 0.3 ± tr. | 0.4 ± tr. | 0.4 ± tr. |  |
| 18:0 | | 26.6 ± 1.8 | 24.4 ± 0.4 | 25.8 ± 0.7 | 25.6 ± 0.8 |  |
| 18:1n-9 ** | | 10.3 ± 0.1 | 10.5 ± 0.4 | 12.5 ± 0.4 | 12.2 ± 0.6 |  |
| 18:1n-7 | | 1.5 ± tr. | 1.8 ± 0.2 | 1.4 ± 0.1 | 1.4 ± tr. |  |
| 18:2n-6 * | | 0.3 ± tr. | 0.2 ± tr. | 0.4 ± tr. | 0.3 ± tr. |  |
| 18:3n-6 | | N.D. | N.D. | N.D. | N.D. |  |
| 18:3n-3 | | 0.1 ± tr. | 0.1 ± tr. | 0.1 ± tr. | 0.1 ± tr. |  |
| 20:0 | | 0.1 ± tr. | 0.1 ± tr. | 0.1 ± tr. | 0.1 ± tr. |  |
| 20:1n-9 | | 1.5 ± tr. | 1.4 ± 0.3 | 1.3 ± 0.3 | 1.0 ± 0.1 |  |
| 20:3n-6 *** | | 0.3 ± tr. | 0.3 ± tr. | 0.6 ± tr. | 0.5 ± tr. |  |
| 20:4n-6 *** | | 13.8 ± 0.4 | 13.8 ± 0.5 | 6.7 ± 0.4 | 7.9 ± 0.3 |  |
| 20:3n-3 | | N.D. | N.D. | N.D. | N.D. |  |
| 20:5n-3 *** | | N.D. | N.D. | 0.5 ± tr. | 0.4 ± tr. |  |
| 22:0 | | N.D. | N.D. | N.D. | N.D. |  |
| 22:1n-9 | | N.D. | N.D. | N.D. | N.D. |  |
| 22:4n-6 *** | | 4.7 ± 0.2 | 4.4 ± tr. | 1.1 ± 0.1 | 1.4 ± 0.1 |  |
| 22:5n-3 *** | | 0.1 ± 0.1 | 0.1 ± 0.1 | 0.6 ± tr. | 0.6 ± tr. |  |
| 22:6n-3 *** | | 22.2 ± 1.0 | 24.1 ± 0.5 | 31.6 ± 0.6 | 31.2 ± 0.7 |  |
| 24:0 | | N.D. | N.D. | N.D. | N.D. |  |
| 24:1n-9 | | N.D. | N.D. | N.D. | N.D. |  |
| Other minor FA * | | 12.2 ± 0.3 | 11.9 ± 0.5 | 10.1 ± 0.6 | 9.8 ± 1.2 |  |
|  | |  |  |  |  |  |
| Total SAT | | 32.8 ± 1.6 | 31.2 ± 0.2 | 32.8 ± 0.4 | 32.7 ± 0.6 |  |
| Total MUFA * | | 13.6 ± tr. | 14.0 ± 0.6 | 15.6 ± 0.7 | 15.1 ± 0.6 |  |
| Total PUFA | | 41.4 ± 1.5 | 42.9 ± 0.7 | 41.5 ± 0.9 | 42.4 ± 1.0 |  |
| Total n-3 FA *** | | 22.4 ± 1.0 | 24.3 ± 0.6 | 32.8 ± 0.5 | 32.3 ± 0.8 |  |
| Total n-6 FA *** | | 19.0 ± 0.6 | 18.6 ± 0.4 | 8.7 ± 0.5 | 10.1 ± 0.4 |  |

SFA, saturated fatty acids; MUFA, monounsaturated fatty acids; PUFA, polyunsaturated fatty acids; N.D., not detected; tr., trace (less than 0.05); significant effect of diet, * p < 0.05, ** p < 0.01, *** p < 0.001.

**Tab1e 5.** Fatty acid composition of phosphatidylethanolamine (PE) from cerebellum of 12 month-old wild-type (WT) and transgenic (Tg) mice on the oil blend diet (OB) or the DHA diet. Results are represented as mean percentage of total fatty acids ± SEM. Analysis by GLC.

|  | PE Cerebellum - 12 months | | | | | |
| --- | --- | --- | --- | --- | --- | --- |
| Fatty acid | | Tg OB (n = 4) | WT OB (n = 4) | Tg DHA (n = 4) | WT DHA (n = 4) |  |
| 12:0 | | N.D. | N.D. | N.D. | N.D. |  |
| 16:0 | | 5.1 ± 0.4 | 5.2 ± 0.2 | 5.4 ± 0.5 | 5.9 ± 0.5 |  |
| 16:1n-7 | | 0.3 ± 0.1 | 0.3 ± 0.1 | 0.4 ± 0.1 | 0.6 ± 0.2 |  |
| 18:0 | | 19.2 ± 0.7 | 17.8 ± 1.0 | 18.8 ± 1.3 | 17.7 ± 0.7 |  |
| 18:1n-9 ** | | 17.3 ± 0.4 | 17.8 ± 0.5 | 19.3 ± 0.4 | 19.9 ± 0.9 |  |
| 18:1n-7 | | 2.0 ± 0.1 | 2.6 ± 0.5 | 2.0 ± 0.1 | 2.3 ± tr. |  |
| 18:2n-6 | | 0.4 ± 0.1 | 0.4 ± tr. | 0.5 ± 0.1 | 0.5 ± tr. |  |
| 18:3n-6 | | 0.1 ± tr. | 0.1 ± tr. | 0.1 ± tr. | 0.1 ± tr. |  |
| 18:3n-3 | | 0.1 ± tr. | 0.1 ± 0.1 | 0.1 ± tr. | 0.2 ± tr. |  |
| 20:0 | | 0.4 ± tr. | 0.4 ± 0.1 | 0.2 ± 0.1 | 0.3 ± 0.1 |  |
| 20:1n-9 | | 5.5 ± 0.4 | 6.0 ± tr. | 4.4 ± 0.1 | 5.6 ± 0.2 |  |
| 20:3n-6 *** | | 0.3 ± tr. | 0.3 ± tr. | 0.5 ± tr. | 0.6 ± tr. |  |
| 20:4n-6 *** | | 8.4 ± 0.6 | 8.2 ± 0.1 | 3.3 ± 0.3 | 3.2 ± 0.2 |  |
| 20:3n-3 | | 0.1 ± tr. | N.D. | 0.1 ± 0.1 | 0.1 ± 0.1 |  |
| 20:5n-3 ** | | 0.1 ± tr. | 0.1 ± tr. | 0.3 ± 0.1 | 0.4 ± tr. |  |
| 22:0 | | N.D. | 0.1 ± tr. | 0.1 ± tr. | 0.1 ± tr. |  |
| 22:1n-9 | | 0.2 ± tr. | 0.1 ± tr. | N.D. | 0.1 ± tr. |  |
| 22:4n-6 *** | | 2.6 ± 0.2 | 2.6 ± 0.1 | 0.7 ± 0.1 | 0.7 ± 0.1 |  |
| 22:5n-3 *** | | 0.1 ± tr. | 0.1 ± tr. | 0.4 ± tr. | 0.5 ± tr. |  |
| 22:6n-3 *** | | 22.5 ± 1.8 | 19.4 ± 1.1 | 28.5 ± 1.6 | 27.7 ± 0.3 |  |
| 24:0 | | N.D. | N.D. | N.D. | N.D. |  |
| 24:1n-9 | | N.D. | N.D. | N.D. | N.D. |  |
| Other minor FA | | 15.4 ± 1.9 | 18.5 ± 1.0 | 14.5 ± 1.6 | 13.6 ± 2.4 |  |
|  | |  |  |  |  |  |
| Total SAT | | 24.7 ± 0.7 | 23.5 ± 1.0 | 24.6 ± 0.8 | 24.0 ± 1.2 |  |
| Total MUFA ^♦^ | | 25.2 ± 0.8 | 26.9 ± 0.5 | 26.3 ± 0.4 | 28.4 ± 1.2 |  |
| Total PUFA | | 34.6 ± 2.4 | 31.4 ± 1.4 | 34.7 ± 1.7 | 34.0 ± 0.6 |  |
| Total n-3 FA *** | | 22.9 ± 1.7 | 19.7 ± 1.2 | 29.5 ± 1.6 | 28.9 ± 0.4 |  |
| Total n-6 FA *** | | 11.8 ± 0.8 | 11.6 ± 0.3 | 5.2 ± 0.4 | 5.1 ± 0.2 |  |

SFA, saturated fatty acids; MUFA, monounsaturated fatty acids; PUFA, polyunsaturated fatty acids; N.D., not detected; tr., trace (less than 0.05); significant effect of diet, ** p < 0.01, *** p < 0.001; significant effect of genotype, ^♦^ p < 0.05.

**Tab1e 6.** Fatty acid composition of phosphatidylethanolamine (PE) from cerebellum of 16 month-old wild-type (WT) and transgenic (Tg) mice on the oil blend diet (OB) or the DHA diet. Results are represented as mean percentage of total fatty acids ± SEM. Analysis by GLC.

|  | PE Cerebellum - 16 months | | | | | |
| --- | --- | --- | --- | --- | --- | --- |
| Fatty acid | | Tg OB (n = 3) | WT OB (n = 3) | Tg DHA (n = 3) | WT DHA (n = 3) |  |
| 12:0 | | N.D. | N.D. | N.D. | N.D. |  |
| 16:0 | | 5.2 ± 0.1 | 5.4 ± 0.3 | 5.3 ± 0.2 | 5.7 ± 0.4 |  |
| 16:1n-7 | | 0.4 ± 0.1 | 0.4 ± 0.1 | 0.3 ± tr. | 0.3 ± tr. |  |
| 18:0 * | | 18.5 ± 0.1 | 19.2 ± 0.5 | 17.5 ± 0.6 | 18.2 ± 0.2 |  |
| 18:1n-9 | | 20.1 ± 1.2 | 18.8 ± 0.9 | 21.7 ± 1.1 | 21.4 ± 0.5 |  |
| 18:1n-7 | | 2.5 ± 0.1 | 2.8 ± 0.4 | 2.4 ± 0.2 | 2.7 ± 0.5 |  |
| 18:2n-6 ^♦^ | | 0.4 ± tr. | 0.3 ± tr. | 0.4 ± tr. | 0.3 ± tr. |  |
| 18:3n-6 | | N.D. | N.D. | N.D. | N.D. |  |
| 18:3n-3 | | 0.1 ± tr. | 0.1 ± tr. | 0.1 ± tr. | 0.1 ± tr. |  |
| 20:0 | | 0.2 ± tr. | 0.2 ± 0.1 | 0.2 ± tr. | 0.1 ± tr. |  |
| 20:1n-9 ^♦^ | | 6.0 ± 0.4 | 5.0 ± 0.2 | 5.3 ± 0.3 | 4.8 ± 0.4 |  |
| 20:3n-6 | | 0.3 ± tr. | 0.3 ± tr. | 0.3 ± tr. | 0.4 ± tr. |  |
| 20:4n-6 *** | | 7.5 ± 0.4 | 8.0 ± 0.3 | 2.1 ± 0.2 | 2.7 ± 0.1 |  |
| 20:3n-3 | | N.D. | N.D. | N.D. | N.D. |  |
| 20:5n-3 | | N.D. | N.D. | 0.4 ± tr. | 0.3 ± tr. |  |
| 22:0 | | N.D. | N.D. | N.D. | N.D. |  |
| 22:1n-9 | | N.D. | 0.1 ± tr. | 0.1 ± tr. | N.D. |  |
| 22:4n-6 *** ^♦^ | | 2.6 ± tr. | 2.2 ± tr. | 0.4 ± tr. | 0.5 ± tr. |  |
| 22:5n-3 *** | | N.D. | 0.1 ± tr. | 0.4 ± tr. | 0.4 ± tr. |  |
| 22:6n-3 ** | | 19.1 ± 1.3 | 22.9 ± 0.8 | 27.2 ± 1.1 | 26.5 ± 1.7 |  |
| 24:0 | | N.D. | N.D. | N.D. | N.D. |  |
| 24:1n-9 | | N.D. | N.D. | N.D. | N.D. |  |
| Other minor FA | | 16.9 ± 0.2 | 14.3 ± 0.7 | 15.9 ± 0.6 | 15.5 ± 0.5 |  |
|  | |  |  |  |  |  |
| Total SAT | | 24.0 ± 0.2 | 24.7 ± 0.7 | 23.0 ± 0.5 | 24.0 ± 0.5 |  |
| Total MUFA * | | 29.1 ± 1.6 | 27.1 ± 0.7 | 29.8 ± 1.5 | 29.3 ± 0.7 |  |
| Total PUFA * | | 30.0 ± 1.7 | 33.9 ± 0.5 | 31.3 ± 1.3 | 31.2 ± 1.6 |  |
| Total n-3 FA ** | | 19.2 ± 1.3 | 23.1 ± 0.8 | 28.0 ± 1.1 | 27.3 ± 1.6 |  |
| Total n-6 FA *** | | 10.8 ± 0.5 | 10.8 ± 0.3 | 3.3 ± 0.2 | 3.9 ± 0.2 |  |

SFA, saturated fatty acids; MUFA, monounsaturated fatty acids; PUFA, polyunsaturated fatty acids; N.D., not detected; tr., trace (less than 0.05); significant effect of diet, * p < 0.05, ** p < 0.01, *** p < 0.001; significant effect of genotype, ^♦^ p < 0.05.

**Tab1e 7.** Fatty acid composition of phosphatidylcholine (PC) from cortex of 12 month-old wild-type (WT) and transgenic (Tg) mice on the oil blend diet (OB) or the DHA diet. Results are represented as mean percentage of total fatty acids ± SEM. Analysis by GLC.

|  | PC Cortex - 12 months | | | | | |
| --- | --- | --- | --- | --- | --- | --- |
| Fatty acid | | Tg OB (n = 4) | WT OB (n = 4) | Tg DHA (n = 4) | WT DHA (n = 4) |  |
| 12:0 | | N.D. | N.D. | N.D. | N.D. |  |
| 16:0 | | 40.3 ± 1.6 | 39.1 ± 2.3 | 39.9 ± 2.8 | 40.2 ± 1.8 |  |
| 16:1n-7 | | 0.6 ± 0.1 | 0.6 ± 0.2 | 0.7 ± 0.2 | 0.7 ± 0.2 |  |
| 18:0 | | 13.7 ± 0.6 | 13.6 ± 0.8 | 13.6 ± 0.9 | 13.9 ± 0.5 |  |
| 18:1n-9 | | 22.2 ± 0.3 | 22.5 ± 0.7 | 23.6 ± 1.1 | 23.9 ± 0.6 |  |
| 18:1n-7 * | | 6.4 ± 0.2 | 6.6 ± 0.2 | 5.9 ± 0.2 | 5.8 ± 0.2 |  |
| 18:2n-6 * | | 0.7 ± 0.1 | 0.7 ± tr. | 0.9 ± tr. | 0.8 ± tr. |  |
| 18:3n-6 | | N.D. | N.D. | N.D. | N.D. |  |
| 18:3n-3 | | 0.1 ± tr. | N.D. | N.D. | N.D. |  |
| 20:0 | | 0.1 ± tr. | 0.1 ± tr. | 0.1 ± tr. | 0.1 ± tr. |  |
| 20:1n-9 | | 1.0 ± tr. | 1.0 ± 0.1 | 0.6 ± 0.2 | 1.0 ± 0.1 |  |
| 20:3n-6 *** | | 0.4 ± tr. | 0.4 ± tr. | 1.1 ± 0.1 | 0.9 ± 0.1 |  |
| 20:4n-6 *** | | 7.2 ± 0.4 | 7.5 ± 0.5 | 4.4 ± 0.2 | 3.9 ± 0.2 |  |
| 20:3n-3 | | 0.1 ± tr. | 0.1 ± tr. | 0.1 ± tr. | 0.2 ± 0.1 |  |
| 20:5n-3 *** | | N.D. | N.D. | 0.2 ± tr. | 0.2 ± tr. |  |
| 22:0 * | | 0.1 ± tr. | 0.1 ± tr. | N.D. | 0.1 ± tr. |  |
| 22:1n-9 | | 0.1 ± tr. | 0.1 ± tr. | N.D. | 0.1 ± tr. |  |
| 22:4n-6 *** | | 0.7 ± tr. | 0.7 ± tr. | 0.2 ± tr. | 0.2 ± tr. |  |
| 22:5n-3 * | | N.D. | N.D. | 0.1 ± tr. | 0.1 ± tr. |  |
| 22:6n-3 * | | 5.3 ± 0.6 | 5.7 ± 0.5 | 7.5 ± 0.9 | 6.8 ± 0.5 |  |
| 24:0 | | N.D. | N.D. | N.D. | N.D. |  |
| 24:1n-9 | | N.D. | 0.1 ± 0.1 | N.D. | N.D. |  |
| Other minor FA | | 1.1 ± 0.1 | 1.0 ± tr. | 1.0 ± 0.1 | 1.0 ± 0.1 |  |
|  | |  |  |  |  |  |
| Total SAT | | 54.2 ± 1.4 | 53.0 ± 1.7 | 53.7 ± 2.1 | 54.3 ± 1.4 |  |
| Total MUFA | | 30.2 ± 0.4 | 30.9 ± 0.8 | 30.9 ± 1.0 | 31.5 ± 0.7 |  |
| Total PUFA | | 14.5 ± 1.0 | 15.1 ± 1.0 | 14.4 ± 1.2 | 13.2 ± 0.7 |  |
| Total n-3 FA * | | 5.5 ± 0.6 | 5.8 ± 0.5 | 7.9 ± 0.9 | 7.4 ± 0.5 |  |
| Total n-6 FA *** | | 9.0 ± 0.5 | 9.3 ± 0.5 | 6.6 ± 0.3 | 5.8 ± 0.3 |  |

SFA, saturated fatty acids; MUFA, monounsaturated fatty acids; PUFA, polyunsaturated fatty acids; N.D., not detected; tr., trace (less than 0.05); significant effect of diet, * p < 0.05, *** p < 0.001.

**Tab1e 8.** Fatty acid composition of phosphatidylcholine (PC) from cortex of 16 month-old wild-type (WT) and transgenic (Tg) mice on the oil blend diet (OB) or the DHA diet. Results are represented as mean percentage of total fatty acids ± SEM. Analysis by GLC.

|  | PC Cortex - 16 months | | | | | |
| --- | --- | --- | --- | --- | --- | --- |
| Fatty acid | | Tg OB (n = 3) | WT OB (n = 3) | Tg DHA (n = 3) | WT DHA (n = 3) |  |
| 12:0 | | N.D. | N.D. | N.D. | N.D. |  |
| 16:0 | | 50.7 ± 1.1 | 52.5 ± 0.7 | 52.0 ± 1.2 | 52.2 ± 1.4 |  |
| 16:1n-7 | | 0.5 ± 0.1 | 0.4 ± tr. | 0.5 ± 0.1 | 0.5 ± tr. |  |
| 18:0 | | 13.2 ± 0.4 | 12.6 ± 0.2 | 13.1 ± 0.3 | 12.2 ± 0.3 |  |
| 18:1n-9 | | 20.4 ± 0.5 | 19.7 ± 0.5 | 21.2 ± 0.6 | 21.6 ± 0.9 |  |
| 18:1n-7 | | 5.7 ± 0.1 | 5.6 ± 0.3 | 4.9 ± 0.4 | 5.2 ± 0.4 |  |
| 18:2n-6 | | 0.5 ± tr. | 0.5 ± 0.1 | 0.5 ± tr. | 0.5 ± tr. |  |
| 18:3n-6 | | N.D. | 0.1 ± 0.1 | N.D. | N.D. |  |
| 18:3n-3 | | N.D. | N.D. | N.D. | N.D. |  |
| 20:0 | | 0.1 ± tr. | 0.1 ± tr. | 0.1 ± tr. | 0.1 ± tr. |  |
| 20:1n-9 | | 0.3 ± 0.2 | 0.3 ± 0.1 | 0.3 ± 0.1 | 0.3 ± 0.1 |  |
| 20:3n-6 *** | | 0.2 ± tr. | 0.2 ± tr. | 0.5 ± tr. | 0.4 ± tr. |  |
| 20:4n-6 *** | | 4.9 ± 0.3 | 4.6 ± 0.3 | 1.9 ± 0.1 | 2.4 ± 0.3 |  |
| 20:3n-3 | | N.D. | N.D. | N.D. | N.D. |  |
| 20:5n-3 ** | | N.D. | N.D. | 0.1 ± tr. | 0.1 ± tr. |  |
| 22:0 | | N.D. | N.D. | N.D. | N.D. |  |
| 22:1n-9 | | N.D. | N.D. | N.D. | N.D. |  |
| 22:4n-6 *** | | 0.3 ± tr. | 0.3 ± tr. | N.D. | 0.1 ± tr. |  |
| 22:5n-3 | | N.D. | N.D. | N.D. | N.D. |  |
| 22:6n-3 ** | | 2.7 ± 0.2 | 2.7 ± 0.1 | 4.4 ± 0.4 | 4.0 ± 0.4 |  |
| 24:0 | | N.D. | N.D. | N.D. | N.D. |  |
| 24:1n-9 | | N.D. | N.D. | N.D. | N.D. |  |
| Other minor FA | | 0.6 ± 0.1 | 0.4 ± 0.1 | 0.6 ± 0.1 | 0.5 ± 0.1 |  |
|  | |  |  |  |  |  |
| Total SAT | | 64.0 ± 1.1 | 65.1 ± 0.9 | 65.1 ± 1.3 | 64.5 ± 1.6 |  |
| Total MUFA | | 26.8 ± 0.6 | 26.1 ± 0.7 | 26.9 ± 0.7 | 27.5 ± 1.2 |  |
| Total PUFA | | 8.7 ± 0.5 | 8.4 ± 0.2 | 7.4 ± 0.5 | 7.5 ± 0.7 |  |
| Total n-3 FA ** | | 2.7 ± 0.2 | 2.7 ± 0.1 | 4.5 ± 0.4 | 4.1 ± 0.4 |  |
| Total n-6 FA *** | | 5.9 ± 0.3 | 5.6 ± 0.1 | 2.9 ± 0.2 | 3.4 ± 0.3 |  |

SFA, saturated fatty acids; MUFA, monounsaturated fatty acids; PUFA, polyunsaturated fatty acids; N.D., not detected; tr., trace (less than 0.05); significant effect of diet, ** p < 0.01, *** p < 0.001.

**Tab1e 9.** Fatty acid composition of phosphatidylcholine (PC) from hippocampus of 12 month-old wild-type (WT) and transgenic (Tg) mice on the oil blend diet (OB) or the DHA diet. Results are represented as mean percentage of total fatty acids ± SEM. Analysis by GLC.

|  | PC Hippocampus - 12 months | | | | | |
| --- | --- | --- | --- | --- | --- | --- |
| Fatty acid | | Tg OB (n = 4) | WT OB (n = 4) | Tg DHA (n = 4) | WT DHA (n = 4) |  |
| 12:0 | | N.D. | N.D. | N.D. | N.D. |  |
| 16:0 | | 44.0 ± 0.8 | 46.0 ± 1.2 | 43.7 ± 1.0 | 44.6 ± 1.2 |  |
| 16:1n-7 | | 0.5 ± 0.4 | 1.1 ± 0.4 | 1.3 ± 0.7 | 0.8 ± 0.5 |  |
| 18:0 | | 13.5 ± 0.4 | 13.1 ± 0.3 | 13.3 ± 0.1 | 14.0 ± 0.4 |  |
| 18:1n-9 * | | 20.1 ± 0.3 | 19.3 ± 0.1 | 21.9 ± 0.2 | 21.8 ± 0.7 |  |
| 18:1n-7 | | 9.1 ± 0.3 | 8.5 ± 0.7 | 8.5 ± 0.3 | 8.3 ± 1.4 |  |
| 18:2n-6 ** | | 0.7 ± tr. | 0.6 ± tr. | 0.9 ± 0.1 | 0.7 ± 0.1 |  |
| 18:3n-6 | | N.D. | 0.2 ± 0.2 | N.D. | N.D. |  |
| 18:3n-3 | | N.D. | 0.1 ± tr. | N.D. | N.D. |  |
| 20:0 | | 0.1 ± tr. | 0.1 ± tr. | 0.1 ± tr. | 0.1 ± tr. |  |
| 20:1n-9 ^♦^ | | 0.6 ± tr. | 0.5 ± tr. | 0.6 ± tr. | 0.5 ± 0.1 |  |
| 20:3n-6 *** ^♦^ | | 0.2 ± tr. | 0.2 ± tr. | 0.5 ± tr. | 0.4 ± tr. |  |
| 20:4n-6 *** | | 7.3 ± 0.3 | 6.7 ± 0.2 | 4.2 ± 0.2 | 3.9 ± 0.3 |  |
| 20:3n-3 | | N.D. | N.D. | 0.1 ± 0.1 | N.D. |  |
| 20:5n-3 * | | N.D. | N.D. | 0.1 ± 0.1 | 0.3 ± 0.2 |  |
| 22:0 | | N.D. | N.D. | N.D. | N.D. |  |
| 22:1n-9 | | N.D. | N.D. | N.D. | N.D. |  |
| 22:4n-6 *** | | 0.4 ± 0.1 | 0.3 ± 0.1 | 0.1 ± tr. | 0.1 ± tr. |  |
| 22:5n-3 | | N.D. | N.D. | N.D. | 0.2 ± 0.2 |  |
| 22:6n-3 *** | | 2.7 ± 0.1 | 2.8 ± 0.3 | 3.9 ± 0.1 | 3.7 ± 0.2 |  |
| 24:0 | | N.D. | N.D. | N.D. | N.D. |  |
| 24:1n-9 | | N.D. | N.D. | N.D. | N.D. |  |
| Other minor FA ^♦^ | | 0.8 ± 0.1 | 0.6 ± tr. | 0.8 ± 0.1 | 0.5 ± 0.1 |  |
|  | |  |  |  |  |  |
| Total SAT | | 57.6 ± 0.6 | 59.2 ± 1.4 | 57.1 ± 1.0 | 58.7 ± 1.6 |  |
| Total MUFA * | | 30.3 ± 0.1 | 29.5 ± 0.7 | 32.3 ± 0.8 | 31.3 ± 0.9 |  |
| Total PUFA * | | 11.3 ± 0.3 | 10.7 ± 0.7 | 9.7 ± 0.2 | 9.5 ± 0.5 |  |
| Total n-3 FA *** | | 2.8 ± 0.1 | 2.8 ± 0.2 | 4.1 ± 0.1 | 4.3 ± 0.5 |  |
| Total n-6 FA *** | | 8.5 ± 0.3 | 7.9 ± 0.4 | 5.6 ± 0.3 | 5.2 ± 0.3 |  |

SFA, saturated fatty acids; MUFA, monounsaturated fatty acids; PUFA, polyunsaturated fatty acids; N.D., not detected; tr., trace (less than 0.05); significant effect of diet, * p < 0.05, ** p < 0.01, *** p < 0.001; significant effect of genotype, ^♦^ p < 0.05.

**Tab1e 10.** Fatty acid composition of phosphatidylcholine (PC) from hippocampus of 16 month-old wild-type (WT) and transgenic (Tg) mice on the oil blend diet (OB) or the DHA diet. Results are represented as mean percentage of total fatty acids ± SEM. Analysis by GLC.

|  | PC Hippocampus - 16 months | | | | | |
| --- | --- | --- | --- | --- | --- | --- |
| Fatty acid | | Tg OB (n = 3) | WT OB (n = 3) | Tg DHA (n = 3) | WT DHA (n = 3) |  |
| 12:0 | | N.D. | N.D. | N.D. | N.D. |  |
| 16:0 | | 50.8 ± 0.6 | 51.9 ± 0.6 | 51.8 ± 1.4 | 52.3 ± 0.7 |  |
| 16:1n-7 | | 0.7 ± 0.1 | 0.6 ± 0.1 | 0.6 ± tr. | 0.6 ± 0.1 |  |
| 18:0 | | 14.4 ± 0.5 | 13.9 ± 0.4 | 14.0 ± 0.4 | 13.4 ± 0.3 |  |
| 18:1n-9 *** | | 19.8 ± 0.1 | 19.9 ± 0.2 | 22.1 ± 0.5 | 21.6 ± 0.4 |  |
| 18:1n-7 | | 5.7 ± 0.5 | 5.4 ± 0.5 | 4.5 ± 0.5 | 5.1 ± 0.4 |  |
| 18:2n-6 ** | | 0.4 ± tr. | 0.4 ± tr. | 0.5 ± tr. | 0.5 ± tr. |  |
| 18:3n-6 | | N.D. | N.D. | N.D. | 0.2 ± 0.2 |  |
| 18:3n-3 | | N.D. | N.D. | N.D. | 0.1 ± tr. |  |
| 20:0 | | 0.1 ± tr. | 0.1 ± tr. | 0.1 ± tr. | 0.1 ± tr. |  |
| 20:1n-9 * | | 0.4 ± tr. | 0.4 ± tr. | 0.4 ± tr. | 0.3 ± tr. |  |
| 20:3n-6 *** | | 0.2 ± tr. | 0.2 ± tr. | 0.3 ± tr. | 0.3 ± tr. |  |
| 20:4n-6 *** | | 5.4 ± 0.3 | 5.1 ± 0.3 | 2.5 ± 0.3 | 2.8 ± 0.1 |  |
| 20:3n-3 | | N.D. | N.D. | N.D. | N.D. |  |
| 20:5n-3 ** | | N.D. | N.D. | 0.1 ± tr. | 0.1 ± tr. |  |
| 22:0 | | N.D. | N.D. | N.D. | N.D. |  |
| 22:1n-9 | | N.D. | N.D. | N.D. | N.D. |  |
| 22:4n-6 *** | | 0.2 ± tr. | 0.2 ± tr. | N.D. | N.D. |  |
| 22:5n-3 | | N.D. | N.D. | N.D. | N.D. |  |
| 22:6n-3 *** | | 1.7 ± 0.1 | 1.8 ± 0.1 | 2.8 ± 0.2 | 2.5 ± 0.1 |  |
| 24:0 | | N.D. | N.D. | N.D. | N.D. |  |
| 24:1n-9 | | N.D. | N.D. | N.D. | N.D. |  |
| Other minor FA | | 0.3 ± tr. | 0.2 ± tr, | 0.3 ± 0.1 | 0.3 ± 0.1 |  |
|  | |  |  |  |  |  |
| Total SAT | | 65.3 ± 1.0 | 65.9 ± 0.9 | 65.9 ± 1.2 | 65.7 ± 0.8 |  |
| Total MUFA | | 26.5 ± 0.6 | 26.2 ± 0.7 | 27.6 ± 0.9 | 27.6 ± 0.8 |  |
| Total PUFA * | | 7.9 ± 0.5 | 7.7 ± 0.4 | 6.3 ± 0.5 | 6.4 ± 0.4 |  |
| Total n-3 FA *** | | 1.7 ± 0.1 | 1.8 ± 0.1 | 3.0 ± 0.2 | 2.6 ± 0.2 |  |
| Total n-6 FA *** | | 6.2 ± 0.3 | 5.8 ± 0.3 | 3.3 ± 0.3 | 3.7 ± 0.3 |  |

SFA, saturated fatty acids; MUFA, monounsaturated fatty acids; PUFA, polyunsaturated fatty acids; N.D., not detected; tr., trace (less than 0.05); significant effect of diet, * p < 0.05, ** p < 0.01, *** p < 0.001.

**Tab1e 11.** Fatty acid composition of phosphatidylcholine (PC) from cerebellum of 12 month-old wild-type (WT) and transgenic (Tg) mice on the oil blend diet (OB) or the DHA diet. Results are represented as mean percentage of total fatty acids ± SEM. Analysis by GLC.

|  | PC Cerebellum - 12 months | | | | | |
| --- | --- | --- | --- | --- | --- | --- |
| Fatty acid | | Tg OB (n = 4) | WT OB (n = 4) | Tg DHA (n = 4) | WT DHA (n = 4) |  |
| 12:0 | | N.D. | N.D. | N.D. | N.D. |  |
| 16:0 | | 44.4 ± 0.9 | 44.0 ± 1.8 | 46.2 ± 2.0 | 43.5 ± 0.2 |  |
| 16:1n-7 | | 0.4 ± 0.1 | 0.4 ± 0.1 | 0.4 ± 0.1 | 0.5 ± 0.1 |  |
| 18:0 | | 16.4 ± 0.3 | 16.8 ± 0.2 | 15.8 ± 0.7 | 15.4 ± 0.3 |  |
| 18:1n-9 | | 19.8 ± 0.4 | 19.6 ± 0.5 | 19.3 ± 0.9 | 20.5 ± 0.4 |  |
| 18:1n-7 | | 5.9 ± 0.2 | 6.5 ± 0.8 | 5.1 ± 0.4 | 5.7 ± 0.2 |  |
| 18:2n-6 | | 0.6 ± 0.1 | 0.6 ± tr. | 0.7 ± 0.1 | 0.7 ± 0.1 |  |
| 18:3n-6 | | 0.1 ± tr. | N.D. | 0.1 ± 0.1 | 0.1 ± tr. |  |
| 18:3n-3 | | N.D. | 0.1 ± 0.1 | 0.2 ± tr. | 0.1 ± tr. |  |
| 20:0 | | 0.3 ± tr. | 0.2 ± 0.1 | 0.3 ± 0.1 | 0.3 ± tr. |  |
| 20:1n-9 | | 1.6 ± 0.1 | 1.7 ± 0.2 | 1.4 ± 0.1 | 1.7 ± 0.1 |  |
| 20:3n-6 ** | | 0.2 ± tr. | 0.2 ± tr. | 0.4 ± tr. | 0.4 ± tr. |  |
| 20:4n-6 *** | | 2.6 ± 0.2 | 2.5 ± 0.2 | 0.8 ± 0.1 | 0.8 ± tr. |  |
| 20:3n-3 | | 0.1 ± tr. | 0.1 ± tr. | N.D. | 0.1 ± tr. |  |
| 20:5n-3 | | 0.1 ± tr. | N.D. | 0.1 ± 0.1 | 0.1 ± tr. |  |
| 22:0 | | 0.1 ± tr. | N.D. | 0.1 ± tr. | 0.1 ± tr. |  |
| 22:1n-9 | | 0.1 ± tr. | 0.1 ± tr. | N.D. | 0.1 ± tr. |  |
| 22:4n-6 ** | | 0.3 ± tr. | 0.2 ± 0.1 | N.D. | 0.1 ± tr. |  |
| 22:5n-3 *** ^♦^ | | N.D. | N.D. | N.D. | 0.1 ± tr. |  |
| 22:6n-3 ** | | 6.1 ± 0.4 | 5.8 ± 0.7 | 8.1 ± 0.8 | 8.5 ± 0.3 |  |
| 24:0 | | N.D. | N.D. | N.D. | N.D. |  |
| 24:1n-9 | | N.D. | N.D. | N.D. | N.D. |  |
| Other minor FA | | 1.0 ± 0.2 | 1.1 ± 0.1 | 0.9 ± 0.2 | 1.0 ± 0.1 |  |
|  | |  |  |  |  |  |
| Total SAT | | 61.2 ± 0.7 | 61.0 ± 1.9 | 62.4 ± 2.6 | 59.4 ± 0.5 |  |
| Total MUFA | | 27.8 ± 0.5 | 28.3 ± 1.2 | 26.3 ± 1.5 | 28.6 ± 0.6 |  |
| Total PUFA | | 10.0 ± 0.5 | 9.6 ± 0.9 | 10.6 ± 1.0 | 11.0 ± 0.3 |  |
| Total n-3 FA ** | | 6.3 ± 0.3 | 6.1 ± 0.6 | 8.4 ± 0.8 | 8.9 ± 0.4 |  |
| Total n-6 FA *** | | 3.7 ± 0.3 | 3.5 ± 0.3 | 2.0 ± 0.3 | 2.1 ± 0.1 |  |

SFA, saturated fatty acids; MUFA, monounsaturated fatty acids; PUFA, polyunsaturated fatty acids; N.D., not detected; tr., trace (less than 0.05); significant effect of diet, ** p < 0.01, *** p < 0.001; significant effect of genotype, ^♦^ p < 0.05.

**Tab1e 12.** Fatty acid composition of phosphatidylcholine (PC) from cerebellum of 16 month-old wild-type (WT) and transgenic (Tg) mice on the oil blend diet (OB) or the DHA diet. Results are represented as mean percentage of total fatty acids ± SEM. Analysis by GLC.

|  | PC Cerebellum - 16 months | | | | |
| --- | --- | --- | --- | --- | --- |
| Fatty acid | | Tg OB (n = 3) | WT OB (n = 3) | Tg DHA (n = 3) | WT DHA (n = 3) |
| 12:0 | | N.D. | N.D. | N.D. | N.D. |
| 16:0 | | 43.2 ± 0.3 | 44.2 ± 1.2 | 45.2 ± 0.3 | 45.1 ± 0.8 |
| 16:1n-7 | | 0.7 ± 0.1 | 0.6 ± 0.2 | 0.4 ± tr. | 0.5 ± tr. |
| 18:0 *** | | 17.5 ± 0.3 | 16.8 ± 0.4 | 15.2 ± 0.2 | 15.6 ± 0.1 |
| 18:1n-9 * | | 21.6 ± 0.5 | 20.2 ± 0.2 | 22.3 ± 0.5 | 21.8 ± 0.6 |
| 18:1n-7 * | | 7.2 ± 0.2 | 7.7 ± 0.4 | 5.9 ± 0.5 | 6.7 ± 0.4 |
| 18:2n-6 | | 0.6 ± tr. | 0.5 ± tr. | 0.6 ± tr. | 0.6 ± tr. |
| 18:3n-6 | | N.D. | N.D. | N.D. | N.D. |
| 18:3n-3 | | 0.1 ± tr. | 0.1 ± tr. | N.D. | 0.1 ± tr. |
| 20:0 | | 0.2 ± tr. | 0.2 ± tr. | 0.2 ± tr. | 0.2 ± tr. |
| 20:1n-9 * | | 1.5 ± tr. | 1.4 ± 0.1 | 1.2 ± tr. | 1.2 ± 0.1 |
| 20:3n-6 | | 0.1 ± tr. | 0.1 ± 0.1 | 0.2 ± tr. | 0.2 ± tr. |
| 20:4n-6 *** | | 1.8 ± 0.2 | 1.9 ± 0.3 | 0.3 ± tr. | 0.5 ± tr. |
| 20:3n-3 | | N.D. | N.D. | N.D. | N.D. |
| 20:5n-3 ** | | N.D. | N.D. | 0.1 ± tr. | 0.1 ± tr. |
| 22:0 | | N.D. | N.D. | N.D. | N.D. |
| 22:1n-9 | | N.D. | N.D. | N.D. | 0.1 ± tr. |
| 22:4n-6 | | 0.1 ± 0.1 | 0.1 ± 0.1 | N.D. | N.D. |
| 22:5n-3 | | N.D. | N.D. | N.D. | N.D. |
| 22:6n-3 ** | | 4.6 ± 0.5 | 5.5 ± 0.9 | 7.7 ± 0.4 | 6.8 ± 0.4 |
| 24:0 | | N.D. | N.D. | N.D. | N.D. |
| 24:1n-9 | | N.D. | N.D. | N.D. | N.D. |
| Other minor FA | | 0.6 ± 0.1 | 0.6 ± tr. | 0.5 ± tr. | 0.5 ± tr. |
|  | |  |  |  |  |
| Total SAT | | 61.0 ± 0.2 | 61.3 ± 1.6 | 60.7 ± 0.5 | 60.9 ± 0.7 |
| Total MUFA | | 31.0 ± 0.7 | 29.9 ± 0.4 | 29.8 ± 1.0 | 30.4 ± 0.4 |
| Total PUFA | | 7.4 ± 0.8 | 8.3 ± 1.2 | 9.0 ± 0.5 | 8.3 ± 0.4 |
| Total n-3 FA ** | | 4.7 ± 0.5 | 5.6 ± 0.9 | 7.9 ± 0.4 | 7.0 ± 0.4 |
| Total n-6 FA *** | | 2.7 ± 0.3 | 2.7 ± 0.3 | 1.1 ± 0.1 | 1.3 ± tr. |

SFA, saturated fatty acids; MUFA, monounsaturated fatty acids; PUFA, polyunsaturated fatty acids; N.D., not detected; tr., trace (less than 0.05); significant effect of diet, * p < 0.05, ** p < 0.01, *** p < 0.001.

**Tab1e 13.** Fatty acid composition of phosphatidylserine (PS) from cortex of 12 month-old wild-type (WT) and transgenic (Tg) mice on the oil blend diet (OB) or the DHA diet. Results are represented as mean percentage of total fatty acids ± SEM. Analysis by GLC.

|  | PS Cortex - 12 months | | | | |  |
| --- | --- | --- | --- | --- | --- | --- |
| Fatty acid | | Tg OB (n = 4) | WT OB (n = 4) | Tg DHA (n = 4) | WT DHA (n = 4) | |
| 12:0 | | N.D. | N.D. | N.D. | N.D. | |
| 16:0 * | | 1.3 ± 0.1 | 1.3 ± tr. | 0.9 ± 0.1 | 0.9 ± 0.2 | |
| 16:1n-7 | | 0.5 ± tr. | 0.4 ± 0.1 | 0.5 ± tr. | 0.5 ± tr. | |
| 18:0 | | 44.6 ± 2.0 | 42.8 ± 1.9 | 44.3 ± 1.4 | 45.2 ± 0.3 | |
| 18:1n-9 | | 12.6 ± 1.0 | 12.2 ± 0.7 | 12.5 ± 0.5 | 12.5 ± 0.5 | |
| 18:1n-7 | | 0.6 ± 0.2 | 0.6 ± 0.2 | 0.6 ± 0.2 | 0.8 ± tr. | |
| 18:2n-6 | | 0.2 ± tr. | 0.2 ± tr. | 0.3 ± tr. | 0.2 ± tr. | |
| 18:3n-6 | | N.D. | N.D. | N.D. | N.D. | |
| 18:3n-3 | | 0.1 ± tr. | 0.1 ± tr. | 0.1 ± tr. | 0.1 ± tr. | |
| 20:0 | | 0.3 ± tr. | 0.3 ± tr. | 0.3 ± tr. | 0.3 ± tr. | |
| 20:1n-9 | | 0.7 ± 0.1 | 0.6 ± 0.1 | 0.5 ± tr. | 0.6 ± tr. | |
| 20:3n-6 *** | | 0.3 ± tr. | 0.3 ± 0.1 | 0.6 ± tr. | 0.5 ± tr. | |
| 20:4n-6 ** | | 2.1 ± 0.1 | 2.1 ± 0.3 | 1.3 ± 0.1 | 1.3 ± 0.1 | |
| 20:3n-3 | | 0.3 ± tr. | 0.3 ± tr. | 0.2 ± 0.1 | 0.3 ± tr. | |
| 20:5n-3 ** | | N.D. | N.D. | 0.1 ± tr. | 0.1 ± tr. | |
| 22:0 | | 0.3 ± 0.1 | 0.4 ± 0.1 | 0.4 ± tr. | 0.4 ± tr. | |
| 22:1n-9 | | 0.3 ± 0.1 | 0.4 ± 0.1 | 0.3 ± 0.1 | 0.4 ± 0.1 | |
| 22:4n-6 *** | | 2.8 ± 0.1 | 2.2 ± 0.4 | 1.0 ± tr. | 0.9 ± tr. | |
| 22:5n-3 *** | | 0.1 ± tr. | 0.2 ± 0.1 | 0.4 tr. | 0.4 ± tr. | |
| 22:6n-3 | | 30.8 ± 2.9 | 33.8 ± 3.1 | 34.7 ± 1.7 | 33.4 ± 0.8 | |
| 24:0 | | 0.1 ± tr. | 0.1 ± tr. | 0.2 ± tr. | 0.2 ± tr. | |
| 24:1n-9 | | 0.2 ± 0.1 | 0.1 ± tr. | 0.1 ± tr. | 0.2 ± tr. | |
| Other minor FA *** | | 1.8 ± 0.1 | 1.6 ± 0.2 | 0.7 ± 0.1 | 0.8 ± 0.1 | |
|  | |  |  |  |  | |
| Total SAT | | 46.7 ± 2.1 | 44.9 ± 2.0 | 46.0 ± 1.4 | 47.1 ± 0.3 | |
| Total MUFA | | 14.8 ± 1.0 | 14.2 ± 0.8 | 14.7 ± 0.7 | 15.0 ± 0.6 | |
| Total PUFA | | 36.7 ± 3.0 | 39.3 ± 2.8 | 38.6 ± 1.7 | 37.1 ± 0.7 | |
| Total n-3 FA | | 31.3 ± 2.9 | 34.4 ± 3.2 | 35.5 ± 1.8 | 34.2 ± 0.8 | |
| Total n-6 FA *** | | 5.4 ± 0.1 | 4.9 ± 0.6 | 3.1 ± 0.1 | 2.9 ± 0.1 | |

SFA, saturated fatty acids; MUFA, monounsaturated fatty acids; PUFA, polyunsaturated fatty acids; N.D., not detected; tr., trace (less than 0.05); significant effect of diet, * p < 0.05, ** p < 0.01, *** p < 0.001.

**Tab1e 14.** Fatty acid composition of phosphatidylserine (PS) from cortex of 16 month-old wild-type (WT) and transgenic (Tg) mice on the oil blend diet (OB) or the DHA diet. Results are represented as mean percentage of total fatty acids ± SEM. Analysis by GLC.

|  | PS Cortex - 16 months | | | | | |
| --- | --- | --- | --- | --- | --- | --- |
| Fatty acid | | Tg OB (n = 3) | WT OB (n = 3) | Tg DHA (n = 3) | WT DHA (n = 3) |  |
| 12:0 | | N.D. | N.D. | N.D. | N.D. |  |
| 16:0 | | 1.6 ± tr. | 1.9 ± 0.1 | 1.9 ± 0.1 | 1.6 ± tr. |  |
| 16:1n-7 | | 0.1 ± 0.1 | 0.1 ± 0.1 | 0.1 ± 0.1 | 0.2 ± 0.1 |  |
| 18:0 | | 54.1 ± 1.1 | 52.9 ± 1.5 | 53.4 ± 2.0 | 52.6 ± 1.3 |  |
| 18:1n-9 | | 12.0 ± 0.5 | 12.5 ± 0.3 | 12.3 ± 0.6 | 13.6 ± 0.4 |  |
| 18:1n-7 | | 0.2 ± 0.2 | 0.5 ± 0.2 | 0.2 ± 0.2 | 0.4 ± 0.2 |  |
| 18:2n-6 | | 0.2 ± tr. | 0.1 ± tr. | 0.2 ± tr. | 0.2 ± tr. |  |
| 18:3n-6 | | N.D. | N.D. | N.D. | N.D. |  |
| 18:3n-3 | | 0.1 ± tr. | 0.1 ± tr. | 0.1 ± tr. | 0.1 ± tr. |  |
| 20:0 | | 0.2 ± tr. | 0.2 ± tr. | 0.2 ± tr. | 0.2 ± tr. |  |
| 20:1n-9 | | 0.2 ± 0.1 | 0.2 ± 0.1 | 0.1 ± 0.1 | 0.1 ± 0.1 |  |
| 20:3n-6 *** | | 0.2 ± tr. | 0.2 ± tr. | 0.3 ± tr. | 0.3 ± tr. |  |
| 20:4n-6 *** | | 1.4 ± 0.1 | 1.5 ± 0.1 | 0.6 ± tr. | 0.8 ± tr. |  |
| 20:3n-3 | | N.D. | N.D. | N.D. | N.D. |  |
| 20:5n-3 | | N.D. | N.D. | N.D. | N.D. |  |
| 22:0 | | 0.2 ± tr. | 0.2 ± tr. | 0.1 ± 0.1 | 0.2 ± tr. |  |
| 22:1n-9 | | 0.1 ± tr. | 0.1 ± tr. | N.D. | 0.1 ± tr. |  |
| 22:4n-6 *** | | 1.9 ± tr. | 1.8 ± 0.1 | 0.4 ± tr. | 0.5 ± 0.1 |  |
| 22:5n-3 * ^♦♦^ | | N.D. | N.D. | 0.1 ± 0.1 | 0.2 ± tr. |  |
| 22:6n-3 | | 26.9 ± 1.5 | 27.3 ± 1.1 | 29.7 ± 2.0 | 28.8 ± 1.1 |  |
| 24:0 | | N.D. | N.D. | N.D. | N.D. |  |
| 24:1n-9 | | N.D. | N.D. | N.D. | N.D. |  |
| Other minor FA * | | 0.7 ± tr. | 0.6 ± 02 | 0.3 ± 0.1 | 0.2 ± 0.1 |  |
|  | |  |  |  |  |  |
| Total SAT | | 56.1 ± 1.1 | 55.1 ± 1.4 | 55.7 ± 1.9 | 54.6 ± 1.3 |  |
| Total MUFA | | 12.7 ± 0.7 | 13.3 ± 0.5 | 12.7 ± 0.6 | 14.4 ± 0.3 |  |
| Total PUFA | | 30.6 ± 1.5 | 31.0 ± 1.1 | 31.4 ± 2.1 | 30.8 ± 1.1 |  |
| Total n-3 FA | | 27.0 ± 1.5 | 27.4 ± 1.1 | 29.9 ± 2.1 | 29.0 ± 1.0 |  |
| Total n-6 FA *** | | 3.7 ± 0.1 | 3.6 ± 0.1 | 1.5 ± tr. | 1.8 ± 0.1 |  |

SFA, saturated fatty acids; MUFA, monounsaturated fatty acids; PUFA, polyunsaturated fatty acids; N.D., not detected; tr., trace (less than 0.05); significant effect of diet, * p < 0.05, *** p < 0.001; significant effect of genotype, ^♦♦^ p < 0.01.

**Tab1e 15.** Fatty acid composition of phosphatidylserine (PS) from hippocampus of 12 month-old wild-type (WT) and transgenic (Tg) mice on the oil blend diet (OB) or the DHA diet. Results are represented as mean percentage of total fatty acids ± SEM. Analysis by GLC.

|  | PS Hippocampus - 12 months | | | | | |
| --- | --- | --- | --- | --- | --- | --- |
| Fatty acid | | Tg OB (n = 4) | WT OB (n = 4) | Tg DHA (n = 4) | WT DHA (n = 4) |  |
| 12:0 | | 0.1 ± tr. | N.D. | N.D. | 0.1 ± tr. |  |
| 16:0 | | 2.0 ± 0.4 | 2.0 ± tr. | 2.0 ± 0.3 | 1.8 ± 0.2 |  |
| 16:1n-7 | | 0.4 ± 0.1 | 0.3 ± 0.1 | 0.5 ± 0.1 | 0.3 ± 0.1 |  |
| 18:0 | | 43.8 ± 1.3 | 45.2 ± 1.9 | 42.9 ± 0.9 | 43.2 ± 1.0 |  |
| 18:1n-9 | | 20.2 ± 1.6 | 19.4 ± 1.7 | 20.6 ± 1.2 | 20.3 ± 2.0 |  |
| 18:1n-7 | | N.D. | N.D. | N.D. | N.D. |  |
| 18:2n-6 ** | | 0.3 ± tr. | 0.2 ± tr. | 0.5 ± 0.1 | 0.4 ± tr. |  |
| 18:3n-6 | | N.D. | N.D. | N.D. | N.D. |  |
| 18:3n-3 | | 0.2 ± tr. | 0.1 ± tr. | 0.1 ± tr. | 0.1 ± tr. |  |
| 20:0 | | 0.3 ± tr. | 0.3 ± tr. | 0.3 ± tr. | 0.3 ± 0.1 |  |
| 20:1n-9 | | 0.7 ± 0.1 | 0.6 ± 0.1 | 0.6 ± 0.1 | 0.6 ± 0.1 |  |
| 20:3n-6 *** | | 0.2 ± tr. | 0.2 ± tr. | 0.5 ± tr. | 0.5 ± 0.1 |  |
| 20:4n-6 *** | | 2.8 ± 0.2 | 2.7 ± 0.3 | 1.8 ± 0.2 | 1.7 ± 0.1 |  |
| 20:3n-3 | | 0.1 ± 0.1 | 0.1 ± 0.1 | 0.2 ± 0.1 | 0.2 ± 0.1 |  |
| 20:5n-3 * | | N.D. | N.D. | 0.1 ± tr. | 0.1 ± tr. |  |
| 22:0 | | 0.4 ± tr. | 0.3 ± 0.1 | 0.4 ± 0.1 | 0.4 ± 0.1 |  |
| 22:1n-9 | | 0.2 ± 0.1 | 0.2 ± tr. | 0.4 ± 0.1 | 0.2 ± 0.1 |  |
| 22:4n-6 ** | | 2.0 ± 0.4 | 2.0 ± 0.2 | 0.8 ± 0.1 | 0.9 ± 0.3 |  |
| 22:5n-3 | | N.D. | N.D. | 0.2 ± 0.1 | 0.1 ± 0.1 |  |
| 22:6n-3 | | 25.2 ± 1.8 | 25.3 ± 0.4 | 27.4 ± 1.7 | 28.0 ± 1.1 |  |
| 24:0 | | N.D. | N.D. | N.D. | 0.1 ± 0.1 |  |
| 24:1n-9 | | N.D. | N.D. | 0.1 ± 0.1 | 0.1 ± 0.1 |  |
| Other minor FA | | 1.1 ± 0.4 | 0.9 ± 0.2 | 0.8 ± 0.1 | 0.8 ± tr. |  |
|  | |  |  |  |  |  |
| Total SAT | | 46.6 ± 1.5 | 47.8 ± 1.8 | 45.6 ± 1.0 | 45.7 ± 1.0 |  |
| Total MUFA | | 21.6 ± 1.5 | 20.6 ± 1.9 | 22.2 ± 1.2 | 21.6 ± 1.7 |  |
| Total PUFA | | 30.8 ± 2.3 | 30.7 ± 0.6 | 31.5 ± 1.9 | 31.9 ± 1.4 |  |
| Total n-3 FA *** | | 25.4 ± 1.9 | 25.5 ± 0.4 | 27.9 ± 1.8 | 28.5 ± 1.2 |  |
| Total n-6 FA | | 5.3 ± 0.5 | 5.2 ± 0.3 | 3.6 ± 0.2 | 3.4 ± 0.3 |  |

SFA, saturated fatty acids; MUFA, monounsaturated fatty acids; PUFA, polyunsaturated fatty acids; N.D., not detected; tr., trace (less than 0.05); significant effect of diet, * p < 0.05, ** p < 0.01, *** p < 0.001.

**Tab1e 16.** Fatty acid composition of phosphatidylserine (PS) from hippocampus of 16 month-old wild-type (WT) and transgenic (Tg) mice on the oil blend diet (OB) or the DHA diet. Results are represented as mean percentage of total fatty acids ± SEM. Analysis by GLC.

|  | PS Hippocampus - 16 months | | | | |
| --- | --- | --- | --- | --- | --- |
| Fatty acid | | Tg OB (n = 3) | WT OB (n = 3) | Tg DHA (n = 3) | WT DHA (n = 3) |
| 12:0 | | N.D. | N.D. | N.D. | N.D. |
| 16:0 | | 2.2 ± 0.4 | 2.3 ± 0.3 | 2.8 ± 0.3 | 2.3 ± 0.5 |
| 16:1n-7 | | 0.5 ± 0.1 | 0.3 ± tr. | 0.5 ± 0.1 | 0.5 ± 0.1 |
| 18:0 | | 54.6 ± 0.8 | 54.2 ± 0.2 | 55.0 ± 0.3 | 53.2 ± 1.0 |
| 18:1n-9 | | 16.5 ± 0.3 | 17.4 ± 0.5 | 15.9 ± 0.9 | 16.8 ± 1.0 |
| 18:1n-7 | | 0.5 ± 0.3 | N.D. | 0.2 ± 0.2 | 0.2 ± 0.2 |
| 18:2n-6 ** | | 0.2 ± tr. | 0.2 ± tr. | 0.3 ± tr. | 0.3 ± 0.1 |
| 18:3n-6 | | N.D. | N.D. | N.D. | N.D. |
| 18:3n-3 | | 0.2 ± 0.1 | 0.2 ± 0.1 | 0.2 ± 0.1 | 0.3 ± tr. |
| 20:0 | | 0.2 ± 0.1 | 0.3 ± tr. | 0.2 ± tr. | 0.3 ± tr. |
| 20:1n-9 | | 0.4 ± tr. | 0.3 ± tr. | 0.3 ± tr. | 0.3 ± tr. |
| 20:3n-6 ** | | 0.2 ± tr. | 0.2 ± tr. | 0.3 ± tr. | 0.3 ± tr. |
| 20:4n-6 ** | | 1.7 ± 0.1 | 1.7 ± tr. | 0.8 ± 0.1 | 1.0 ± 0.2 |
| 20:3n-3 | | N.D. | N.D. | N.D. | N.D. |
| 20:5n-3 | | N.D. | N.D. | N.D. | N.D. |
| 22:0 | | N.D. | 0.1 ± 0.1 | 0.1 ± 0.1 | 0.1 ± 0.1 |
| 22:1n-9 * | | 0.2 ± 0.1 | 0.2 ± tr. | 0.1 ± 0.1 | N.D. |
| 22:4n-6 *** | | 2.0 ± 0.1 | 1.7 ± 0.1 | 0.5 ± tr. | 0.5 ± 0.1 |
| 22:5n-3 | | N.D. | N.D. | N.D. | 0.1 ± 0.1 |
| 22:6n-3 ** | | 20.1 ± 0.2 | 20.8 ± 0.5 | 22.4 ± 1.0 | 23.4 ± 0.7 |
| 24:0 | | N.D. | N.D. | N.D. | N.D. |
| 24:1n-9 | | N.D. | N.D. | N.D. | N.D. |
| Other minor FA * ^♦♦^ | | 0.6 ± tr. | 0.2 ± 0.1 | 0.3 ± 0.2 | 0.4 ± 0.1 |
|  | |  |  |  |  |
| Total SAT | | 56.9 ± 0.6 | 56.8 ± 0.5 | 58.2 ± 0.4 | 56.0 ± 0.7 |
| Total MUFA | | 18.1 ± 0.5 | 18.3 ± 0.5 | 17.1 ± 1.0 | 17.8 ± 1.0 |
| Total PUFA | | 24.4 ± 0.2 | 24.7 ± 0.6 | 24.5 ± 1.1 | 25.8 ± 1.1 |
| Total n-3 FA ** | | 20.3 ± 0.1 | 20.9 ± 0.5 | 22.6 ± 1.0 | 23.7 ± 0.8 |
| Total n-6 FA *** | | 4.1 ± 0.2 | 3.7 ± 0.1 | 1.9 ± 0.1 | 2.1 ± 0.4 |

SFA, saturated fatty acids; MUFA, monounsaturated fatty acids; PUFA, polyunsaturated fatty acids; N.D., not detected; tr., trace (less than 0.05); significant effect of diet, * p < 0.05, ** p < 0.01, *** p < 0.001; significant effect of genotype, ^♦♦^ p < 0.01.

**Tab1e 17.** Fatty acid composition of phosphatidylserine (PS) from cerebellum of 12 month-old wild-type (WT) and transgenic (Tg) mice on the oil blend diet (OB) or the DHA diet. Results are represented as mean percentage of total fatty acids ± SEM. Analysis by GLC.

|  | PS Cerebellum - 12 months | | | | | |
| --- | --- | --- | --- | --- | --- | --- |
| Fatty acid | | Tg OB (n = 4) | WT OB (n = 4) | Tg DHA (n = 4) | WT DHA (n = 4) |  |
| 12:0 | | N.D. | N.D. | N.D. | N.D. |  |
| 16:0 | | 2.1 ± 0.3 | 2.0 ± 0.3 | 1.9 ± 0.3 | 2.3 ± 0.3 |  |
| 16:1n-7 | | 0.3 ± 0.2 | 0.3 ± 0.1 | 0.4 ± 0.1 | 0.6 ± 0.2 |  |
| 18:0 | | 45.3 ± 2.3 | 44.2 ± 1.5 | 41.2 ± 1.4 | 41.9 ± 1.4 |  |
| 18:1n-9 | | 24.9 ± 0.7 | 26.1 ± 1.3 | 25.8 ± 1.1 | 26.3 ± 1.2 |  |
| 18:1n-7 | | 0.5 ± 0.3 | 0.6 ± 0.3 | 0.5 ± 0.3 | 0.8 ± 0.3 |  |
| 18:2n-6 | | 0.4 ± 0.1 | 0.4 ± 0.1 | 0.4 ± tr. | 0.5 ± 0.1 |  |
| 18:3n-6 | | 0.1 ± 0.1 | 0.1 ± 0.1 | 0.3 ± 0.1 | 0.2 ± tr. |  |
| 18:3n-3 | | 0.2 ± 0.1 | 0.6 ± 0.4 | 0.1 ± 0.1 | 0.2 ± tr. |  |
| 20:0 | | 0.8 ± 0.1 | 0.7 ± 0.2 | 0.9 ± 0.3 | 0.7 ± 0.1 |  |
| 20:1n-9 ^♦^ | | 1.9 ± 0.3 | 2.2 ± 0.2 | 1.6 ± 0.2 | 2.3 ± 0.1 |  |
| 20:3n-6 * | | 0.3 ± 0.1 | 0.3 ± 0.1 | 0.4 ± tr. | 0.6 ± 0.1 |  |
| 20:4n-6 *** | | 2.0 ± 0.2 | 1.9 ± 0.2 | 1.2 ± 0.2 | 1.0 ± 0.1 |  |
| 20:3n-3 | | 0.1 ± 0.1 | 0.1 ± 0.1 | 0.2 ± 0.1 | 0.3 ± 0.1 |  |
| 20:5n-3 | | 0.2 ± tr. | 0.3 ± 0.1 | 0.3 ± 0.1 | 0.2 ± 0.1 |  |
| 22:0 | | 0.5 ± 0.1 | 0.5 ± 0.1 | 0.5 ± 0.2 | 0.7 ± 0.2 |  |
| 22:1n-9 | | 0.4 ± 0.1 | 0.5 ± 0.3 | 0.6 ± 0.1 | 0.5 ± tr. |  |
| 22:4n-6 *** | | 1.6 ± 0.1 | 1.5 ± 0.1 | 0.6 ± tr. | 0.5 ± 0.1 |  |
| 22:5n-3 *** | | N.D. | N.D. | 0.2 ± 0.1 | 0.3 ± 0.1 |  |
| 22:6n-3 ** | | 17.3 ± 1.3 | 16.5 ± 0.9 | 21.6 ± 0.9 | 18.8 ± 0.7 |  |
| 24:0 | | 0.1 ± 0.1 | N.D. | N.D. | N.D. |  |
| 24:1n-9 | | 0.1 ± tr. | N.D. | 0.1 ± 0.1 | N.D. |  |
| Other minor FA | | 0.9 ± 0.2 | 1.2 ± 0.4 | 1.3 ± 0.2 | 1.3 ± 0.2 |  |
|  | |  |  |  |  |  |
| Total SAT | | 48.9 ± 2.1 | 47.5 ± 1.2 | 44.5 ± 1.4 | 45.6 ± 1.1 |  |
| Total MUFA | | 28.2 ± 1.3 | 29.7 ± 1.1 | 28.9 ± 1.6 | 30.5 ± 1.6 |  |
| Total PUFA | | 22.1 ± 1.6 | 21.7 ± 1.3 | 25.3 ± 1.0 | 22.6 ± 1.0 |  |
| Total n-3 FA ** | | 17.8 ± 1.3 | 17.5 ± 1.2 | 22.5 ± 0.9 | 19.7 ± 0.8 |  |
| Total n-6 FA ** | | 4.3 ± 0.4 | 4.1 ± 0.4 | 2.9 ± 0.1 | 2.9 ± 0.3 |  |

SFA, saturated fatty acids; MUFA, monounsaturated fatty acids; PUFA, polyunsaturated fatty acids; N.D., not detected; tr., trace (less than 0.05); significant effect of diet, * p < 0.05, ** p < 0.01, *** p < 0.001; significant effect of genotype, ^♦^ p < 0.05.

**Tab1e 18.** Fatty acid composition of phosphatidylserine (PS) from cerebellum of 16 month-old wild-type (WT) and transgenic (Tg) mice on the oil blend diet (OB) or the DHA diet. Results are represented as mean percentage of total fatty acids ± SEM. Analysis by GLC.

|  | PS Cerebellum - 16 months | | | | | |
| --- | --- | --- | --- | --- | --- | --- |
| Fatty acid | | Tg OB (n = 3) | WT OB (n = 3) | Tg DHA (n = 3) | WT DHA (n = 3) |  |
| 12:0 | | N.D. | N.D. | N.D. | N.D. |  |
| 16:0 | | 2.9 ± 0.5 | 2.7 ± 0.7 | 2.6 ± 0.2 | 2.4 ± 0.1 |  |
| 16:1n-7 | | 0.9 ± 0.5 | 0.5 ± 0.2 | 0.4 ± tr. | 0.4 ± tr. |  |
| 18:0 | | 45.4 ± 1.3 | 44.3 ± 1.1 | 42.8 ± 0.5 | 42.8 ± 1.2 |  |
| 18:1n-9 | | 32.3 ± 0.7 | 31.2 ± 0.5 | 31.8 ± 1.2 | 32.5 ± 0.9 |  |
| 18:1n-7 | | 0.4 ± 0.4 | 0.7 ± 0.3 | 0.7 ± 0.3 | 0.4 ± 0.4 |  |
| 18:2n-6 | | 0.3 ± 0.1 | 0.3 ± 0.1 | 0.3 ± tr. | 0.3 ± tr. |  |
| 18:3n-6 | | N.D. | N.D. | N.D. | N.D. |  |
| 18:3n-3 | | 0.2 ± 0.1 | 0.3 ± 0.1 | 0.2 ± tr. | 0.2 ± tr. |  |
| 20:0 | | 0.5 ± 0.1 | 0.5 ± tr. | 0.4 ± 0.1 | 0.4 ± tr. |  |
| 20:1n-9 * | | 2.3 ± 0.3 | 1.8 ± 0.1 | 1.5 ± 0.3 | 1.3 ± 0.1 |  |
| 20:3n-6 | | 0.1 ± 0.1 | 0.2 ± tr. | 0.2 ± tr. | 0.3 ± tr. |  |
| 20:4n-6 ** | | 1.3 ± 0.2 | 1.5 ± 0.1 | 0.6 ± 0.1 | 0.8 ± 0.1 |  |
| 20:3n-3 | | N.D. | N.D. | N.D. | 0.1 ± 0.1 |  |
| 20:5n-3 | | N.D. | N.D. | 0.1 ± tr. | N.D. |  |
| 22:0 | | 0.5 ± tr. | 0.6 ± tr. | 0.5 ± 0.1 | 0.6 ± tr. |  |
| 22:1n-9 | | 0.4 ± tr. | 0.3 ± tr. | 0.3 ± tr. | 0.4 ± tr. |  |
| 22:4n-6 *** | | 0.9 ± 0.2 | 1.1 ± 0.1 | 0.2 ± 0.1 | 0.2 ± 0.1 |  |
| 22:5n-3 *** | | N.D. | N.D. | 0.2 ± tr. | 0.2 ± tr. |  |
| 22:6n-3 * | | 11.1 ± 2.1 | 13.8 ± 1.9 | 16.5 ± 1.3 | 15.9 ± 0.7 |  |
| 24:0 * | | N.D. | N.D. | 0.1 ± 0.1 | 0.2 ± 0.1 |  |
| 24:1n-9 ** | | N.D. | 0.1 ± 0.1 | 0.1 ± 0.1 | 0.2 ± tr. |  |
| Other minor FA | | 0.3 ± 0.1 | 0.2 ± 0.1 | 0.4 ± 0.1 | 0.3 ± 0.1 |  |
|  | |  |  |  |  |  |
| Total SAT | | 49.4 ± 1.9 | 48.0 ± 1.8 | 46.4 ± 0.7 | 46.5 ± 1.0 |  |
| Total MUFA | | 36.2 ± 0.7 | 34.7 ± 0.3 | 34.8 ± 1.3 | 35.3 ± 1.1 |  |
| Total PUFA | | 14.1 ± 2.4 | 17.1 ± 1.9 | 18.3 ± 1.5 | 17.9 ± 0.6 |  |
| Total n-3 FA * | | 11.4 ± 2.1 | 14.1 ± 1.8 | 16.9 ± 1.3 | 16.3 ± 0.7 |  |
| Total n-6 FA *** | | 2.7 ± 0.3 | 3.0 ± 0.2 | 1.4 ± 0.2 | 1.6 ± 0.2 |  |

SFA, saturated fatty acids; MUFA, monounsaturated fatty acids; PUFA, polyunsaturated fatty acids; N.D., not detected; tr., trace (less than 0.05); significant effect of diet, * p < 0.05, ** p < 0.01, *** p < 0.001.

**Tab1e 19.** Fatty acid composition of phosphatidylinositol (PI) from cortex of 12 month-old wild-type (WT) and transgenic (Tg) mice on the oil blend diet (OB) or the DHA diet. Results are represented as mean percentage of total fatty acids ± SEM. Analysis by GLC.

|  | PI Cortex - 12 months | | | | | |
| --- | --- | --- | --- | --- | --- | --- |
| Fatty acid | | Tg OB (n = 4) | WT OB (n = 4) | Tg DHA (n = 4) | WT DHA (n = 4) |  |
| 12:0 | | N.D. | N.D. | N.D. | N.D. |  |
| 16:0 | | 6.0 ± 1.1 | 5.4 ± 0.9 | 6.3 ± 0.7 | 7.5 ± 0.7 |  |
| 16:1n-7 | | 1.1 ± 0.1 | 1.0 ± 0.1 | 0.9 ± 0.3 | 1.2 ± 0.1 |  |
| 18:0 | | 35.3 ± 1.8 | 34.4 ± 1.8 | 34.5 ± 1.5 | 37.0 ± 0.7 |  |
| 18:1n-9 | | 5.8 ± 0.2 | 5.4 ± 0.3 | 6.1 ± 0.2 | 6.0 ± 0.2 |  |
| 18:1n-7 | | 2.5 ± tr. | 2.4 ± 0.1 | 2.6 ± 0.1 | 2.0 ± 0.7 |  |
| 18:2n-6 * | | 0.7 ± tr. | 0.6 ± tr. | 0.8 ± 0.1 | 0.8 ± tr. |  |
| 18:3n-6 | | N.D. | N.D. | 0.2 ± 0.2 | N.D. |  |
| 18:3n-3 | | 0.1 ± tr. | 0.1 ± tr. | 0.2 ± tr. | 0.2 ± 0.1 |  |
| 20:0 | | 0.1 ± tr. | 0.1 ± tr. | 0.1 ± tr. | 0.1 ± tr. |  |
| 20:1n-9 | | 0.7 ± 0.1 | 0.6 ± 0.1 | 0.6 ± tr. | 0.6 ± 0.1 |  |
| 20:3n-6 *** | | 0.3 ± tr. | 0.3 ± 0.1 | 1.1 ± 0.1 | 0.9 ± 0.1 |  |
| 20:4n-6** | | 38.7 ± 2.3 | 41.6 ± 2.8 | 33.5 ± 2.5 | 32.2 ± 0.9 |  |
| 20:3n-3 ^♦^ | | 1.9 ± 0.4 | 1.3 ± 0.3 | 2.1 ± 0.6 | 0.7 ± 0.2 |  |
| 20:5n-3 *** | | N.D. | 0.1 ± tr. | 2.0 ± 0.4 | 1.8 ± 0.1 |  |
| 22:0 | | N.D. | 0.7 ± 0.4 | 0.3 ± 0.1 | 0.1 ± 0.1 |  |
| 22:1n-9 | | 1.9 ± 0.6 | 0.6 ± 0.4 | 0.6 ± 0.4 | 0.2 ± 0.1 |  |
| 22:4n-6 | | 0.6 ± 0.4 | 1.0 ± 0.4 | 1.5 ± 0.8 | 2.2 ± 1.1 |  |
| 22:5n-3 | | N.D. | N.D. | 0.1 ± tr. | N.D. |  |
| 22:6n-3 | | 2.6 ± 0.5 | 3.0 ± 0.4 | 3.4 ± 0.5 | 3.5 ± 0.2 |  |
| 24:0 | | 0.1 ± 0.1 | 0.1 ± 0.1 | 0.6 ± 0.5 | 0.8 ± 0.7 |  |
| 24:1n-9 | | N.D. | N.D. | 0.3 ± 0.3 | 0.4 ± 0.4 |  |
| Other minor FA | | 1.6 ± 0.3 | 1.3 ± 0.3 | 2.2 ± 0.4 | 1.9 ± 0.5 |  |
|  | |  |  |  |  |  |
| Total SAT | | 41.5 ± 2.5 | 40.7 ± 2.6 | 41.9 ± 1.8 | 45.5 ± 0.9 |  |
| Total MUFA | | 11.9 ± 0.9 | 10.0 ± 0.8 | 11.1 ± 1.0 | 10.3 ± 0.8 |  |
| Total PUFA | | 44.9 ± 2.9 | 48.0 ± 3.1 | 44.8 ± 2.4 | 42.3 ± 0.8 |  |
| Total n-3 FA ** | | 4.6 ± 0.7 | 4.5 ± 0.6 | 7.8 ± 1.1 | 6.1 ± 0.4 |  |
| Total n-6 FA * | | 40.3 ± 2.4 | 43.5 ± 2.5 | 37.0 ± 1.9 | 36.2 ± 0.6 |  |

SFA, saturated fatty acids; MUFA, monounsaturated fatty acids; PUFA, polyunsaturated fatty acids; N.D., not detected; tr., trace (less than 0.05); significant effect of diet, * p < 0.05, ** p < 0.01, *** p < 0.001; significant effect of genotype, ^♦^ p < 0.05.

**Tab1e 20.** Fatty acid composition of phosphatidylinositol (PI) from cortex of 16 month-old wild-type (WT) and transgenic (Tg) mice on the oil blend diet (OB) or the DHA diet. Results are represented as mean percentage of total fatty acids ± SEM. Analysis by GLC.

|  | PI Cortex - 16 months | | | | | |
| --- | --- | --- | --- | --- | --- | --- |
| Fatty acid | | Tg OB (n = 3) | WT OB (n = 3) | Tg DHA (n = 3) | WT DHA (n = 3) |  |
| 12:0 | | N.D. | N.D. | N.D. | N.D. |  |
| 16:0 | | 10.1 ± 1.0 | 8.5 ± 0.6 | 9.1 ± 0.5 | 9.6 ± 0.3 |  |
| 16:1n-7 | | 0.8 ± 0.4 | 0.4 ± 0.2 | 0.7 ± 0.5 | 0.3 ± 0.3 |  |
| 18:0 | | 45.4 ± 1.2 | 45.8 ± 1.6 | 46.7 ± 1.6 | 45.0 ± 1.8 |  |
| 18:1n-9 ** | | 5.9 ± 0.4 | 5.7 ± tr. | 7.1 ± 0.2 | 6.5 ± 0.1 |  |
| 18:1n-7 | | 1.6 ± 0.1 | 1.6 ± tr. | 1.6 ± 0.1 | 1.7 ± 0.2 |  |
| 18:2n-6 | | 0.4 ± 0.1 | 0.3 ± tr. | 0.6 ± 0.1 | 0.4 ± tr. |  |
| 18:3n-6 | | N.D. | N.D. | N.D. | N.D. |  |
| 18:3n-3 | | 0.2 ± 0.1 | 0.1 ± 0.1 | 0.1 ± 0.1 | 0.2 ± 0.1 |  |
| 20:0 | | 0.1 ± 0.1 | N.D. | 0.1 ± 0.1 | 0.1 ± 0.1 |  |
| 20:1n-9 | | N.D. | N.D. | N.D. | 0.1 ± 0.1 |  |
| 20:3n-6 *** | | N.D. | N.D. | 0.8 ± tr. | 0.7 ± tr. |  |
| 20:4n-6 * | | 33.0 ± 1.9 | 35.2 ± 0.8 | 28.1 ± 2.4 | 30.9 ± 1.6 |  |
| 20:3n-3 | | N.D. | N.D. | N.D. | N.D. |  |
| 20:5n-3 *** | | N.D. | N.D. | 1.7 ± 0.2 | 1.2 ± 0.1 |  |
| 22:0 | | N.D. | N.D. | N.D. | N.D. |  |
| 22:1n-9 | | N.D. | N.D. | N.D. | N.D. |  |
| 22:4n-6 | | N.D. | N.D. | N.D. | N.D. |  |
| 22:5n-3 | | N.D. | N.D. | N.D. | N.D. |  |
| 22:6n-3 *** | | 1.3 ± 0.1 | 1.7 ± 0.1 | 2.6 ± 0.1 | 2.5 ± tr. |  |
| 24:0 | | N.D. | N.D. | N.D. | N.D. |  |
| 24:1n-9 | | N.D. | N.D. | N.D. | N.D. |  |
| Other minor FA | | 1.2 ± 0.6 | 0.7 ± 0.2 | 0.8 ± 0.3 | 0.9 ± tr. |  |
|  | |  |  |  |  |  |
| Total SAT | | 55.6 ± 1.8 | 54.3 ± 1.0 | 55.9 ± 2.1 | 54.6 ± 1.7 |  |
| Total MUFA | | 8.3 ± 0.6 | 7.6 ± 0.2 | 9.4 ± 0.6 | 8.6 ± 0.3 |  |
| Total PUFA | | 34.9 ± 2.0 | 37.4 ± 0.7 | 33.9 ± 2.7 | 35.9 ± 1.6 |  |
| Total n-3 FA *** | | 1.5 ± 0.1 | 1.9 ± 0.1 | 4.4 ± 0.2 | 3.9 ± tr. |  |
| Total n-6 FA | | 33.4 ± 2.0 | 35.5 ± 0.8 | 29.5 ± 2.5 | 32.0 ± 1.6 |  |

SFA, saturated fatty acids; MUFA, monounsaturated fatty acids; PUFA, polyunsaturated fatty acids; N.D., not detected; tr., trace (less than 0.05); significant effect of diet, * p < 0.05, ** p < 0.01, *** p < 0.001.

**Tab1e 21.** Fatty acid composition of phosphatidylinositol (PI) from hippocampus of 12 month-old wild-type (WT) and transgenic (Tg) mice on the oil blend diet (OB) or the DHA diet. Results are represented as mean percentage of total fatty acids ± SEM. Analysis by GLC.

|  | PI Hippocampus - 12 months | | | | | |
| --- | --- | --- | --- | --- | --- | --- |
| Fatty acid | | Tg OB (n = 4) | WT OB (n = 4) | Tg DHA (n = 4) | WT DHA (n = 4) |  |
| 12:0 | | 0.2 ± 0.2 | 0.1 ± 0.1 | 0.2 ± 0.2 | 0.1 ± 0.1 |  |
| 16:0 | | 7.9 ± 1.2 | 9.1 ± 1.0 | 7.0 ± 1.7 | 6.7 ± 1.5 |  |
| 16:1n-7 | | 0.9 ± 0.1 | 0.6 ± 0.1 | 0.9 ± 0.2 | 1.0 ± 0.3 |  |
| 18:0 | | 40.0 ± 1.2 | 40.7 ± 2.7 | 41.0 ± 1.1 | 40.6 ± 1.3 |  |
| 18:1n-9 | | 9.7 ± 0.6 | 8.6 ± 0.7 | 10.4 ± 0.9 | 9.3 ± 1.2 |  |
| 18:1n-7 | | 3.8 ± 0.7 | 3.5 ± 0.1 | 3.5 ± 0.8 | 3.0 ± 1.2 |  |
| 18:2n-6 | | 0.7 ± tr. | 0.7 ± 0.1 | 0.9 ± 0.1 | 0.8 ± 0.1 |  |
| 18:3n-6 | | 0.5 ± 0.2 | 0.4 ± 0.1 | 0.3 ± 0.2 | 0.2 ± 0.1 |  |
| 18:3n-3 | | 0.1 ± 0.1 | 0.1 ± 0.1 | N.D. | N.D. |  |
| 20:0 | | 0.1 ± tr. | 0.1 ± 0.1 | N.D. | 0.1 ± 0.1 |  |
| 20:1n-9 | | 0.2 ± 0.1 | 0.3 ± 0.1 | 0.4 ± 0.2 | 0.4 ± 0.3 |  |
| 20:3n-6 ** | | N.D. | N.D. | 0.3 ± 0.1 | 0.4 ± 0.2 |  |
| 20:4n-6 | | 32.4 ± 1.7 | 33.1 ± 1.8 | 29.2 ± 1.3 | 30.5 ± 1.2 |  |
| 20:3n-3 | | N.D. | N.D. | 0.5 ± 0.5 | 0.6 ± 0.6 |  |
| 20:5n-3 *** | | N.D. | N.D. | 1.4 ± 0.2 | 1.5 ± 0.2 |  |
| 22:0 | | 0.1 ± 0.1 | 0.3 ± 0.1 | N.D. | 0.2 ± 0.2 |  |
| 22:1n-9 | | 0.2 ± 0.2 | N.D. | 0.4 ± 0.4 | 0.3 ± 0.3 |  |
| 22:4n-6 | | 0.5 ± 0.4 | N.D. | 0.3 ± 0.3 | 0.3 ± 0.3 |  |
| 22:5n-3 | | N.D. | N.D. | N.D. | N.D. |  |
| 22:6n-3 | | 1.0 ± 0.3 | 1.2 ± 0.2 | 1.5 ± 0.5 | 2.2 ± 0.4 |  |
| 24:0 | | N.D. | N.D. | N.D. | N.D. |  |
| 24:1n-9 | | N.D. | N.D. | N.D. | N.D. |  |
| Other minor FA | | 1.9 ± 0.3 | 1.2 ± 0.3 | 1.5 ± 0.2 | 1.9 ± 0.5 |  |
|  | |  |  |  |  |  |
| Total SAT | | 48.2 ± 1.2 | 50.1 ± 3.7 | 48.3 ± 1.3 | 47.7 ± 0.7 |  |
| Total MUFA | | 14.8 ± 1.0 | 13.0 ± 1.5 | 15.7 ± 1.2 | 13.9 ± 1.6 |  |
| Total PUFA | | 35.1 ± 2.1 | 35.7 ± 2.1 | 34.5 ± 2.2 | 36.5 ± 2.1 |  |
| Total n-3 FA ** | | 1.0 ± 0.3 | 1.4 ± 0.1 | 3.4 ± 0.9 | 4.3 ± 0.9 |  |
| Total n-6 FA | | 34.0 ± 1.9 | 34.3 ± 2.1 | 31.1 ± 1.4 | 32.2 ± 1.3 |  |

SFA, saturated fatty acids; MUFA, monounsaturated fatty acids; PUFA, polyunsaturated fatty acids; N.D., not detected; tr., trace (less than 0.05); significant effect of diet, ** p < 0.01, *** p < 0.001.

**Tab1e 22.** Fatty acid composition of phosphatidylinositol (PI) from hippocampus of 16 month-old wild-type (WT) and transgenic (Tg) mice on the oil blend diet (OB) or the DHA diet. Results are represented as mean percentage of total fatty acids ± SEM. Analysis by GLC.

|  | PI Hippocampus - 16 months | | | | | |
| --- | --- | --- | --- | --- | --- | --- |
| Fatty acid | | Tg OB (n = 3) | WT OB (n = 3) | Tg DHA (n = 3) | WT DHA (n = 3) |  |
| 12:0 | | N.D. | 0.1 ± 0.1 | N.D. | N.D. |  |
| 16:0 | | 8.9 ± 1.1 | 9.7 ± 1.4 | 10.7 ± 0.9 | 10.4 ± 1.2 |  |
| 16:1n-7 ^♦^ | | 1.8 ± 0.1 | 2.5 ± 0.2 | 1.5 ± 0.5 | 2.9 ± 0.6 |  |
| 18:0 | | 51.0 ± 2.4 | 48.3 ± 1.8 | 51.0 ± 1.8 | 48.1 ± 2.6 |  |
| 18:1n-9 * | | 6.4 ± 0.9 | 6.6 ± 1.2 | 9.1 ± 0.5 | 8.6 ± 1.1 |  |
| 18:1n-7 | | 0.5 ± 0.5 | 0.9 ± 0.5 | 1.4 ± tr. | 1.3 ± 0.1 |  |
| 18:2n-6 | | 0.9 ± 0.1 | 0.8 ± 0.1 | 1.0 ± 0.2 | 1.0 ± 0.1 |  |
| 18:3n-6 | | N.D. | N.D. | N.D. | N.D. |  |
| 18:3n-3 | | 0.7 ± 0.4 | 0.4 ± 0.2 | 0.6 ± 0.3 | 0.7 ± 0.1 |  |
| 20:0 | | 0.6 ± 0.5 | 0.1 ± 0.1 | 0.1 ± 0.1 | N.D. |  |
| 20:1n-9 | | N.D. | N.D. | N.D. | N.D. |  |
| 20:3n-6 | | N.D. | N.D. | 0.1 ± 0.1 | 0.3 ± 0.1 |  |
| 20:4n-6 ** | | 28.2 ± 1.1 | 28.7 ± 2.0 | 22.1 ± 1.3 | 24.2 ± 1.0 |  |
| 20:3n-3 | | N.D. | N.D. | N.D. | N.D. |  |
| 20:5n-3 *** ^♦^ | | N.D. | N.D. | 1.3 ± 0.1 | 1.0 ± 0.1 |  |
| 22:0 | | N.D. | N.D. | 0.1 ± 0.1 | 0.3 ± 0.1 |  |
| 22:1n-9 | | N.D. | N.D. | N.D. | N.D. |  |
| 22:4n-6 | | N.D. | N.D. | N.D. | N.D. |  |
| 22:5n-3 | | N.D. | N.D. | N.D. | N.D. |  |
| 22:6n-3 | | 0.1 ± 0.1 | 0.2 ± 0.2 | 0.4 ± 0.4 | 0.3 ± 0.3 |  |
| 24:0 | | N.D. | N.D. | N.D. | N.D. |  |
| 24:1n-9 | | N.D. | N.D. | N.D. | N.D. |  |
| Other minor FA | | 0.8 ± 0.2 | 1.6 ± 0.4 | 0.7 ± 0.3 | 1.0 ± 0.2 |  |
|  | |  |  |  |  |  |
| Total SAT | | 60.5 ± 2.0 | 58.2 ± 0.4 | 61.9 ± 1.1 | 58.7 ± 1.3 |  |
| Total MUFA | | 8.7 ± 1.5 | 10.1 ± 1.8 | 11.9 ± 1.0 | 12.7 ± 1.5 |  |
| Total PUFA * | | 30.0 ± 0.7 | 30.1 ± 1.7 | 25.4 ± 0.7 | 27.5 ± 1.4 |  |
| Total n-3 FA * | | 0.8 ± 0.4 | 0.6 ± 0.3 | 2.2 ± 0.7 | 2.1 ± 0.4 |  |
| Total n-6 FA ** | | 29.1 ± 1.0 | 29.5 ± 2.0 | 23.2 ± 1.3 | 25.5 ± 1.0 |  |

SFA, saturated fatty acids; MUFA, monounsaturated fatty acids; PUFA, polyunsaturated fatty acids; N.D., not detected; tr., trace (less than 0.05), significant effect of diet, * p < 0.05, ** p < 0.01, *** p < 0.001; significant effect of genotype, ^♦^ p < 0.05.

**Tab1e 23.** Fatty acid composition of phosphatidylinositol (PI) from cerebellum of 12 month-old wild-type (WT) and transgenic (Tg) mice on the oil blend diet (OB) or the DHA diet. Results are represented as mean percentage of total fatty acids ± SEM. Analysis by GLC.

|  | PI Cerebellum - 12 months | | | | | |
| --- | --- | --- | --- | --- | --- | --- |
| Fatty acid | | Tg OB (n = 4) | WT OB (n = 4) | Tg DHA (n = 4) | WT DHA (n = 4) |  |
| 12:0 | | N.D. | N.D. | N.D. | 0.1 ± tr. |  |
| 16:0 | | 7.3 ± 0.9 | 6.1 ± 0.6 | 6.5 ± 1.0 | 8.7 ± 1.3 |  |
| 16:1n-7 | | 0.8 ± 0.2 | 0.6 ± 0.3 | 0.6 ± 0.2 | 1.4 ± 0.4 |  |
| 18:0 ** | | 40.6 ± 2.5 | 36.2 ± 0.6 | 34.4 ± 0.3 | 33.2 ± 1.2 |  |
| 18:1n-9 ** | | 7.4 ± 0.5 | 6.3 ± 0.7 | 8.4 ± 1.0 | 10.0 ± 0.5 |  |
| 18:1n-7 | | 1.9 ± 0.2 | 1.5 ± 0.6 | 1.8 ± 0.3 | 2.4 ± 0.2 |  |
| 18:2n-6 | | 0.8 ± 0.3 | 1.5 ± 0.3 | 1.0 ± 0.3 | 1.2 ± 0.4 |  |
| 18:3n-6 | | 0.2 ± 0.2 | 0.3 ± 0.2 | 0.4 ± 0.2 | 0.6 ± 0.1 |  |
| 18:3n-3 | | 0.7 ± 0.2 | 0.9 ± 0.3 | 1.8 ± 1.1 | 0.7 ± 0.2 |  |
| 20:0 | | 0.7 ± 0.3 | 2.4 ± 0.5 | 1.6 ± 0.9 | 2.0 ± 0.8 |  |
| 20:1n-9 | | 1.0 ± 0.4 | 1.0 ± 0.6 | 0.8 ± 0.3 | 1.2 ± 0.3 |  |
| 20:3n-6 *** | | 0.3 ± 0.2 | 0.3 ± 0.3 | 1.5 ± 0.2 | 1.9 ± 0.4 |  |
| 20:4n-6 ** | | 32.3 ± 2.7 | 31.4 ± 1.7 | 26.8 ± 1.2 | 23.1 ± 0.9 |  |
| 20:3n-3 * | | 0.2 ± 0.1 | 0.1 ± 0.1 | 0.5 ± 0.2 | 0.8 ± 0.3 |  |
| 20:5n-3 * | | 0.5 ± 0.3 | 2.1 ± 0.7 | 2.4 ± 0.5 | 2.3 ± 0.3 |  |
| 22:0 | | 0.1 ± 0.1 | 0.6 ± 0.4 | 1.2 ± 0.6 | 0.3 ± 0.3 |  |
| 22:1n-9 | | 0.1 ± 0.1 | 0.4 ± 0.4 | 0.2 ± 0.1 | N.D. |  |
| 22:4n-6 | | N.D. | N.D. | N.D. | N.D. |  |
| 22:5n-3 | | N.D. | N.D. | N.D. | N.D. |  |
| 22:6n-3 *** | | 3.4 ± 0.4 | 3.6 ± 0.7 | 7.2 ± 0.4 | 6.7 ± 0.2 |  |
| 24:0 | | N.D. | N.D. | N.D. | N.D. |  |
| 24:1n-9 | | N.D. | N.D. | N.D. | N.D. |  |
| Other minor FA | | 1.8 ± 0.3 | 4.7 ± 1.6 | 2.8 ± 0.9 | 3.6 ± 0.4 |  |
|  | |  |  |  |  |  |
| Total SAT | | 48.7 ± 3.2 | 45.3 ± 1.3 | 43.7 ± 0.7 | 44.2 ± 1.5 |  |
| Total MUFA | | 11.0 ± 1.2 | 9.8 ± 2.0 | 11.9 ± 1.9 | 15.0 ± 0.9 |  |
| Total PUFA | | 38.5 ± 3.3 | 40.2 ± 1.9 | 41.6 ± 1.8 | 37.3 ± 2.0 |  |
| Total n-3 FA *** | | 4.7 ± 0.7 | 6.7 ± 0.4 | 12.0 ± 1.3 | 10.4 ± 0.9 |  |
| Total n-6 FA ** | | 33.7 ± 2.8 | 33.5 ± 1.5 | 29.7 ± 0.6 | 26.8 ± 1.1 |  |

SFA, saturated fatty acids; MUFA, monounsaturated fatty acids; PUFA, polyunsaturated fatty acids; N.D., not detected; tr., trace (less than 0.05); significant effect of diet, * p < 0.05, ** p < 0.01, *** p < 0.001.

**Tab1e 24.** Fatty acid composition of phosphatidylinositol (PI) from cerebellum of 16 month-old wild-type (WT) and transgenic (Tg) mice on the oil blend diet (OB) or the DHA diet. Results are represented as mean percentage of total fatty acids ± SEM. Analysis by GLC.

|  | PI Cerebellum - 16 months | | | | | |
| --- | --- | --- | --- | --- | --- | --- |
| Fatty acid | | Tg OB (n = 3) | WT OB (n = 3) | Tg DHA (n = 3) | WT DHA (n = 3) |  |
| 12:0 | | N.D. | N.D. | N.D. | N.D. |  |
| 16:0 | | 11.3 ± 1.4 | 10.6 ± 2.0 | 11.1 ± 0.5 | 10.1 ± 0.2 |  |
| 16:1n-7 | | 2.2 ± 0.7 | 2.3 ± 1.4 | 1.0 ± tr. | 0.8 ± 0.1 |  |
| 18:0 | | 44.2 ± 1.1 | 43.6 ± 0.1 | 41.6 ± 0.8 | 43.0 ± 1.6 |  |
| 18:1n-9 | | 10.1 ± 1.6 | 9.8 ± 1.5 | 12.1 ± 0.9 | 11.7 ± 0.9 |  |
| 18:1n-7 | | 1.8 ± 0.2 | 1.8 ± 0.2 | 2.3 ± 0.3 | 2.3 ± 0.2 |  |
| 18:2n-6 | | 1.1 ± 0.4 | 1.2 ± 0.5 | 0.9 ± 0.1 | 0.6 ± 0.3 |  |
| 18:3n-6 | | N.D. | N.D. | N.D. | N.D. |  |
| 18:3n-3 | | 0.9 ± 0.3 | 0.8 ± 0.4 | 0.5 ± 0.1 | 0.5 ± tr. |  |
| 20:0 | | 0.2 ± 0.2 | 0.1 ± 0.1 | 0.2 ± 0.1 | 0.1 ± 0.1 |  |
| 20:1n-9 ** | | 0.1 ± 0.1 | 0.1 ± 0.1 | 0.5 ± 0.1 | 0.6 ± 0.1 |  |
| 20:3n-6 *** | | 0.1 ± 0.1 | 0.1 ± 0.1 | 0.9 ± tr. | 1.0 ± tr. |  |
| 20:4n-6 | | 25.0 ± 3.6 | 27.3 ± 4.4 | 19.6 ± 1.8 | 21.5 ± 1.2 |  |
| 20:3n-3 | | N.D. | N.D. | N.D. | N.D. |  |
| 20:5n-3 *** | | N.D. | N.D. | 2.1 ± 0.1 | 1.8 ± 0.1 |  |
| 22:0 | | N.D. | N.D. | N.D. | N.D. |  |
| 22:1n-9 | | N.D. | N.D. | N.D. | N.D. |  |
| 22:4n-6 | | N.D. | N.D. | N.D. | N.D. |  |
| 22:5n-3 | | N.D. | N.D. | N.D. | N.D. |  |
| 22:6n-3 *** | | 2.4 ± 0.4 | 2.2 ± 1.1 | 6.9 ± 0.2 | 5.8 ± 0.4 |  |
| 24:0 | | N.D. | N.D. | N.D. | N.D. |  |
| 24:1n-9 | | N.D. | N.D. | N.D. | N.D. |  |
| Other minor FA | | 0.5 ± 0.5 | 0.1 ± 0.1 | 0.3 ± 0.2 | 0.2 ± 0.2 |  |
|  | |  |  |  |  |  |
| Total SAT | | 55.8 ± 2.0 | 54.3 ± 2.1 | 52.8 ± 1.3 | 53.3 ± 1.5 |  |
| Total MUFA | | 14.2 ± 1.6 | 14.0 ± 2.6 | 16.0 ± 0.9 | 15.4 ± 0.8 |  |
| Total PUFA | | 29.4 ± 3.3 | 31.5 ± 4.6 | 30.9 ± 1.5 | 31.1 ± 1.6 |  |
| Total n-3 FA *** | | 3.2 ± 0.1 | 3.0 ± 0.7 | 9.5 ± 0.2 | 8.0 ± 0.4 |  |
| Total n-6 FA | | 26.2 ± 3.3 | 28.6 ± 3.9 | 21.5 ± 1.7 | 23.1 ± 1.2 |  |

SFA, saturated fatty acids; MUFA, monounsaturated fatty acids; PUFA, polyunsaturated fatty acids; N.D., not detected; tr., trace (less than 0.05); significant effect of diet, ** p < 0.01, *** p < 0.001.

**Tab1e 25.** Fatty acid composition of sphingomyelin (Sph) from cortex of 12 month-old wild-type (WT) and transgenic (Tg) mice on the oil blend diet (OB) or the DHA diet. Results are represented as mean percentage of total fatty acids ± SEM. Analysis by GLC.

|  | Sph Cortex - 12 months | | | | | |
| --- | --- | --- | --- | --- | --- | --- |
| Fatty acid | | Tg OB (n = 4) | WT OB (n = 4) | Tg DHA (n = 4) | WT DHA (n = 4) |  |
| 12:0 | | 0.2 ± 0.2 | N.D. | 0.1 ± 0.1 | N.D. |  |
| 16:0 | | 4.5 ± 0.7 | 4.6 ± 0.9 | 4.0 ± 0.7 | 3.9 ± 0.7 |  |
| 16:1n-7 | | 2.8 ± 0.5 | 2.2 ± 0.3 | 2.3 ± 0.4 | 2.5 ± 0.5 |  |
| 18:0 | | 68.5 ± 3.7 | 70.5 ± 1.9 | 60.7 ± 7.7 | 69.6 ± 4.1 |  |
| 18:1n-9 | | 3.8 ± 0.4 | 3.7 ± 0.7 | 3.8 ± 0.6 | 4.1 ± 1.0 |  |
| 18:1n-7 | | N.D. | N.D. | N.D. | N.D. |  |
| 18:2n-6 | | 1.1 ± 0.2 | 0.9 ± 0.2 | 1.1 ± 0.3 | 1.1 ± 0.4 |  |
| 18:3n-6 | | 0.6 ± 0.6 | 0.1 ± 0.1 | 0.1 ± 0.1 | 0.5 ± 0.2 |  |
| 18:3n-3 | | 0.5 ± tr. | 0.3 ± 0.1 | 0.5 ± 0.1 | 0.3 ± 0.1 |  |
| 20:0 | | 2.0 ± 0.2 | 2.0 ± 0.1 | 2.0 ± 0.3 | 2.3 ± 0.1 |  |
| 20:1n-9 | | 0.3 ± 0.2 | 0.1 ± 0.1 | 1.6 ± 1.3 | 0.1 ± 0.1 |  |
| 20:3n-6 | | N.D. | N.D. | N.D. | N.D. |  |
| 20:4n-6 | | 0.2 ± 0.2 | 0.1 ± 0.1 | 0.1 ± 0.1 | 0.1 ± 0.1 |  |
| 20:3n-3 | | 1.4 ± 0.9 | 1.8 ± 0.5 | 3.6 ± 1.9 | 1.7 ± 0.6 |  |
| 20:5n-3 | | 0.1 ± 0.1 | N.D. | 0.2 ± 0.2 | 0.1 ± 0.1 |  |
| 22:0 | | 2.1 ± 0.4 | 2.3 ± 0.1 | 3.8 ± 1.0 | 2.4 ± 0.4 |  |
| 22:1n-9 | | 0.5 ± 0.3 | 1.2 ± 0.9 | 2.3 ± 1.2 | 1.2 ± 0.7 |  |
| 22:4n-6 | | N.D. | N.D. | N.D. | 0.6 ± 0.6 |  |
| 22:5n-3 | | N.D. | N.D. | N.D. | N.D. |  |
| 22:6n-3 | | 0.1 ± 0.1 | 0.2 ± 0.2 | 0.2 ± 0.2 | N.D. |  |
| 24:0 ^♦^ | | 2.4 ± 0.4 | 1.4 ± 0.3 | 3.6 ± 0.9 | 1.6 ± 0.7 |  |
| 24:1n-9 | | 5.4 ± 1.4 | 5.5 ± 1.4 | 6.0 ± 1.3 | 5.0 ± 1.2 |  |
| Other minor FA | | 3.4 ± 0.9 | 3.0 ± 0.2 | 4.2 ± 1.2 | 2.8 ± 0.6 |  |
|  | |  |  |  |  |  |
| Total SAT | | 79.7 ± 3.2 | 80.9 ± 2.4 | 74.2 ± 5.9 | 79.9 ± 4.1 |  |
| Total MUFA | | 12.7 ± 2.2 | 12.7 ± 1.8 | 16.0 ± 3.4 | 12.9 ± 3.2 |  |
| Total PUFA | | 4.2 ± 1.2 | 3.4 ± 0.6 | 5.6 ± 1.8 | 4.4 ± 1.1 |  |
| Total n-3 FA | | 2.2 ± 1.0 | 2.3 ± 0.5 | 4.4 ± 1.8 | 2.0 ± 0.6 |  |
| Total n-6 FA | | 2.0 ± 0.7 | 1.1 ± 0.2 | 1.2± 0.3 | 2.3 ± 0.7 |  |

SFA, saturated fatty acids; MUFA, monounsaturated fatty acids; PUFA, polyunsaturated fatty acids; N.D., not detected; tr., trace (less than 0.05); significant effect of genotype, ^♦^ p < 0.05.

**Tab1e 26.** Fatty acid composition of sphingomyelin (Sph) from cortex of 16 month-old wild-type (WT) and transgenic (Tg) mice on the oil blend diet (OB) or the DHA diet. Results are represented as mean percentage of total fatty acids ± SEM. Analysis by GLC.

|  | Sph Cortex - 16 months | | | | | |
| --- | --- | --- | --- | --- | --- | --- |
| Fatty acid | | Tg OB (n = 3) | WT OB (n = 3) | Tg DHA (n = 3) | WT DHA (n = 3) |  |
| 12:0 | | N.D. | N.D. | N.D. | N.D. |  |
| 16:0 | | 6.9 ± 0.8 | 7.5 ± 1.0 | 6.8 ± 0.9 | 6.7 ± 1.0 |  |
| 16:1n-7 | | 0.5 ± 0.5 | 0.6 ± 0.6 | 0.8 ± 0.8 | 0.8 ± 0.4 |  |
| 18:0 | | 85.6 ± 1.0 | 84.2 ± 1.9 | 84.2 ± 2.2 | 85.0 ± 1.2 |  |
| 18:1n-9 | | 3.7 ± 0.5 | 3.9 ± 0.6 | 4.1 ± 0.7 | 3.9 ± 0.5 |  |
| 18:1n-7 | | N.D. | N.D. | N.D. | N.D. |  |
| 18:2n-6 | | 0.3 ± 0.1 | 0.4 ± tr. | 0.5 ± tr. | 0.4 ± 0.1 |  |
| 18:3n-6 | | N.D. | N.D. | N.D. | N.D. |  |
| 18:3n-3 | | 0.2 ± 0.2 | N.D. | N.D. | N.D. |  |
| 20:0 | | 1.5 ± tr. | 1.6 ± 0.1 | 1.6 ± tr. | 1.6 ± 0.1 |  |
| 20:1n-9 | | N.D. | N.D. | N.D. | N.D. |  |
| 20:3n-6 | | N.D. | N.D. | N.D. | N.D. |  |
| 20:4n-6 | | N.D. | N.D. | N.D. | N.D. |  |
| 20:3n-3 | | N.D. | N.D. | N.D. | N.D. |  |
| 20:5n-3 | | N.D. | N.D. | N.D. | N.D. |  |
| 22:0 | | 0.3 ± 0.3 | 0.6 ± 0.3 | 0.7 ± 0.4 | 0.5 ± 0.3 |  |
| 22:1n-9 | | N.D. | N.D. | N.D. | N.D. |  |
| 22:4n-6 | | N.D. | N.D. | N.D. | N.D. |  |
| 22:5n-3 | | N.D. | N.D. | N.D. | N.D. |  |
| 22:6n-3 | | N.D. | N.D. | N.D. | N.D. |  |
| 24:0 | | N.D. | N.D. | N.D. | N.D. |  |
| 24:1n-9 | | N.D. | N.D. | 0.2 ± 0.2 | N.D. |  |
| Other minor FA | | 1.0 ± 0.3 | 1.2 ± 0.3 | 1.2 ± 0.2 | 1.2 ± 0.1 |  |
|  | |  |  |  |  |  |
| Total SAT | | 94.3 ± 0.4 | 93.9 ± 1.2 | 93.3 ± 1.7 | 93.8 ± 0.5 |  |
| Total MUFA | | 4.2 ± 0.8 | 4.5 ± 1.0 | 5.1 ± 1.4 | 4.7 ± 0.4 |  |
| Total PUFA | | 0.5 ± 0.3 | 0.4 ± tr. | 0.5 ± tr. | 0.4 ± 0.1 |  |
| Total n-3 FA | | 0.2 ± 0.2 | N.D. | N.D. | N.D. |  |
| Total n-6 FA | | 0.3 ± 0.1 | 0.4 ± tr. | 0.5 ± tr. | 0.4 ± 0.1 |  |

SFA, saturated fatty acids; MUFA, monounsaturated fatty acids; PUFA, polyunsaturated fatty acids; N.D., not detected; tr., trace (less than 0.05).

**Tab1e 27.** Fatty acid composition of sphingomyelin (Sph) from hippocampus of 12 month-old wild-type (WT) and transgenic (Tg) mice on the oil blend diet (OB) or the DHA diet. Results are represented as mean percentage of total fatty acids ± SEM. Analysis by GLC.

|  | Sph Hippocampus - 12 months | | | | | |
| --- | --- | --- | --- | --- | --- | --- |
| Fatty acid | | Tg OB (n = 4) | WT OB (n = 4) | Tg DHA (n = 4) | WT DHA (n = 4) |  |
| 12:0 | | 0.1 ± 0.1 | 0.1 ± tr. | 0.2 ± 0.2 | 0.4 ± 0.3 |  |
| 16:0 | | 7.6 ±1.3 | 7.3 ± 0.6 | 8.7 ± 2.2 | 4.4 ± 0.9 |  |
| 16:1n-7 | | 1.6 ± 0.2 | 1.6 ± 0.1 | 1.9 ± 0.1 | 1.7 ± 0.3 |  |
| 18:0 | | 71.5 ± 1.0 | 75.4 ± 1.8 | 70.8 ± 2.6 | 74.9 ± 0.7 |  |
| 18:1n-9 | | 9.9 ± 0.3 | 6.8 ± 2.2 | 9.3 ± 0.7 | 8.1 ± 1.8 |  |
| 18:1n-7 | | N.D. | N.D. | N.D. | N.D. |  |
| 18:2n-6 | | 0.7 ± tr. | 0.6 ± tr. | 1.0 ± 0.2 | 0.5 ± 0.2 |  |
| 18:3n-6 | | 0.1 ± 0.1 | N.D. | 0.2 ± 0.1 | N.D. |  |
| 18:3n-3 | | 0.1 ± 0.1 | 0.2 ± 0.1 | 0.2 ± 0.1 | 0.1 ± 0.1 |  |
| 20:0 | | 1.3 ± 0.1 | 1.3 ± 0.1 | 1.2 ± 0.1 | 1.6 ± 0.3 |  |
| 20:1n-9 | | 0.3 ± 0.3 | 0.5 ± 0.3 | 0.6 ± 0.4 | 0.2 ± 0.2 |  |
| 20:3n-6 | | N.D. | N.D. | N.D. | N.D. |  |
| 20:4n-6 | | N.D. | N.D. | N.D. | N.D. |  |
| 20:3n-3 | | 0.8 ± 0.5 | 0.8 ± 0.1 | 0.8 ± 0.2 | 1.3 ± 0.3 |  |
| 20:5n-3 | | 0.2 ± 0.2 | N.D. | 0.1 ± 0.1 | N.D. |  |
| 22:0 | | 1.3 ± 0.2 | 1.0 ± 0.4 | 0.8 ± 0.4 | 1.4 ± 0.5 |  |
| 22:1n-9 | | N.D. | N.D. | N.D. | N.D. |  |
| 22:4n-6 | | 0.4 ± 0.3 | 0.4 ± 0.3 | 0.5 ± 0.4 | 1.4 ± 1.0 |  |
| 22:5n-3 | | N.D. | N.D. | N.D. | N.D. |  |
| 22:6n-3 | | N.D. | N.D. | N.D. | N.D. |  |
| 24:0 | | 0.4 ± 0.1 | 0.2 ± 02. | 0.3 ± 0.1 | 0.7 ± 0.5 |  |
| 24:1n-9 | | 1.8 ± 0.7 | 2.2 ± 0.8 | 2.0 ± 0.8 | 2.5 ± 1.1 |  |
| Other minor FA | | 1.9 ± 0.7 | 1.5 ± 0.5 | 1.4 ± 0.5 | 0.8 ± 0.1 |  |
|  | |  |  |  |  |  |
| Total SAT | | 82.2 ± 0.9 | 85.3 ± 1.9 | 82.1 ± 0.9 | 83.4 ± 0.9 |  |
| Total MUFA ^♦^ | | 13.6 ± 1.0 | 11.2 ± 1.8 | 13.8 ± 0.5 | 12.6 ± 1.7 |  |
| Total PUFA | | 2.3 ± 0.6 | 2.0 ± 0.2 | 2.7 ± 0.5 | 3.3 ± 1.4 |  |
| Total n-3 FA | | 1.1 ± 0.4 | 1.0 ± 0.2 | 1.1 ± 0.3 | 1.4 ± 0.4 |  |
| Total n-6 FA | | 1.2 ± 0.5 | 1.1 ± 0.3 | 1.6 ± 0.2 | 1.9 ± 1.1 |  |

SFA, saturated fatty acids; MUFA, monounsaturated fatty acids; PUFA, polyunsaturated fatty acids; N.D., not detected; tr., trace (less than 0.05); significant effect of genotype, ^♦^ p < 0.05.

**Tab1e 28.** Fatty acid composition of sphingomyelin (Sph) from hippocampus of 16 month-old wild-type (WT) and transgenic (Tg) mice on the oil blend diet (OB) or the DHA diet. Results are represented as mean percentage of total fatty acids ± SEM. Analysis by GLC.

|  | Sph Hippocampus - 16 months | | | | | |
| --- | --- | --- | --- | --- | --- | --- |
| Fatty acid | | Tg OB (n = 3) | WT OB (n = 3) | Tg DHA (n = 3) | WT DHA (n = 3) |  |
| 12:0 | | N.D. | N.D. | 0.1 ± 0.1 | N.D. |  |
| 16:0 | | 10.7 ± 1.2 | 11.3 ± 2.6 | 10.5 ± 2.6 | 9.1 ± 0.8 |  |
| 16:1n-7 | | 4.0 ± 1.1 | 2.6 ± 0.5 | 3.3 ± 0.7 | 3.0 ± 1.2 |  |
| 18:0 | | 75.3 ± 1.4 | 74.9 ± 5.0 | 74.7 ± 5.1 | 78.7 ± 1.9 |  |
| 18:1n-9 | | 5.6 ± 0.6 | 5.8 ± 0.8 | 6.3 ± 0.7 | 6.4 ± 0.2 |  |
| 18:1n-7 | | N.D. | 0.1 ± 0.1 | 0.1 ± 0.1 | N.D. |  |
| 18:2n-6 | | 0.8 ± 0.1 | 0.6 ± 0.1 | 0.6 ± 0.1 | 0.7 ± tr. |  |
| 18:3n-6 | | N.D. | N.D. | N.D. | N.D. |  |
| 18:3n-3 | | 0.6 ± 0.3 | 0.6 ± 0.3 | 0.5 ± 0.2 | 0.4 ± 0.2 |  |
| 20:0 | | 1.1 ± 0.1 | 1.1 ± 0.1 | 1.1 ± tr. | 0.7 ± 0.4 |  |
| 20:1n-9 | | N.D. | N.D. | N.D. | N.D. |  |
| 20:3n-6 | | 0.4 ± 0.2 | 0.8 ± 0.2 | 0.8 ± 0.1 | 0.5 ± 0.2 |  |
| 20:4n-6 | | N.D. | N.D. | N.D. | N.D. |  |
| 20:3n-3 | | N.D. | 0.1 ± 0.1 | N.D. | N.D. |  |
| 20:5n-3 | | N.D. | N.D. | N.D. | N.D. |  |
| 22:0 | | 0.1 ± 0.1 | 0.2 ± 0.1 | 0.2 ± 0.1 | N.D. |  |
| 22:1n-9 | | 0.3 ± 0.2 | 0.4 ± 0.2 | 0.4 ± 0.2 | 0.2 ± 0.2 |  |
| 22:4n-6 | | N.D. | N.D. | N.D. | N.D. |  |
| 22:5n-3 | | N.D. | N.D. | N.D. | N.D. |  |
| 22:6n-3 | | N.D. | N.D. | N.D. | N.D. |  |
| 24:0 | | N.D. | N.D. | N.D. | N.D. |  |
| 24:1n-9 | | N.D. | N.D. | N.D. | N.D. |  |
| Other minor FA | | 1.1 ± 0.4 | 1.4 ± 1.0 | 1.3 ± 1.1 | 0.4 ± 0.2 |  |
|  | |  |  |  |  |  |
| Total SAT | | 87.1 ± 1.8 | 87.6 ± 2.5 | 86.6 ± 2.6 | 88.5 ± 1.6 |  |
| Total MUFA | | 10.0 ± 1.5 | 9.0 ± 1.5 | 10.1 ± 1.5 | 9.5 ± 1.0 |  |
| Total PUFA | | 1.8 ± 0.6 | 2.1 ± 0.3 | 1.9 ± 0.2 | 1.6 ± 0.5 |  |
| Total n-3 FA | | 0.6 ± 0.3 | 0.7 ± 0.4 | 0.5 ± 0.2 | 0.4 ± 0.2 |  |
| Total n-6 FA | | 1.2 ± 0.3 | 1.4 ± 0.1 | 1.4 ± 0.1 | 1.2 ± 0.3 |  |

SFA, saturated fatty acids; MUFA, monounsaturated fatty acids; PUFA, polyunsaturated fatty acids; N.D., not detected; tr., trace (less than 0.05).

**Tab1e 29.** Fatty acid composition of sphingomyelin (Sph) from cerebellum of 12 month-old wild-type (WT) and transgenic (Tg) mice on the oil blend diet (OB) or the DHA diet. Results are represented as mean percentage of total fatty acids ± SEM. Analysis by GLC.

|  | Sph Cerebellum - 12 months | | | | | |
| --- | --- | --- | --- | --- | --- | --- |
| Fatty acid | | Tg OB (n = 4) | WT OB (n = 4) | Tg DHA (n = 4) | WT DHA (n = 4) |  |
| 12:0 | | N.D. | N.D. | 0.1 ± 0.1 | 0.4 ± 0.4 |  |
| 16:0 | | 10.3 ± 1.0 | 9.7 ± 1.5 | 9.7 ± 2.1 | 8.5 ± 1.3 |  |
| 16:1n-7 | | 1.5 ± 0.4 | 1.9 ± 0.2 | 1.7 ± 0.6 | 1.9 ± 0.3 |  |
| 18:0 | | 55.5 ± 4.4 | 54.4 ± 3.9 | 49.8 ± 5.9 | 58.8 ± 2.9 |  |
| 18:1n-9 | | 5.2 ± 1.4 | 4.0 ± 0.1 | 3.2 ± 1.4 | 6.3 ± 0.8 |  |
| 18:1n-7 | | 1.0 ± 0.7 | 1.3 ± 1.1 | 2.0 ± 1.0 | 0.6 ± 0.6 |  |
| 18:2n-6 | | 1.6 ± 0.3 | 2.2 ± 0.6 | 1.3 ± 0.5 | 1.8 ± 0.4 |  |
| 18:3n-6 | | 1.0 ± 0.6 | 0.9 ± 0.6 | 0.8 ± 0.4 | 1.1 ± 0.4 |  |
| 18:3n-3 | | 2.7 ± 0.8 | 2.3 ± 0.3 | 2.8 ± 0.4 | 1.1 ± 0.4 |  |
| 20:0 | | 3.3 ± 0.7 | 5.3 ± 1.5 | 4.0 ± 2.0 | 3.8 ± 0.5 |  |
| 20:1n-9 | | 1.4 ± 0.7 | 1.5 ± 0.8 | 1.0 ± 0.6 | 2.2 ± 0.8 |  |
| 20:3n-6 | | 1.2 ± 0.5 | 1.6 ± 0.5 | 1.3 ± 0.5 | 1.0 ± 0.4 |  |
| 20:4n-6 | | 0.3 ± 0.2 | 0.2 ± 0.2 | 3.1 ± 2.5 | 1.3 ± 0.6 |  |
| 20:3n-3 | | 1.8 ± 0.6 | 2.2 ± 0.9 | 1.5 ± 0.6 | 0.8 ± 0.3 |  |
| 20:5n-3 | | 1.4 ± 0.3 | 1.4 ± 0.5 | 1.1 ± 0.1 | 0.8 ± 0.5 |  |
| 22:0 | | 0.5 ± 0.3 | 0.7 ± 0.2 | 2.8 ± 1.3 | 1.5 ± 0.8 |  |
| 22:1n-9 | | 1.6 ± 0.9 | 0.6 ± 0.4 | 1.3 ± 0.9 | 0.4 ± 0.4 |  |
| 22:4n-6 | | N.D. | 0.3 ± 0.2 | 1.0 ± 1.0 | 0.4 ± 0.3 |  |
| 22:5n-3 | | N.D. | N.D. | N.D. | N.D. |  |
| 22:6n-3 | | N.D. | N.D. | 0.4 ± 0.4 | N.D. |  |
| 24:0 | | N.D. | 0.1 ± 0.1 | N.D. | 0.8 ± 0.8 |  |
| 24:1n-9 | | 2.8 ± 1.7 | 0.8 ± 0.8 | 5.7 ± 3.6 | 1.8 ± 1.8 |  |
| Other minor FA | | 6.9 ± 0.6 | 8.9 ± 1.2 | 5.7 ± 1.6 | 5.1 ± 1.5 |  |
|  | |  |  |  |  |  |
| Total SAT | | 69.6 ± 3.1 | 70.2 ± 3.1 | 66.4 ± 4.3 | 73.7 ± 2.6 |  |
| Total MUFA | | 13.6 ± 2.0 | 9.9 ± 0.5 | 14.9 ± 1.9 | 13.0 ± 1.2 |  |
| Total PUFA | | 10.0 ± 1.2 | 11.0 ± 2.4 | 13.1 ± 3.4 | 8.2 ± 2.0 |  |
| Total n-3 FA | | 5.8 ± 0.6 | 5.9 ± 1.6 | 5.7 ± 0.5 | 2.6 ± 1.1 |  |
| Total n-6 FA | | 4.1 ± 1.3 | 5.1 ± 1.5 | 7.3 ± 3.0 | 5.6 ± 1.1 |  |

SFA, saturated fatty acids; MUFA, monounsaturated fatty acids; PUFA, polyunsaturated fatty acids; N.D., not detected; tr., trace (less than 0.05).

**Tab1e 30.** Fatty acid composition of sphingomyelin (Sph) from cerebellum of 16 month-old wild-type (WT) and transgenic (Tg) mice on the oil blend diet (OB) or the DHA diet. Results are represented as mean percentage of total fatty acids ± SEM. Analysis by GLC.

|  | Sph Cerebellum - 16 months | | | | | |
| --- | --- | --- | --- | --- | --- | --- |
| Fatty acid | | Tg OB (n = 3) | WT OB (n = 3) | Tg DHA (n = 3) | WT DHA (n = 3) |  |
| 12:0 | | N.D. | N.D. | 0.1 ± 0.1 | N.D. |  |
| 16:0 | | 17.1 ± 1.9 | 14.7 ± 1.6 | 14.3 ± 1.4 | 12.4 ± 0.9 |  |
| 16:1n-7 | | 3.6 ± 0.8 | 3.3 ± 1.0 | 2.4 ± 0.5 | 3.0 ± 0.8 |  |
| 18:0 | | 59.9 ± 3.1 | 64.7 ± 3.1 | 64.8 ± 1.0 | 69.1 ± 2.5 |  |
| 18:1n-9 | | 11.1 ± 2.5 | 8.6 ± 2.6 | 6.9 ± 0.9 | 6.5 ± 0.2 |  |
| 18:1n-7 | | N.D. | N.D. | N.D. | N.D. |  |
| 18:2n-6 | | 1.2 ± 0.4 | 0.9 ± 0.2 | 0.9 ± 0.2 | 0.7 ± tr. |  |
| 18:3n-6 | | N.D. | N.D. | N.D. | N.D. |  |
| 18:3n-3 | | 1.3 ± 0.3 | 1.2 ± 0.2 | 1.0 ± tr. | 0.7 ± 0.1 |  |
| 20:0 | | 1.4 ± tr. | 1.3 ± 0.1 | 1.5 ± 0.1 | 1.4 ± tr. |  |
| 20:1n-9 | | N.D. | N.D. | N.D. | N.D. |  |
| 20:3n-6 | | N.D. | 0.4 ± 0.4 | 0.3 ± 0.3 | 0.4 ± 0.4 |  |
| 20:4n-6 | | 0.6 ± 0.6 | N.D. | N.D. | 0.6 ± 0.6 |  |
| 20:3n-3 | | N.D. | N.D. | N.D. | 0.1 ± 0.1 |  |
| 20:5n-3 | | 0.4 ± 0.4 | 0.1 ± 0.1 | 0.2 ± 0.2 | 0.2 ± 0.2 |  |
| 22:0 | | 0.9 ± 0.5 | 0.8 ± 0.4 | 1.4 ± 0.5 | 1.5 ± 0.1 |  |
| 22:1n-9 | | N.D. | N.D. | 0.5 ± 0.5 | N.D. |  |
| 22:4n-6 | | 0.1 ± 0.1 | 0.1 ± 0.1 | 0.3 ± 0.1 | 0.1 ± 0.1 |  |
| 22:5n-3 | | N.D. | N.D. | N.D. | N.D. |  |
| 22:6n-3 | | N.D. | N.D. | N.D. | N.D. |  |
| 24:0 | | 0.3 ± 0.3 | 0.5 ± 0.2 | 1.1 ± 0.4 | 0.5 ± 0.3 |  |
| 24:1n-9 | | 1.3 ± 0.7 | 2.3 ± 1.2 | 3.4 ± 0.4 | 2.2 ± 0.3 |  |
| Other minor FA | | 0.8 ± 0.7 | 1.0 ± 0.5 | 1.2 ± 0.5 | 0.6 ± 0.3 |  |
|  | |  |  |  |  |  |
| Total SAT | | 79.6 ± 2.1 | 82.1 ± 2.1 | 83.2 ± 0.8 | 84.8 ± 1.8 |  |
| Total MUFA | | 16.0 ± 2.5 | 14.1 ± 2.3 | 13.1 ± 1.1 | 11.7 ± 1.1 |  |
| Total PUFA | | 3.5 ± 0.8 | 2.8 ± 0.3 | 2.6 ± 0.4 | 2.9 ± 0.8 |  |
| Total n-3 FA * | | 1.6 ± 0.2 | 1.3 ± 0.2 | 1.2 ± 0.2 | 0.9 ± 0.1 |  |
| Total n-6 FA | | 1.9 ± 0.6 | 1.5 ± 0.3 | 1.4 ± 0.3 | 1.9 ± 0.7 |  |

SFA, saturated fatty acids; MUFA, monounsaturated fatty acids; PUFA, polyunsaturated fatty acids; N.D., not detected; tr., trace (less than 0.05); significant effect of diet, * p < 0.05.

**Table 31**. Phosphatidylethanolamine (PE) molecular species composition of the cortex of wild-type (WT) and transgenic (Tg) mice on the oil blend diet (OB) or on the DHA diet, at 12 months of age. Results are represented as mean percentages of total molecular species analysed ± SEM. Analysis by LC-MS/MS.

|  | PE Cortex - 12 months | | | | | |
| --- | --- | --- | --- | --- | --- | --- |
| Molecular species | | Tg OB (n = 6) | WT OB (n = 6) | Tg DHA (n = 6) | WT DHA (n = 6) |  |
| 16:0/18:1 *** | | 2.6 ± 0.1 | 2.7 ± 0.1 | 3.0 ± 0.1 | 3.1 ± 0.1 |  |
| 16:0p/18:1 * | | 4.1 ± 0.1 | 3.9 ± 0.1 | 4.5 ± 0.3 | 4.5 ± 0.1 |  |
| 18:0/18:0 *** | | 2.2 ± 0.1 | 2.1 ± 0.1 | 2.9 ± 0.1 | 2.7 ± 0.1 |  |
| 18:0/18:1 | | 6.1 ± 0.2 | 5.7 ± 0.2 | 6.1 ± 0.3 | 5.9 ± 0.2 |  |
| 18:0p/18:1*** | | 7.4 ± 0.2 | 7.5 ± 0.2 | 8.5 ± 0.4 | 8.4 ± 0.2 |  |
| 18:1/18:1 *** | | 5.8 ± 0.1 | 5.7 ± 0.2 | 7.9 ± 0.3 | 8.6 ± 0.2 |  |
| 18:1p/18:1 *** | | 14.7 ± 0.6 | 15.0 ± 0.5 | 17.6 ± 0.6 | 18.4 ± 0.6 |  |
| 18:0/20:1 *** | | 0.7 ± tr. | 0.7 ± 0.1 | 1.0 ± 0.1 | 1.0 ± tr. |  |
| 18:1p/20:1 ^♦^ | | 4.9 ± 0.3 | 5.4 ± 0.3 | 5.2 ± 0.2 | 5.7 ± 0.2 |  |
| 18:1p/20:2 | | 0.4 ± tr. | 0.4 ± tr. | 0.4 ± tr. | 0.4 ± tr. |  |
| 18:0/20:3 *** | | 0.6 ± tr. | 0.5 ± tr. | 2.8 ± 0.2 | 2.5 ± 0.1 |  |
| 16:0/20:4 *** | | 1.6 ± 0.1 | 1.7 ± 0.1 | 1.0 ± tr. | 1.0 ± 0.1 |  |
| 16:0p/20:4 *** | | 2.4 ± 0.1 | 2.4 ± 0.1 | 1.4 ± 0.1 | 1.3 ± 0.1 |  |
| 18:0/20:4 *** | | 12.1 ± 0.5 | 11.9 ± 0.3 | 8.3 ± 0.3 | 7.7 ± 0.3 |  |
| 18:0p/20:4 *** | | 5.6 ± 0.2 | 6.1 ± 0.2 | 3.1 ± 0.2 | 2.9 ± 0.1 |  |
| 18:0e/20:4 *** | | 0.4 ± tr. | 0.4 ± tr. | 0.2 ± tr. | 0.2 ± tr. |  |
| 18:1/20:4 *** | | 1.5 ± tr. | 1.5 ± 0.1 | 0.8 ± tr. | 0.8 ± tr. |  |
| 16:0p/22:4 *** ^♦^ | | 3.2 ± 0.1 | 2.9 ± 0.1 | 1.2 ± 0.1 | 1.0 ± 0.1 |  |
| 18:0/22:4 *** | | 2.8 ± 0.1 | 2.8 ± 0.1 | 1.1 ± tr. | 0.9 ± 0.1 |  |
| 18:0p/22:4 or ***  18:1e/22:4 | | 4.0 ± 0.1 | 3.9 ± 0.2 | 1.6 ± 0.1 | 1.5 ± 0.1 |  |
| 16:0/22:5 | | 0.5 ± tr. | 0.5 ± tr. | 0.5 ± tr. | 0.5 ± tr. |  |
| 16:0p/22:5 *** | | 0.2 ± tr. | 0.2 ± tr. | 0.4 ± tr. | 0.4 ± tr. |  |
| 18:0p/22:5 *** | | 0.2 ± tr. | 0.2 ± tr. | 0.7 ± 0.1 | 0.7 ± 0.1 |  |
| 16:0/22:6 * | | 1.8 ± 0.1 | 2.0 ± 0.1 | 2.2 ± 0.2 | 2.3 ± 0.1 |  |
| 16:0p/22:6 ** | | 2.1 ± 0.1 | 2.0 ± 0.1 | 2.8 ± 0.1 | 2.5 ± 0.2 |  |
| 18:0/22:6 ** | | 6.3 ± 0.3 | 6.0 ± 0.3 | 7.1 ± 0.1 | 7.4 ± 0.2 |  |
| 18:0p/22:6 *** | | 3.7 ± 0.1 | 4.1 ± 0.2 | 5.0 ± 0.2 | 5.2 ± 0.3 |  |
| 18:1/22:6 | | 0.5 ± tr. | 0.5 ± tr. | 0.6 ± tr. | 0.6 ± tr. |  |
| 18:1p/22:6 or ***  18:2e/22:6 | | 1.4 ± 0.1 | 1.4 ± 0.1 | 2.0 ± 0.1 | 2.0 ± 0.1 |  |

p, plasmenyl (or plasmalogen); e, plasmanyl; tr., trace (less than 0.05); significant effect of diet, * p < 0.05, ** p < 0.01, *** p < 0.001; significant effect of genotype, ^♦^ p < 0.05.

**Table 32**. Phosphatidylethanolamine (PE) molecular species composition of the cortex of wild-type (WT) and transgenic (Tg) mice on the oil blend diet (OB) or on the DHA diet, at 16 months of age. Results are represented as mean percentages of total molecular species analysed ± SEM. Analysis by LC-MS/MS.

|  | PE Cortex - 16 months | | | | | |
| --- | --- | --- | --- | --- | --- | --- |
| Molecular species | | Tg OB (n = 3) | WT OB (n = 3) | Tg DHA (n = 3) | WT DHA (n = 3) |  |
| 16:0/18:1 *** | | 3.3 ± 0.1 | 3.5 ± tr. | 4.2 ± 0.3 | 4.4 ± 0.1 |  |
| 16:0p/18:1 | | 3.4 ± 0.2 | 3.0 ± 0.2 | 4.0 ± 0.4 | 3.4 ± 0.2 |  |
| 18:0/18:0 ** | | 2.0 ± tr. | 2.0 ± 0.1 | 2.7 ± 0.2 | 2.6 ± 0.1 |  |
| 18:0/18:1 ** | | 5.2 ± 0.1 | 5.2 ± 0.2 | 5.6 ± 0.1 | 5.7 ± 0.1 |  |
| 18:0p/18:1 ** | | 4.9 ± 0.2 | 4.5 ± 0.3 | 6.4 ± 0.4 | 5.7 ± 0.3 |  |
| 18:1/18:1 *** | | 6.2 ± tr. | 6.4 ± 0.1 | 9.0 ± 0.5 | 9.2 ± 0.2 |  |
| 18:1p/18:1 * | | 12.6 ± 0.6 | 12.5 ± 1.8 | 14.6 ± 0.8 | 16.2 ± 0.5 |  |
| 18:0/20:1 | | 1.0 ± tr. | 1.1 ± tr. | 1.5 ± tr. | 1.5 ± tr. |  |
| 18:1p/20:1 | | 4.0 ± 0.1 | 4.0 ± 0.7 | 4.2 ± 0.4 | 4.3 ± 0.2 |  |
| 18:1p/20:2 | | 0.4 ± tr. | 0.3 ± 0.1 | 0.5 ± 0.2 | 0.3 ± tr. |  |
| 18:0/20:3 *** ^♦♦^ | | 0.4 ± tr. | 0.3 ± tr. | 2.3 ± 0.1 | 1.7 ± 0.1 |  |
| 16:0/20:4 *** ^♦♦^ | | 1.7 ± tr. | 1.8 ± tr. | 0.8 ± 0.1 | 1.1 ± tr. |  |
| 16:0p/20:4 ** | | 1.7 ± tr. | 1.5 ± 0.2 | 0.8 ± 0.1 | 1.0 ± 0.1 |  |
| 18:0/20:4 *** ^♦^ | | 16.2 ± 0.3 | 16.7 ± 0.4 | 8.8 ± 0.4 | 10.1 ± 0.4 |  |
| 18:0p/20:4 *** | | 5.3 ± 0.1 | 5.5 ± 0.5 | 2.6 ± 0.3 | 2.9 ± 0.1 |  |
| 18:0e/20:4 | | 0.3 ± tr. | 0.3 ± 0.1 | 0.2 ± tr. | 0.1 ± tr. |  |
| 18:1/20:4 *** | | 1.6 ± tr. | 1.5 ± tr. | 0.6 ± tr. | 0.7 ± tr. |  |
| 16:0p/22:4 *** | | 3.3 ± 0.1 | 3.1 ± 0.3 | 0.9 ± 0.1 | 1.1 ± tr. |  |
| 18:0/22:4 *** | | 3.1 ± 0.1 | 2.9 ± 0.1 | 0.9 ± tr. | 1.1 ± 0.1 |  |
| 18:0p/22:4 or ***  18:1e/22:4 | | 3.7 ± 0.1 | 3.6 ± tr. | 1.2 ± 0.1 | 1.3 ± 0.1 |  |
| 16:0/22:5 | | 0.7 ± tr. | 0.7 ± tr. | 0.8 ± 0.1 | 0.8 ± tr. |  |
| 16:0p/22:5 ** | | 0.1 ± tr. | 0.1 ± tr. | 0.3 ± 0.1 | 0.2 ± tr. |  |
| 18:0p/22:5 ** | | 0.1 ± tr. | 0.1 ± tr. | 0.7 ± 0.2 | 0.4 ± tr. |  |
| 16:0/22:6 *** | | 2.3 ± 0.1 | 2.4 ± 0.2 | 3.1 ± 0.2 | 3.1 ± tr. |  |
| 16:0p/22:6 *** | | 2.1 ± tr. | 2.2 ± 0.1 | 3.1 ± tr. | 2.7 ± 0.1 |  |
| 18:0/22:6 ** | | 7.4 ± 0.4 | 7.5 ± 0.9 | 10.3 ± 0.5 | 9.4 ± 0.1 |  |
| 18:0p/22:6 *** | | 4.6 ± 0.2 | 4.8 ± 0.2 | 6.7 ± 0.2 | 5.9 ± 0.1 |  |
| 18:1/22:6 ** | | 0.7 ± tr. | 0.7 ± 0.1 | 1.0 ± tr. | 1.0 ± tr. |  |
| 18:1p/22:6 or ***  18:2e/22:6 | | 1.4 ± tr. | 1.6 ± tr. | 2.1 ± tr. | 2.1 ± 0.1 |  |

p, plasmenyl (or plasmalogen); e, plasmanyl; tr., trace (less than 0.05); significant effect of diet, * p < 0.05, ** p < 0.01, *** p < 0.001; significant effect of genotype, ^♦^ p < 0.05, ^♦♦^ p < 0.01.

**Table 33**. Phosphatidylethanolamine (PE) molecular species composition of the hippocampus of wild-type (WT) and transgenic (Tg) mice on the oil blend diet (OB) or on the DHA diet, at 12 months of age. Results are represented as mean percentages of total molecular species analysed ± SEM. Analysis by LC-MS/MS.

|  | PE Hippocampus - 12 months | | | | | |
| --- | --- | --- | --- | --- | --- | --- |
| Molecular species | | Tg OB (n = 6) | WT OB (n = 6) | Tg DHA (n = 6) | WT DHA (n = 6) |  |
| 16:0/18:1 *** | | 2.7 ± 0.1 | 2.5 ± 0.1 | 3.1 ± 0.2 | 3.3 ± 0.1 |  |
| 16:0p/18:1 | | 4.3 ± 0.2 | 4.3 ± 0.2 | 4.5 ± 0.4 | 4.8 ± 0.2 |  |
| 18:0/18:0 ** | | 2.1 ± tr. | 2.0 ± 0.1 | 2.6 ± 0.1 | 2.4 ± 0.1 |  |
| 18:0/18:1 | | 6.8 ± 0.1 | 6.6 ± 0.2 | 6.7 ± 0.2 | 6.5 ± 0.1 |  |
| 18:0p/18:1 ** | | 6.7 ± 0.1 | 6.7 ± 0.1 | 7.6 ± 0.4 | 7.7 ± 0.3 |  |
| 18:1/18:1 *** ^♦^ | | 4.8 ± 0.1 | 4.9 ± 0.1 | 7.1 ± 0.3 | 8.0 ± 0.3 |  |
| 18:1p/18:1 *** | | 16.1 ± 0.7 | 16.9 ± 0.8 | 19.7 ± 0.8 | 20.6 ± 0.7 |  |
| 18:0/20:1 *** | | 0.6 ± tr. | 0.7 ± tr. | 1.0 ± tr. | 0.9 ± tr. |  |
| 18:1p/20:1 | | 5.2 ± 0.4 | 5.6 ± 0.4 | 5.2 ± 0.4 | 6.1 ± 0.2 |  |
| 18:1p/20:2 | | 0.5 ± tr. | 0.5 ± tr. | 0.5 ± 0.1 | 0.5 ± tr. |  |
| 18:0/20:3 *** ^♦^ | | 0.5 ± tr. | 0.5 ± tr. | 2.0 ± 0.1 | 1.7 ± 0.1 |  |
| 16:0/20:4 ** | | 1.9 ± 0.1 | 1.7 ± 0.2 | 1.3 ± 0.1 | 1.1 ± 0.1 |  |
| 16:0p/20:4 *** | | 1.7 ± 0.1 | 1.6 ± 0.1 | 1.0 ± 0.1 | 1.0 ± 0.1 |  |
| 18:0/20:4 *** | | 15.0 ± 0.4 | 13.6 ± 0.8 | 10.4 ± 0.7 | 9.5 ± 0.4 |  |
| 18:0p/20:4 *** | | 4.7 ± 0.2 | 5.0 ± 0.2 | 2.5 ± 0.1 | 2.6 ± 0.2 |  |
| 18:0e/20:4 *** | | 0.4 ± tr. | 0.4 ± tr. | 0.2 ± tr. | 0.2 ± tr. |  |
| 18:1/20:4 *** | | 1.7 ± 0.1 | 1.7 ± 0.1 | 1.0 ± 0.1 | 0.9 ± tr. |  |
| 16:0p/22:4 *** | | 3.3 ± 0.1 | 3.1 ± 0.1 | 1.3 ± 0.1 | 1.2 ± 0.1 |  |
| 18:0/22:4 *** | | 2.5 ± 0.1 | 2.6 ± 0.1 | 1.1 ± tr. | 1.0 ± 0.1 |  |
| 18:0p/22:4 or ***  18:1e/22:4 | | 4.2 ± 0.1 | 4.3 ± 0.2 | 1.7 ± 0.1 | 1.6 ± 0.1 |  |
| 16:0/22:5 | | 0.4 ± tr. | 0.4 ± tr. | 0.4 ± tr. | 0.4 ± tr. |  |
| 16:0p/22:5 *** | | 0.2 ± tr. | 0.2 ± tr. | 0.4 ± tr. | 0.4 ± tr. |  |
| 18:0p/22:5 *** | | 0.2 ± tr. | 0.2 ± tr. | 0.7 ± 0.1 | 0.7 ± 0.1 |  |
| 16:0/22:6 | | 1.5 ± 0.1 | 1.6 ± 0.2 | 1.8 ± 0.1 | 1.7 ± 0.1 |  |
| 16:0p/22:6 *** | | 2.0 ± tr. | 1.9 ± 0.1 | 2.6 ± 0.1 | 2.3 ± 0.1 |  |
| 18:0/22:6 ** | | 4.7 ± 0.2 | 5.0 ± 0.4 | 6.2 ± 0.2 | 5.5 ± 0.2 |  |
| 18:0p/22:6 *** | | 3.6 ± 0.1 | 3.9 ± 0.2 | 4.8 ± 0.2 | 4.8 ± 0.2 |  |
| 18:1/22:6 ** | | 0.4 ± tr. | 0.5 ± tr. | 0.5 ± tr. | 0.5 ± tr. |  |
| 18:1p/22:6 or ***  18:2e/22:6 | | 1.4 ± tr. | 1.4 ± 0.1 | 2.0 ± 0.1 | 1.9 ± 0.1 |  |

p, plasmenyl (or plasmalogen); e, plasmanyl; tr., trace (less than 0.05) significant effect of diet, ** p < 0.01, *** p < 0.001; significant effect of genotype, ^♦^ p < 0.05.

**Table 34**. Phosphatidylethanolamine (PE) molecular species composition of the hippocampus of wild-type (WT) and transgenic (Tg) mice on the oil blend diet (OB) or on the DHA diet, at 16 months of age. Results are represented as mean percentages of total molecular species analysed ± SEM. Analysis by LC-MS/MS.

|  | PE Hippocampus - 16 months | | | | | |
| --- | --- | --- | --- | --- | --- | --- |
| Molecular species | | Tg OB (n = 3) | WT OB (n = 3) | Tg DHA (n = 3) | WT DHA (n = 3) |  |
| 16:0/18:1 ** | | 3.3 ± 0.1 | 3.5 ± tr. | 4.1 ± 0.2 | 4.2 ± 0.2 |  |
| 16:0p/18:1 | | 3.3 ± 0.1 | 3.1 ± 0.1 | 3.8 ± 0.2 | 3.5 ± 0.3 |  |
| 18:0/18:0 ** | | 1.9 ± tr. | 2.0 ± 0.1 | 2.4 ± 0.2 | 2.4 ± 0.1 |  |
| 18:0/18:1 ^♦^ | | 5.9 ± 0.2 | 5.4 ± 0.3 | 6.3 ± 0.2 | 5.7 ± 0.2 |  |
| 18:0p/18:1 | | 4.5 ± 0.2 | 4.4 ± 0.5 | 5.8 ± 0.3 | 4.6 ± 0.3 |  |
| 18:1/18:1 *** ^♦^ | | 5.2 ± 0.1 | 5.8 ± 0.5 | 7.7 ± tr. | 8.8 ± 0.4 |  |
| 18:1p/18:1 ** | | 14.9 ± 0.6 | 13.5 ± 0.3 | 18.0 ± 1.5 | 17.0 ± 0.6 |  |
| 18:0/20:1 *** | | 1.1 ± tr. | 1.1 ± 0.1 | 1.5 ± 0.1 | 1.5 ± tr. |  |
| 18:1p/20:1 | | 4.3 ± 0.1 | 4.1 ± 0.2 | 4.7 ± 0.5 | 4.1 ± 0.2 |  |
| 18:1p/20:2 | | 0.3 ± 0.1 | 0.3 ± tr. | 0.4 ± 0.1 | 0.4 ± tr. |  |
| 18:0/20:3 *** | | 0.3 ± tr. | 0.4 ± 0.1 | 1.6 ± 0.1 | 1.4 ± 0.1 |  |
| 16:0/20:4 *** | | 1.7 ± 0.1 | 1.8 ± 0.1 | 1.1 ± 0.1 | 1.2 ± tr. |  |
| 16:0p/20:4 ** | | 1.2 ± 0.1 | 1.4 ± 0.1 | 0.6 ± 0.2 | 0.8 ± 0.1 |  |
| 18:0/20:4 ** | | 19.3 ± 0.4 | 17.9 ± 1.7 | 12.1 ± 0.9 | 13.8 ± 0.6 |  |
| 18:0p/20:4 *** | | 4.5 ± 0.2 | 5.0 ± 0.4 | 2.1 ± 0.1 | 2.5 ± 0.1 |  |
| 18:0e/20:4 ** | | 0.3 ± 0.1 | 0.4 ± tr. | 0.1 ± tr. | 0.1 ± tr. |  |
| 18:1/20:4 *** | | 1.4 ± tr. | 1.5 ± 0.1 | 0.7 ± 0.1 | 0.8 ± tr. |  |
| 16:0p/22:4 *** | | 3.4 ± 0.1 | 3.1 ± 0.2 | 1.0 ± 0.1 | 1.1 ± 0.1 |  |
| 18:0/22:4 *** | | 2.9 ± 0.1 | 2.9 ± tr. | 0.9 ± 0.1 | 0.9 ± tr. |  |
| 18:0p/22:4 or ***  18:1e/22:4 | | 4.0 ± 0.1 | 3.7 ± 0.1 | 1.2 ± 0.1 | 1.4 ± tr. |  |
| 16:0/22:5 * | | 0.6 ± 0.1 | 0.6 ± 0.1 | 0.8 ± 0.1 | 0.8 ± tr. |  |
| 16:0p/22:5 ** | | 0.1 ± tr. | 0.1 ± tr. | 0.3 ± tr. | 0.4 ± 0.1 |  |
| 18:0p/22:5 *** | | 0.1 ± tr. | 0.1 ± tr. | 0.5 ± tr. | 0.4 ± 0.1 |  |
| 16:0/22:6 ** | | 1.8 ± tr. | 2.1 ± 0.2 | 2.5 ± 0.1 | 2.5 ± 0.1 |  |
| 16:0p/22:6 ** | | 1.9 ± 0.1 | 2.0 ± 0.1 | 2.6 ± 0.1 | 2.4 ± 0.1 |  |
| 18:0/22:6 ** | | 5.9 ± 0.1 | 6.7 ± 0.5 | 8.7 ± 0.6 | 8.3 ± 0.1 |  |
| 18:0p/22:6 *** ^♦^ | | 3.9 ± 0.1 | 4.9 ± 0.2 | 5.9 ± 0.3 | 6.1 ± 0.2 |  |
| 18:1/22:6 ** | | 0.6 ± tr. | 0.7 ± tr. | 0.9 ± tr. | 0.9 ± tr. |  |
| 18:1p/22:6 or ***  18:2e/22:6 | | 1.5 ± tr. | 1.5 ± 0.1 | 2.1 ± 0.1 | 2.1 ± 0.1 |  |

p, plasmenyl (or plasmalogen); e, plasmanyl; tr., trace (less than 0.05); significant effect of diet, * p < 0.05, ** p < 0.01, *** p < 0.001; significant effect of genotype, ^♦^ p < 0.05.

**Table 35**. Phosphatidylethanolamine (PE) molecular species composition of the cerebellum of wild-type (WT) and transgenic (Tg) mice on the oil blend diet (OB) or on the DHA diet, at 12 months of age. Results are represented as mean percentages of total molecular species analysed ± SEM. Analysis by LC-MS/MS.

|  | PE Cerebellum - 12 months | | | | | |
| --- | --- | --- | --- | --- | --- | --- |
| Molecular species | | Tg OB (n = 6) | WT OB (n = 6) | Tg DHA (n = 6) | WT DHA (n = 6) |  |
| 16:0/18:1 *** | | 2.5 ± 0.1 | 2.3 ± tr. | 2.9 ± 0.1 | 2.9 ± 0.1 |  |
| 16:0p/18:1 *** | | 9.3 ± 0.2 | 9.1 ± 0.2 | 10.9 ± 0.5 | 10.7 ± 0.3 |  |
| 18:0/18:0 *** | | 0.9 ± tr. | 0.9 ± 0.1 | 1.2 ± 0.1 | 1.1 ± 0.1 |  |
| 18:0/18:1 * | | 5.6 ± 0.1 | 5.5 ± 0.1 | 5.3 ± 0.2 | 5.2 ± 0.1 |  |
| 18:0p/18:1 | | 13.1 ± 0.2 | 13.2 ± 0.2 | 13.6 ± 0.3 | 13.4 ± 0.1 |  |
| 18:1/18:1 *** | | 7.6 ± 0.1 | 7.3 ± 0.2 | 10.2 ± 0.3 | 10.0 ± 0.3 |  |
| 18:1p/18:1 *** | | 24.7 ± 0.3 | 25.6 ± 0.6 | 28.1 ± 0.5 | 28.7 ± 0.6 |  |
| 18:0/20:1 *** | | 0.3 ± tr. | 0.3 ± tr. | 0.4 ± tr. | 0.4 ± tr. |  |
| 18:1p/20:1 * ^♦♦^ | | 10.1 ± 0.2 | 10.4 ± 0.3 | 9.1 ± 0.2 | 10.3 ± 0.3 |  |
| 18:1p/20:2 *** | | 0.5 ± tr. | 0.5 ± tr. | 0.3 ± tr. | 0.3 ± tr. |  |
| 18:0/20:3 *** | | 0.3 ± tr. | 0.3 ± tr. | 0.7 ± 0.1 | 0.6 ± tr. |  |
| 16:0/20:4 *** | | 0.7 ± 0.1 | 0.6 ± 0.1 | 0.4 ± tr. | 0.4 ± tr. |  |
| 16:0p/20:4 *** | | 1.0 ± tr. | 0.9 ± tr. | 0.4 ± tr. | 0.4 ± tr. |  |
| 18:0/20:4 *** ^♦♦^ | | 5.0 ± 0.1 | 4.6 ± 0.1 | 2.2 ± 0.1 | 1.9 ± 0.1 |  |
| 18:0p/20:4 *** | | 3.5 ± 0.1 | 3.4 ± 0.1 | 1.2 ± tr. | 1.0 ± 0.1 |  |
| 18:0e/20:4 *** | | 0.3 ± tr. | 0.3 ± tr. | 0.1 ± tr. | 0.1 ± tr. |  |
| 18:1/20:4 *** | | 1.4 ± 0.1 | 1.4 ± tr. | 0.6 ± tr. | 0.6 ± tr. |  |
| 16:0p/22:4 *** | | 0.9 ± tr. | 0.9 ± tr. | 0.3 ± tr. | 0.3 ± tr. |  |
| 18:0/22:4 *** | | 0.7 ± tr. | 0.7 ± tr. | 0.2 ± tr. | 0.2 ± tr. |  |
| 18:0p/22:4 or ***  18:1e/22:4 | | 1.7 ± 0.1 | 1.8 ± 0.1 | 0.5 ± tr. | 0.5 ± tr. |  |
| 16:0/22:5 | | 0.2 ± tr. | 0.2 ± tr. | 0.3 ± tr. | 0.2 ± tr. |  |
| 16:0p/22:5 * | | 0.1 ± tr. | 0.1 ± tr. | 0.2 ± tr. | 0.2 ± tr. |  |
| 18:0p/22:5 *** | | 0.1 ± tr. | 0.1 ± tr. | 0.3 ± tr. | 0.4 ± tr. |  |
| 16:0/22:6 | | 0.9 ± 0.1 | 0.9 ± 0.1 | 1.0 ± 0.1 | 1.0 ± 0.1 |  |
| 16:0p/22:6 ** | | 0.9 ± tr. | 0.8 ± tr. | 1.2 ± 0.1 | 1.0 ± 0.1 |  |
| 18:0/22:6 | | 3.9 ± 0.2 | 3.9 ± 0.3 | 4.2 ± 0.2 | 4.2 ± 0.3 |  |
| 18:0p/22:6 | | 2.8 ± 0.1 | 2.9 ± 0.1 | 3.1 ± 0.1 | 3.0 ± 0.1 |  |
| 18:1/22:6 | | 0.4 ± tr. | 0.4 ± tr. | 0.4 ± tr. | 0.5 ± tr. |  |
| 18:1p/22:6 or ***  18:2e/22:6 | | 0.5 ± tr. | 0.5 ± tr. | 0.8 ± tr. | 0.7 ± tr. |  |

p, plasmenyl (or plasmalogen); e, plasmanyl; tr., trace (less than 0.05); significant effect of diet, * p < 0.05, ** p < 0.01, *** p < 0.001; significant effect of genotype, ^♦♦^ p < 0.01.

**Table 36**. Phosphatidylethanolamine (PE) molecular species composition of the cerebellum of wild-type (WT) and transgenic (Tg) mice on the oil blend diet (OB) or on the DHA diet, at 16 months of age. Results are represented as mean percentages of total molecular species analysed ± SEM. Analysis by LC-MS/MS.

|  | PE Cerebellum - 16 months | | | | | | | |  |  |
| --- | --- | --- | --- | --- | --- | --- | --- | --- | --- | --- |
| Molecular species | | Tg OB (n = 3) | WT OB (n = 3) | | Tg DHA (n = 3) | | WT DHA (n = 3) | | |  |
| 16:0/18:1 *** | | 3.2 ± 0.1 | | 3.6 ± 0.1 | | 4.6 ± 0.1 | | 4.2 ± 0.3 | | |
| 16:0p/18:1 * | | 9.1 ± 0.4 | | 7.6 ± 0.5 | | 10.2 ± 0.4 | | 9.4 ± 0.7 | | |
| 18:0/18:0 ** | | 0.8 ± 0.1 | | 0.9 ± 0.1 | | 1.2 ± tr. | | 1.2 ± tr. | | |
| 18:0/18:1 * | | 5.5 ± 0.1 | | 5.2 ± 0.1 | | 5.0 ± tr. | | 4.8 ± 0.2 | | |
| 18:0p/18:1 ** ^♦♦^ | | 10.5 ± 0.2 | | 9.6 ± 0.3 | | 11.6 ± 0.1 | | 10.3 ± 0.3 | | |
| 18:1/18:1 *** | | 8.3 ± 0.3 | | 8.8 ± 0.1 | | 11.3 ± 0.1 | | 11.7 ± 0.4 | | |
| 18:1p/18:1 * | | 23.8 ± 1.2 | | 24.0 ± 0.3 | | 25.3 ± 0.5 | | 27.6 ± 1.1 | | |
| 18:0/20:1 ** | | 0.4 ± tr. | | 0.4 ± tr. | | 0.6 ± tr. | | 0.6 ± tr. | | |
| 18:1p/20:1 | | 10.4 ± 0.5 | | 9.3 ± 0.5 | | 8.8 ± 0.3 | | 8.9 ± 0.4 | | |
| 18:1p/20:2 ** | | 0.5 ± tr. | | 0.4 ± tr. | | 0.2 ± tr. | | 0.3 ± tr. | | |
| 18:0/20:3 *** | | 0.2 ± tr. | | 0.2 ± tr. | | 0.4 ± tr. | | 0.5 ± 0.1 | | |
| 16:0/20:4 *** | | 0.7 ± tr. | | 0.8 ± 0.1 | | 0.4 ± 0.1 | | 0.4 ± tr. | | |
| 16:0p/20:4 *** | | 0.7 ± tr. | | 0.8 ± 0.1 | | 0.3 ± 0.1 | | 0.3 ± tr. | | |
| 18:0/20:4 *** | | 6.0 ± 0.7 | | 6.6 ± 0.3 | | 2.2 ± 0.2 | | 2.6 ± 0.2 | | |
| 18:0p/20:4 *** | | 3.3 ± 0.2 | | 3.4 ± 0.2 | | 1.0 ± 0.2 | | 1.1 ± tr. | | |
| 18:0e/20:4 * | | 0.2 ± 0.1 | | 0.2 ± 0.1 | | N.D. | | 0.1 ± tr. | | |
| 18:1/20:4 *** | | 1.3 ± 0.1 | | 1.3 ± tr. | | 0.4 ± tr. | | 0.5 ± tr. | | |
| 16:0p/22:4 *** ^♦^ | | 1.1 ± 0.1 | | 0.9 ± tr. | | 0.2 ± tr. | | 0.2 ± tr. | | |
| 18:0/22:4 *** | | 0.9 ± 0.1 | | 0.8 ± tr. | | 0.1 ± tr. | | 0.2 ± tr. | | |
| 18:0p/22:4 or ***  18:1e/22:4 | | 1.8 ± 0.1 | | 1.7 ± tr. | | 0.4 ± tr. | | 0.4 ± tr. | | |
| 16:0/22:5 | | 0.4 ± tr. | | 0.4 ± tr. | | 0.4 ± tr. | | 0.4 ± tr. | | |
| 16:0p/22:5 * | | 0.1 ± tr. | | 0.1 ± tr. | | 0.1 ± tr. | | 0.1 ± tr. | | |
| 18:0p/22:5 ** | | 0.1 ± tr. | | 0.1 ± tr. | | 0.4 ± 0.1 | | 0.2 ± tr. | | |
| 16:0/22:6 * | | 1.1 ± 0.1 | | 1.4 ± 0.1 | | 1.5 ± 0.1 | | 1.5 ± 0.1 | | |
| 16:0p/22:6 *** | | 0.8 ± 0.1 | | 0.9 ± tr. | | 1.2 ± tr. | | 1.2 ± tr. | | |
| 18:0/22:6 * | | 5.0 ± 0.4 | | 5.7 ± 0.1 | | 6.5 ± 0.4 | | 6.0 ± 0.4 | | |
| 18:0p/22:6 * | | 2.8 ± 0.3 | | 3.6 ± tr. | | 3.9 ± 0.2 | | 3.9 ± 0.3 | | |
| 18:1/22:6 ** | | 0.6 ± tr. | | 0.7 ± tr. | | 0.8 ± tr. | | 0.7 ± tr. | | |
| 18:1p/22:6 or *** ^♦^  18:2e/22:6 | | 0.5 ± tr. | | 0.6 ± tr. | | 0.8 ± tr. | | 0.8 ± tr. | | |

p, plasmenyl (or plasmalogen); e, plasmanyl; tr., trace (less than 0.05); significant effect of diet, * p < 0.05, ** p < 0.01, *** p < 0.001; significant effect of genotype, ^♦^ p < 0.05, ^♦♦^ p < 0.01.

**Table 37**. Phosphatidylcholine (PC) molecular species composition of the cortex of wild-type (WT) and transgenic (Tg) mice on the oil blend diet (OB) or on the DHA diet, at 12 months and at 16 months of age. Results are represented as mean percentages of total molecular species analysed ± SEM. Analysis by LC-MS/MS.

|  | PC Cortex - 12 months | | | | | |
| --- | --- | --- | --- | --- | --- | --- |
| Molecular species | | Tg OB (n = 6) | WT OB (n = 6) | Tg DHA (n = 6) | WT DHA (n = 6) |  |
| 16:0/16:0 | | 18.2 ± 0.6 | 18.4 ± 0.1 | 18.1 ± 0.5 | 19.5 ± 0.4 |  |
| 16:0/16:1 ^♦♦^ | | 2.6 ± 0.1 | 1.9 ± 0.2 | 2.5 ± 0.2 | 2.2 ± 0.2 |  |
| 16:1/16:1 | | 1.7 ± 0.1 | 1.6 ± 0.1 | 1.6 ± 0.1 | 1.9 ± 0.1 |  |
| 16:0/18:0 | | 4.8 ± 0.2 | 4.6 ± 0.1 | 4.2 ± 0.2 | 4.6 ± 0.2 |  |
| 16:0/18:1 | | 34.3 ± 0.8 | 33.8 ± 0.9 | 35.8 ± 1.1 | 35.3 ± 0.9 |  |
| 18:0/18:1 ^♦^ | | 6.6 ± 0.3 | 7.2 ± 0.4 | 6.4 ± 0.3 | 6.9 ± 0.2 |  |
| 16:0/18:2 * ^♦♦^ | | 2.1 ± tr. | 1.8 ± tr. | 2.2 ± 0.1 | 2.1 ± 0.1 |  |
| 18:0/18:2 | | 2.9 ± 0.2 | 2.8 ± 0.1 | 3.0 ± 0.1 | 3.0 ± 0.1 |  |
| 16:0p/20:0 or  16:0e/20:1 | | 0.7 ± tr. | 0.7 ± tr. | 0.6 ± tr. | 0.7 ± tr. |  |
| 16:0/20:3 ** | | 2.7 ± 0.1 | 2.7 ± 0.1 | 3.7 ± 0.3 | 3.4 ± 0.3 |  |
| 16:0/20:4 *** | | 7.1 ± 0.4 | 7.2 ± 0.4 | 4.4 ± 0.4 | 4.0 ± 0.3 |  |
| 18:0/20:4 *** | | 3.8 ± 0.2 | 3.9 ± 0.1 | 2.1 ± 0.1 | 1.9 ± 0.1 |  |
| 18:1/20:4 ** | | 3.1 ± 0.2 | 3.0 ± 0.1 | 2.6 ± 0.1 | 2.3 ± 0.1 |  |
| 18:0/18:0 or  16:0p/22:6 | | 1.6 ± 0.1 | 1.7 ± 0.1 | 1.6 ± 0.1 | 1.7 ± 0.1 |  |
| 16:0/22:6 ** | | 5.5 ± 0.5 | 6.0 ± 0.2 | 7.7 ± 0.4 | 7.4 ± 0.6 |  |
| 18:0/22:6 *** | | 1.0 ± tr. | 1.3 ± 0.1 | 1.5 ± 0.1 | 1.5 ± 0.1 |  |
| 18:1/22:6 * | | 1.4 ± 0.1 | 1.4 ± 0.1 | 2.0 ± 0.2 | 1.7 ± 0.1 |  |
|  | PC Cortex - 16 months | | | | | |
| Molecular species | | Tg OB (n = 3) | WT OB (n = 3) | Tg DHA (n = 3) | WT DHA (n = 3) |  |
| 16:0/16:0 | | 5.0 ± 0.1 | 5.6 ± 0.1 | 6.8 ± 1.3 | 5.4 ± 0.1 |  |
| 16:0/16:1 | | 0.3 ± tr. | 0.2 ± 0.1 | 0.5 ± 0.1 | 0.4 ± 0.1 |  |
| 16:1/16:1 | | 0.1 ± tr. | 0.1 ± tr. | 0.2 ± tr. | 0.1 ± tr. |  |
| 16:0/18:0 | | 4.2 ± 0.1 | 3.4 ± 0.4 | 4.0 ± 0.2 | 3.8 ± 0.1 |  |
| 16:0/18:1 | | 19.4 ± 0.3 | 21.3 ± 1.2 | 19.8 ± 2.3 | 20.5 ± 0.5 |  |
| 18:0/18:1 | | 16.8 ± 0.3 | 16.6 ± 0.2 | 14.0 ± 2.0 | 15.6 ± 0.8 |  |
| 16:0/18:2 ^♦^ | | 1.9 ± tr. | 1.6 ± tr. | 2.1 ± 0.2 | 1.7 ± 0.1 |  |
| 18:0/18:2 | | 3.5 ± 0.1 | 3.3 ± 0.2 | 3.7 ± 0.1 | 3.8 ± 0.2 |  |
| 16:0p/20:0 or  16:0e/20:1 | | 1.0 ± 0.1 | 0.8 ± tr. | 0.9 ± 0.1 | 1.0 ± tr. |  |
| 16:0/20:3 *** | | 1.5 ± 0.1 | 1.1 ± 0.1 | 4.7 ± 0.9 | 3.6 ± 0.5 |  |
| 16:0/20:4 *** | | 5.8 ± 0.2 | 6.4 ± 0.3 | 3.0 ± 0.1 | 3.1 ± 0.1 |  |
| 18:0/20:4 *** | | 8.9 ± 0.1 | 9.6 ± 0.6 | 3.9 ± 0.5 | 4.0 ± 0.5 |  |
| 18:1/20:4 * | | 10.9 ± 0.1 | 9.5 ± 1.5 | 6.2 ± 0.8 | 9.6 ± 0.3 |  |
| 18:0/18:0 or  16:0p/22:6 | | 3.5 ± 0.1 | 3.1 ± 0.1 | 2.9 ± 0.2 | 3.2 ± 0.2 |  |
| 16:0/22:6 ** | | 10.4 ± 0.6 | 10.3 ± 0.3 | 16.8 ± 2.0 | 14.6 ± 0.1 |  |
| 18:0/22:6 ** | | 3.5 ± 0.1 | 3.7 ± 0.1 | 5.2 ± 0.5 | 4.5 ± 0.1 |  |
| 18:1/22:6 ** | | 3.2 ± 0.1 | 3.4 ± 0.3 | 5.4 ± 1.0 | 5.1 ± 0.1 |  |

tr., trace (less than 0.05); significant effect of diet, * p < 0.05, ** p < 0.01, *** p < 0.001; significant effect of genotype, ^♦^ p < 0.05, ^♦♦^ p < 0.01.

**Table 38**. Phosphatidylcholine (PC) molecular species composition of the hippocampus of wild-type (WT) and transgenic (Tg) mice on the oil blend diet (OB) or on the DHA diet, at 12 months and at 16 months of age. Results are represented as mean percentages of total molecular species analysed ± SEM. Analysis by LC-MS/MS.

|  | PC Hippocampus - 12 months | | | | | |
| --- | --- | --- | --- | --- | --- | --- |
| Molecular species | | Tg OB (n = 6) | WT OB (n = 6) | Tg DHA (n = 6) | WT DHA (n = 6) |  |
| 16:0/16:0 | | 19.5 ± 0.4 | 20.2 ± 0.5 | 19.8 ± 0.4 | 19.2 ± 0.5 |  |
| 16:0/16:1 | | 1.7 ± 0.1 | 2.0 ± 0.2 | 2.2 ± 0.1 | 1.7 ± 0.1 |  |
| 16:1/16:1 | | 2.3 ± 0.1 | 2.5 ± 0.2 | 2.3 ± 0.1 | 2.4 ± 0.1 |  |
| 16:0/18:0 | | 3.7 ± 0.1 | 3.6 ± 0.2 | 3.4 ± 0.1 | 3.6 ± 0.1 |  |
| 16:0/18:1 ** | | 38.2 ± 0.4 | 36.1 ± 0.7 | 39.1 ± 0.9 | 39.8 ± 0.4 |  |
| 18:0/18:1 | | 6.6 ± 0.2 | 6.9 ± 0.4 | 6.6 ± 0.2 | 6.9 ± 0.3 |  |
| 16:0/18:2 ** ^♦^ | | 1.6 ± tr. | 1.5 ± 0.1 | 2.0 ± 0.1 | 1.7 ± 0.1 |  |
| 18:0/18:2 *** | | 2.2 ± 0.1 | 2.3 ± 0.1 | 2.6 ± 0.1 | 2.6 ± 0.1 |  |
| 16:0p/20:0 or  16:0e/20:1 | | 0.6 ± tr. | 0.6 ± tr. | 0.6 ± tr. | 0.6 ± tr. |  |
| 16:0/20:3 ** | | 2.6 ± tr. | 2.4 ± 0.1 | 3.1 ± 0.2 | 2.9 ± 0.2 |  |
| 16:0/20:4 *** | | 7.3 ± 0.4 | 7.1 ± 0.1 | 4.3 ± 0.3 | 4.2 ± 0.3 |  |
| 18:0/20:4 *** | | 3.6 ± 0.1 | 3.6 ± 0.2 | 2.2 ± 0.1 | 2.1 ± 0.1 |  |
| 18:1/20:4 *** | | 3.1 ± 0.1 | 3.2 ± 0.1 | 2.6 ± 0.1 | 2.5 ± 0.1 |  |
| 18:0/18:0 or  16:0p/22:6 | | 1.7 ± tr. | 1.7 ± 0.1 | 1.7 ± 0.1 | 1.8 ± 0.1 |  |
| 16:0/22:6 *** | | 3.9 ± 0.3 | 4.3 ± 0.3 | 5.3 ± 0.3 | 5.7 ± 0.4 |  |
| 18:0/22:6 ** | | 0.7 ± tr. | 0.9 ± 0.1 | 1.0 ± 0.1 | 1.0 ± 0.1 |  |
| 18:1/22:6 ** | | 0.7 ± 0.1 | 1.1 ± 0.1 | 1.3 ± 0.1 | 1.2 ± 0.1 |  |
|  | PC Hippocampus - 16 months | | | | | |
| Molecular species | | Tg OB (n = 3) | WT OB (n = 3) | Tg DHA (n = 3) | WT DHA (n = 3) |  |
| 16:0/16:0 | | 4.9 ± 0.1 | 5.4 ± 0.4 | 4.9 ± 0.1 | 5.1 ± 0.2 |  |
| 16:0/16:1 | | 0.4 ± 0.1 | 0.4 ± 0.1 | 0.6 ± tr. | 0.3 ± 0.1 |  |
| 16:1/16:1 | | 0.1 ± tr. | 0.1 ± tr. | 0.1 ± tr. | 0.2 ± tr. |  |
| 16:0/18:0 ^♦^ | | 2.5 ± 0.2 | 3.5 ± 0.3 | 2.7 ± 0.1 | 2.8 ± 0.2 |  |
| 16:0/18:1 * | | 21.9 ± 0.1 | 21.4 ± 1.0 | 23.7 ± 0.8 | 24.0 ± 1.1 |  |
| 18:0/18:1 | | 17.0 ± 0.4 | 17.4 ± 0.8 | 17.4 ± 0.7 | 16.8 ± 0.4 |  |
| 16:0/18:2 ** ^♦♦^ | | 1.7 ± 0.1 | 1.5 ± 0.1 | 2.2 ± 0.1 | 1.6 ± 0.1 |  |
| 18:0/18:2 ** | | 3.4 ± 0.1 | 3.3 ± 0.2 | 4.2 ± 0.1 | 4.1 ± 0.3 |  |
| 16:0p/20:0 or  16:0e/20:1 | | 0.8 ± tr. | 0.8 ± tr. | 0.7 ± 0.1 | 0.9 ± tr. |  |
| 16:0/20:3 | | 2.1 ± 1.2 | 1.0 ± 0.2 | 2.3 ± 0.2 | 2.2 ± 0.2 |  |
| 16:0/20:4 *** | | 7.6 ± 0.3 | 6.4 ± 0.8 | 3.9 ± 0.2 | 4.4 ± 0.2 |  |
| 18:0/20:4 *** | | 12.4 ± 0.4 | 11.1 ± 1.4 | 6.5 ± 0.4 | 7.7 ± 0.2 |  |
| 18:1/20:4 *** | | 7.9 ± 0.1 | 8.3 ± 1.1 | 4.0 ± 0.3 | 5.1 ± 0.3 |  |
| 18:0/18:0 or  16:0p/22:6 | | 3.4 ± 0.1 | 3.5 ± 0.2 | 3.4 ± 0.1 | 3.3 ± 0.3 |  |
| 16:0/22:6 ** | | 8.4 ± 0.4 | 9.5 ± 0.9 | 14.0 ± 0.3 | 12.6 ± 1.2 |  |
| 18:0/22:6 *** | | 3.1 ± 0.2 | 3.4 ± 0.1 | 4.7 ± tr. | 4.6 ± tr. |  |
| 18:1/22:6 *** | | 2.5 ± 0.3 | 2.8 ± 0.3 | 4.6 ± 0.3 | 4.3 ± 0.2 |  |

tr., trace (less than 0.05); significant effect of diet, * p < 0.05, ** p < 0.01, *** p < 0.001; significant effect of genotype, ^♦^ p < 0.05, ^♦♦^ p < 0.01.

**Table 39**. Phosphatidylcholine (PC) molecular species composition of the cerebellum of wild-type (WT) and transgenic (Tg) mice on the oil blend diet (OB) or on the DHA diet, at 12 months and at 16 months of age. Results are represented as mean percentages of total molecular species analysed ± SEM. Analysis by LC-MS/MS.

|  | PC Cerebellum - 12 months | | | | | |
| --- | --- | --- | --- | --- | --- | --- |
| Molecular species | | Tg OB (n = 6) | WT OB (n = 6) | Tg DHA (n = 6) | WT DHA (n = 6) |  |
| 16:0/16:0 * | | 14.6 ± 0.4 | 14.0 ± 0.5 | 14.6 ± 0.4 | 16.1 ± 0.4 |  |
| 16:0/16:1 ** | | 1.3 ± 0.1 | 1.5 ± 0.2 | 1.8 ± 0.1 | 1.9 ± 0.2 |  |
| 16:1/16:1 | | 2.1 ± 0.1 | 2.1 ± 0.1 | 1.9 ± 0.1 | 2.2 ± 0.1 |  |
| 16:0/18:0 | | 5.0 ± 0.2 | 4.9 ± 0.3 | 4.8 ± 0.1 | 4.5 ± 0.3 |  |
| 16:0/18:1 | | 39.1 ± 0.6 | 37.9 ± 0.8 | 39.3 ± 0.9 | 38.7 ± 0.8 |  |
| 18:0/18:1 * | | 9.3 ± 0.3 | 9.5 ± 0.4 | 8.3 ± 0.5 | 8.4 ± 0.3 |  |
| 16:0/18:2 * | | 1.7 ± tr. | 1.6 ± 0.1 | 2.0 ± 0.1 | 1.9 ± 0.1 |  |
| 18:0/18:2 | | 2.9 ± 0.1 | 3.0 ± 0.1 | 3.1 ± 0.1 | 3.0 ± tr. |  |
| 16:0p/20:0 or *  16:0e/20:1 | | 0.9 ± tr. | 1.0 ± tr. | 0.9 ± tr. | 0.9 ± tr. |  |
| 16:0/20:3 | | 1.6 ± tr. | 1.5 ± 0.1 | 1.6 ± 0.1 | 1.4 ± 0.1 |  |
| 16:0/20:4 *** | | 3.5 ± 0.3 | 3.3 ± 0.2 | 1.2 ± 0.2 | 1.0 ± 0.1 |  |
| 18:0/20:4 *** | | 1.7 ± tr. | 1.7 ± 0.1 | 0.5 ± tr. | 0.5 ± tr. |  |
| 18:1/20:4 * | | 2.0 ± 0.1 | 2.1 ± 0.1 | 1.9 ± 0.1 | 1.7 ± 0.1 |  |
| 18:0/18:0 or *  16:0p/22:6 | | 2.3 ± 0.1 | 2.4 ± 0.1 | 2.1 ± 0.1 | 2.2 ± 0.1 |  |
| 16:0/22:6 *** | | 7.0 ± 0.4 | 7.5 ± 0.6 | 9.8 ± 0.3 | 9.3 ± 0.7 |  |
| 18:0/22:6 ** | | 2.8 ± 0.1 | 3.1 ± 0.1 | 3.4 ± 0.1 | 3.2 ± 0.2 |  |
| 18:1/22:6 | | 2.1 ± 0.2 | 2.8 ± 0.2 | 2.9 ± 0.2 | 3.0 ± 0.3 |  |
|  | PC Cerebellum - 16 months | | | | | |
| Molecular species | | Tg OB (n = 3) | WT OB (n = 3) | Tg DHA (n = 3) | WT DHA (n = 3) |  |
| 16:0/16:0 | | 3.0 ± 0.2 | 3.6 ± 0.2 | 3.6 ± 0.3 | 3.4 ± 0.2 |  |
| 16:0/16:1 | | 0.2 ± 0.1 | 0.2 ± 0.1 | 0.2 ± tr. | 0.2 ± 0.1 |  |
| 16:1/16:1 | | N.D. | N.D. | N.D. | N.D. |  |
| 16:0/18:0 | | 3.6 ± 0.2 | 3.6 ± 0.3 | 3.7 ± 0.4 | 3.2 ± 0.3 |  |
| 16:0/18:1 | | 20.9 ± 0.7 | 20.3 ± 0.6 | 18.6 ± 1.1 | 19.9 ± 0.6 |  |
| 18:0/18:1 *** | | 23.0 ± 0.3 | 21.1 ± 0.2 | 17.5 ± 1.0 | 18.5 ± 0.4 |  |
| 16:0/18:2 ** | | 1.3 ± 0.2 | 1.3 ± tr. | 1.7 ± 0.1 | 1.6 ± tr. |  |
| 18:0/18:2 | | 4.1 ± 0.2 | 4.1 ± tr. | 4.1 ± 0.2 | 4.2 ± 0.1 |  |
| 16:0p/20:0 or  16:0e/20:1 | | 1.1 ± tr. | 1.1 ± 0.1 | 1.0 ± 0.1 | 1.1 ± 0.1 |  |
| 16:0/20:3 *** | | 0.7 ± tr. | 0.8 ± 0.1 | 1.5 ± tr. | 1.5 ± 0.2 |  |
| 16:0/20:4 *** | | 2.5 ± 0.2 | 2.5 ± 0.2 | 0.7 ± 0.1 | 0.9 ± tr. |  |
| 18:0/20:4 *** | | 4.7 ± 0.2 | 4.6 ± 0.3 | 0.7 ± tr. | 1.0 ± 0.1 |  |
| 18:1/20:4 | | 2.1 ± 0.1 | 1.9 ± 0.1 | 2.0 ± 0.4 | 3.2 ± 2.0 |  |
| 18:0/18:0 or *  16:0p/22:6 | | 4.5 ± 0.3 | 3.9 ± 0.1 | 3.6 ± 0.1 | 3.8 ± 0.1 |  |
| 16:0/22:6 *** | | 13.7 ± 0.4 | 14.1 ± 0.7 | 21.2 ± 0.3 | 18.9 ± 0.9 |  |
| 18:0/22:6 ** | | 9.8 ± 0.5 | 11.1 ± 0.9 | 13.0 ± 0.2 | 11.8 ± 0.5 |  |
| 18:1/22:6 ** | | 4.7 ± 0.2 | 5.6 ± 0.4 | 7.0 ± 0.7 | 6.7 ± 0.1 |  |

tr., trace (less than 0.05); significant effect of diet, * p < 0.05, ** p < 0.01, *** p < 0.001.

**Table 40**. Phosphatidylserine (PS) molecular species composition of the cortex of wild-type (WT) and transgenic (Tg) mice on the oil blend diet (OB) or on the DHA diet, at 12 months and 16 months of age. Results are represented as mean percentages of total molecular species analysed ± SEM. Analysis by LC-MS/MS.

|  | PS Cortex - 12 months | | | |
| --- | --- | --- | --- | --- |
| Molecular species | Tg OB (n = 6) | WT OB (n = 6) | Tg DHA (n = 6) | WT DHA (n = 6) |
| 16:0/18:1 | 4.3 ± 0.2 | 3.9 ± 0.3 | 3.6 ± 0.5 | 3.7 ± 0.3 |
| 18:0/18:1 | 49.5 ± 1.0 | 48.0 ± 0.8 | 51.4 ± 0.5 | 49.7 ± 1.2 |
| 18:0/20:0 | 4.8 ± 0.3 | 3.6 ± 0.3 | 4.3 ± 0.1 | 4.3 ± 0.4 |
| 18:0/20:4 *** ^♦^ | 6.6 ± 0.6 | 7.5 ± 0.3 | 4.0 ± 0.3 | 3.7 ± 0.4 |
| 18:0/22:6 | 34.8 ± 1.2 | 37.1 ± 1.0 | 36.8 ± 1.1 | 38.5 ± 1.1 |
|  | PS Cortex - 16 months | | | |
| Molecular species | Tg OB (n = 3) | WT OB (n = 3) | Tg DHA (n = 3) | WT DHA (n = 3) |
| 16:0/18:1 | 1.7 ± 0.5 | 2.2 ± 0.4 | 1.9 ± 0.1 | 2.0 ± 0.3 |
| 18:0/18:1 | 45.1 ± 0.7 | 45.1 ± 2.7 | 39.5 ± 1.2 | 44.4 ± 4.2 |
| 18:0/20:0 | 3.9 ± 0.8 | 3.4 ± 0.3 | 4.0 ± 0.3 | 3.4 ± 0.6 |
| 18:0/20:4 *** | 6.5 ± 0.4 | 6.1 ± 0.5 | 2.7 ± 0.3 | 3.1 ± 0.1 |
| 18:0/22:6 | 42.8 ± 1.5 | 43.2 ± 2.7 | 51.9 ± 1.4 | 47.0 ± 4.9 |

significant effect of diet, *** p < 0.001; significant effect of genotype, ^♦^ p < 0.05.

**Table 41**. Phosphatidylserine (PS) molecular species composition of the hippocampus of wild-type (WT) and transgenic (Tg) mice on the oil blend diet (OB) or on the DHA diet, at 12 months and 16 months of age. Results are represented as mean percentages of total molecular species analysed ± SEM. Analysis by LC-MS/MS.

|  | PS Hippocampus - 12 months | | | |
| --- | --- | --- | --- | --- |
| Molecular species | Tg OB (n = 6) | WT OB (n = 6) | Tg DHA (n = 6) | WT DHA (n = 6) |
| 16:0/18:1 | 6.4 ± 1.0 | 4.8 ± 0.6 | 6.0 ± 0.5 | 5.2 ± 0.7 |
| 18:0/18:1 | 49.7 ± 3.0 | 49.4 ± 2.9 | 50.5 ± 2.2 | 50.5 ± 1.6 |
| 18:0/20:0 | 2.9 ± 0.4 | 3.0 ± 0.2 | 3.8 ± 0.6 | 3.6 ± 0.5 |
| 18:0/20:4 ** | 7.0 ± 0.7 | 8.6 ± 1.6 | 4.5 ± 0.8 | 4.6 ± 0.6 |
| 18:0/22:6 | 34.0 ± 1.9 | 34.2 ± 2.3 | 35.1 ± 2.2 | 36.2 ± 1.6 |
|  | PS Hippocampus - 16 months | | | |
| Molecular species | Tg OB (n = 3) | WT OB (n = 3) | Tg DHA (n = 3) | WT DHA (n = 3) |
| 16:0/18:1 | 3.2 ± 0.3 | 3.2 ± 0.8 | 2.5 ± 0.2 | 3.4 ± 0.5 |
| 18:0/18:1 | 43.8 ± 1.1 | 47.1 ± 3.5 | 40.1 ± 2.7 | 41.1 ± 2.2 |
| 18:0/20:0 | 3.4 ± 0.2 | 3.4 ± 0.5 | 5.1 ± 0.4 | 4.3 ± 1.0 |
| 18:0/20:4 ** | 10.4 ± 1.6 | 7.1 ± 0.4 | 3.8 ± 0.1 | 5.7 ± 1.5 |
| 18:0/22:6 | 39.1 ± 2.5 | 39.2 ± 4.2 | 48.5 ± 2.0 | 45.5 ± 1.9 |

significant effect of diet, ** p < 0.01.

**Table 42**. Phosphatidylserine (PS) molecular species composition of the cerebellum of wild-type (WT) and transgenic (Tg) mice on the oil blend diet (OB) or on the DHA diet, at 12 months and 16 months of age. Results are represented as mean percentages of total molecular species analysed ± SEM. Analysis by LC-MS/MS.

|  | PS Cerebellum - 12 months | | | |
| --- | --- | --- | --- | --- |
| Molecular species | Tg OB (n = 6) | WT OB (n = 6) | Tg DHA (n = 6) | WT DHA (n = 6) |
| 16:0/18:1 | 4.9 ± 1.0 | 4.8 ± 0.4 | 3.8 ± 0.4 | 5.0 ± 0.2 |
| 18:0/18:1 | 65.7 ± 2.0 | 65.6 ± 0.8 | 66.4 ± 1.9 | 66.6 ± 1.9 |
| 18:0/20:0 | 3.5 ± 0.3 | 3.4 ± 0.4 | 3.5 ± 0.3 | 3.5 ± 0.3 |
| 18:0/20:4 *** | 4.3 ± 0.2 | 5.1 ± 0.5 | 2.4 ± 0.3 | 2.7 ± 0.3 |
| 18:0/22:6 | 21.6 ± 1.2 | 21.0 ± 0.5 | 23.9 ± 1.5 | 22.2 ± 1.4 |
|  | PS Cerebellum - 16 months | | | |
| Molecular species | Tg OB (n = 3) | WT OB (n = 3) | Tg DHA (n = 3) | WT DHA (n = 3) |
| 16:0/18:1 | 2.6 ± 0.1 | 3.2 ± 0.9 | 2.4 ± 0.5 | 2.8 ± 0.7 |
| 18:0/18:1 | 61.9 ± 2.8 | 58.5 ± 1.1 | 63.1 ± 3.1 | 59.8 ± 1.3 |
| 18:0/20:0 | 5.6 ± 0.6 | 5.8 ± 0.8 | 5.8 ± 0.6 | 5.3 ± 0.3 |
| 18:0/20:4 ** | 5.7 ± 0.6 | 5.5 ± 1.0 | 1.9 ± 0.9 | 3.4 ± 0.3 |
| 18:0/22:6 | 24.1 ± 2.8 | 27.1 ± 2.1 | 26.8 ± 2.1 | 28.7 ± 2.5 |

significant effect of diet, ** p < 0.01, *** p < 0.001.

**Table 43**. Phosphatidylinositol (PI) molecular species composition of the cortex of wild-type (WT) and transgenic (Tg) mice on the oil blend diet (OB) or on the DHA diet, at 12 months and 16 months of age. Results are represented as mean percentages of total molecular species analysed ± SEM. Analysis by LC-MS/MS.

|  | PI Cortex - 12 months | | | |
| --- | --- | --- | --- | --- |
| Molecular species | Tg OB (n = 6) | WT OB (n = 6) | Tg DHA (n = 6) | WT DHA (n = 6) |
| 16:0/18:1 | 2.1 ± 0.3 | 2.0 ± 0.3 | 2.6 ± 0.4 | 2.9 ± 0.7 |
| 18:0/18:1 ** | 5.1 ± 0.8 | 4.9 ± 0.6 | 7.5 ± 0.8 | 7.8 ± 0.8 |
| 16:0/20:3 ** | 1.5 ± 0.2 | 1.7 ± 0.2 | 2.6 ± 0.4 | 2.9 ± 0.6 |
| 16:0/20:4 | 44.9 ± 6.6 | 51.0 ± 7.6 | 39.2 ± 7.5 | 40.0 ± 7.1 |
| 18:0/20:4 | 40.6 ± 7.4 | 35.0 ± 7.6 | 40.8 ± 8.1 | 37.2 ± 7.8 |
| 16:0/22:6 | 4.3 ± 1.0 | 3.7 ± 1.0 | 4.7 ± 1.4 | 6.5 ± 1.5 |
| 18:0/22:6 | 1.5 ± 0.4 | 1.7 ± 0.3 | 2.6 ± 0.5 | 2.7 ± 0.7 |
|  | PI Cortex - 16 months | | | |
| Molecular species | Tg OB (n = 3) | WT OB (n = 3) | Tg DHA (n = 3) | WT DHA (n = 3) |
| 16:0/18:1 * | 1.6 ± 0.1 | 2.1 ± 0.5 | 3.1 ± 0.1 | 2.2 ± 0.3 |
| 18:0/18:1 ** | 0.9 ± 0.2 | 1.9 ± 0.3 | 2.8 ± 0.4 | 2.2 ± 0.3 |
| 16:0/20:3 | 0.8 ± 0.1 | 1.1 ± 0.2 | 0.8 ± 0.1 | 0.7 ± 0.1 |
| 16:0/20:4 | 43.7 ± 1.4 | 39.0 ± 3.6 | 36.5 ± 0.6 | 38.9 ± 1.4 |
| 18:0/20:4 | 45.7 ± 1.3 | 49.1 ± 3.4 | 44.6 ± 1.0 | 43.3 ± 0.4 |
| 16:0/22:6 *** | 4.9 ± 0.3 | 4.4 ± 0.4 | 8.5 ± tr. | 9.3 ± 1.1 |
| 18:0/22:6 ** | 2.4 ± 0.2 | 2.5 ± 0.3 | 3.9 ± 0.3 | 3.4 ± 0.4 |

tr., trace (less than 0.05); significant effect of diet, * p < 0.05, ** p < 0.01, *** p < 0.001.

**Table 44**. Phosphatidylinositol (PI) molecular species composition of the hippocampus of wild-type (WT) and transgenic (Tg) mice on the oil blend diet (OB) or on the DHA diet, at 12 months and 16 months of age. Results are represented as mean percentages of total molecular species analysed ± SEM. Analysis by LC-MS/MS.

|  | PI Hippocampus - 12 months | | | |
| --- | --- | --- | --- | --- |
| Molecular species | Tg OB (n = 6) | WT OB (n = 6) | Tg DHA (n = 6) | WT DHA (n = 6) |
| 16:0/18:1 | 2.2 ± 0.5 | 2.4 ± 0.5 | 2.9 ± 0.4 | 2.6 ± 0.3 |
| 18:0/18:1 * | 6.1 ± 0.9 | 6.1 ± 0.5 | 10.3 ± 1.4 | 8.3 ± 1.4 |
| 16:0/20:3 * | 1.2 ± 0.3 | 1.5 ± 0.5 | 2.5 ± 0.5 | 2.9 ± 0.5 |
| 16:0/20:4 | 34.2 ± 6.3 | 36.6 ± 8.0 | 32.2 ± 7.3 | 34.3 ± 5.8 |
| 18:0/20:4 | 52.7 ± 7.1 | 49.9 ± 8.3 | 46.4 ± 7.7 | 46.2 ± 7.1 |
| 16:0/22:6 | 2.2 ± 0.4 | 1.7 ± 0.4 | 3.5 ± 0.7 | 3.2 ± 1.0 |
| 18:0/22:6 | 1.4 ± 0.2 | 1.7 ± 0.4 | 2.3 ± 0.6 | 2.5 ± 0.4 |
|  | PI Hippocampus - 16 months | | | |
| Molecular species | Tg OB (n = 3) | WT OB (n = 3) | Tg DHA (n = 3) | WT DHA (n = 3) |
| 16:0/18:1 | 1.6 ± 0.2 | 1.6 ± 0.5 | 3.1 ± 1.2 | 2.6 ± 0.2 |
| 18:0/18:1 | 2.8 ± 0.3 | 2.5 ± 1.0 | 4.0 ± 0.2 | 2.6 ± 1.1 |
| 16:0/20:3 | 0.4 ± tr. | 0.5 ± 0.1 | 0.3 ± 0.2 | 0.8 ± 0.4 |
| 16:0/20:4 | 30.7 ± 0.9 | 36.5 ± 3.4 | 31.0 ± 0.7 | 31.2 ± 1.2 |
| 18:0/20:4 | 59.1 ± 0.8 | 53.9 ± 4.0 | 54.7 ± 0.8 | 55.9 ± 0.5 |
| 16:0/22:6 | 2.9 ± 0.5 | 3.1 ± 1.1 | 4.4 ± 0.8 | 3.4 ± 1.0 |
| 18:0/22:6 | 2.4 ± 0.5 | 1.7 ± 0.2 | 2.5 ± 0.4 | 3.6 ± 0.7 |

tr., trace (less than 0.05); significant effect of diet, * p < 0.05.

**Table 45**. Phosphatidylinositol (PI) molecular species composition of the cerebellum of wild-type (WT) and transgenic (Tg) mice on the oil blend diet (OB) or on the DHA diet, at 12 months and 16 months of age. Results are represented as mean percentages of total molecular species analysed ± SEM. Analysis by LC-MS/MS.

|  | PI Cerebellum - 12 months | | | |
| --- | --- | --- | --- | --- |
| Molecular species | Tg OB (n = 6) | WT OB (n = 6) | Tg DHA (n = 6) | WT DHA (n = 6) |
| 16:0/18:1 | 4.0 ± 0.6 | 3.2 ± 0.8 | 4.4 ± 0.6 | 5.8 ± 1.0 |
| 18:0/18:1 | 8.3 ± 1.4 | 7.7 ± 1.3 | 10.9 ± 1.2 | 9.0 ± 0.8 |
| 16:0/20:3 | 1.0 ± 0.3 | 1.2 ± 0.3 | 1.5 ± 0.3 | 1.8 ± 0.4 |
| 16:0/20:4 | 24.3 ± 6.4 | 26.0 ± 5.5 | 20.8 ± 4.2 | 17.5 ± 4.9 |
| 18:0/20:4 | 50.8 ± 7.2 | 50.3 ± 6.0 | 41.0 ± 5.6 | 44.7 ± 5.0 |
| 16:0/22:6 * | 5.1 ± 0.8 | 5.7 ± 1.6 | 13.5 ± 2.4 | 9.3 ± 2.6 |
| 18:0/22:6 * | 6.4 ± 1.0 | 5.9 ± 0.9 | 8.0 ± 1.8 | 11.9 ± 1.8 |
|  | PI Cerebellum - 16 months | | | |
| Molecular species | Tg OB (n = 3) | WT OB (n = 3) | Tg DHA (n = 3) | WT DHA (n = 3) |
| 16:0/18:1 | 3.7 ± 0.6 | 4.2 ± 1.0 | 5.7 ± 1.2 | 5.3 ± 0.7 |
| 18:0/18:1 * | 2.2 ± 0.1 | 2.5 ± 1.3 | 6.2 ± 1.0 | 5.2 ± 1.4 |
| 16:0/20:3 | 0.8 ± 0.2 | 0.2 ± 0.2 | 0.3 ± 0.1 | 0.3 ± 0.2 |
| 16:0/20:4 *** | 29.4 ± 1.8 | 30.2 ± 0.3 | 19.9 ± 1.1 | 21.8 ± 1.6 |
| 18:0/20:4 *** | 50.5 ± 1.6 | 49.5 ± 1.5 | 37.4 ± 1.0 | 42.9 ± 2.1 |
| 16:0/22:6 *** ^♦^ | 9.1 ± 0.1 | 6.7 ± 1.3 | 15.7 ± 0.7 | 12.9 ± 1.0 |
| 18:0/22:6 *** | 4.3 ± 1.5 | 6.7 ± 0.7 | 14.8 ± 1.0 | 11.5 ± 0.9 |

significant effect of diet, * p < 0.05, *** p < 0.001; significant effect of genotype, ^♦^ p < 0.05.
